# Unveiling promising immunogenic targets in *Coxiella burnetii* through in silico analysis: paving the way for novel vaccine strategies

**DOI:** 10.1186/s12879-023-08904-7

**Published:** 2023-12-21

**Authors:** Mansoor Kodori, Jafar Amani, Ali Ahmadi

**Affiliations:** 1 Molecular Biology Research Center, Systems Biology and Poisonings Institute, Baqiyatallah University of Medical Sciences, Tehran, Iran; 2 Non Communicable Diseases Research Center, Bam University of Medical Sciences, Bam, Iran; 3 Applied Microbiology Research Center, Systems Biology and Poisonings Institute, Baqiyatallah University of Medical Sciences, Tehran, Iran

**Keywords:** *Coxiella burnetii*, Reverse vaccinology, Immunogenic proteins, Q Fever

## Abstract

**Background:**

*Coxiella burnetii*, an intracellular pathogen, serves as the causative agent of zoonotic Q fever. This pathogen presents a significant threat due to its potential for airborne transmission, environmental persistence, and pathogenicity. The current whole-cell vaccine (WCV) utilized in Australia to combat Q fever exhibits notable limitations, including severe adverse reactions and limited regulatory approval for human use. This research employed the reverse vaccinology (RV) approach to uncover antigenic proteins and epitopes of *C. burnetii*, facilitating the development of more potent vaccine candidates.

**Methods:**

The potential immunogenic proteins derived from *C. burnetii* RSA493/Nine Mile phase I (NMI) were extracted through manual, automated RV, and virulence factor database (VFDB) methods. Web tools and bioinformatics were used to evaluate physiochemical attributes, subcellular localization, antigenicity, allergenicity, human homology, B-cell epitopes, MHC I and II binding ratios, functional class scores, adhesion probabilities, protein-protein interactions, and molecular docking.

**Results:**

Out of the 1850 proteins encoded by RSA493/NMI, a subset of 178 demonstrated the potential for surface or membrane localization. Following a series of analytical iterations, 14 putative immunogenic proteins emerged. This collection included nine proteins (57.1%) intricately involved in cell wall/membrane/envelope biogenesis processes (CBU_0197 (Q83EW1), CBU_0311 (Q83EK8), CBU_0489 (Q83E43), CBU_0939 (Q83D08), CBU_1190 (P39917), CBU_1829 (Q83AQ2), CBU_1412 (Q83BU0), CBU_1414 (Q83BT8), and CBU_1600 (Q83BB2)). The CBU_1627 (Q83B86 ) (7.1%) implicated in intracellular trafficking, secretion, and vesicular transport, and CBU_0092 (Q83F57) (7.1%) contributing to cell division. Additionally, three proteins (21.4%) displayed uncharacterized functions (CBU_0736 (Q83DJ4), CBU_1095 (Q83CL9), and CBU_2079 (Q83A32)). The congruent results obtained from molecular docking and immune response stimulation lend support to the inclusion of all 14 putative proteins as potential vaccine candidates. Notably, seven proteins with well-defined functions stand out among these candidates.

**Conclusions:**

The outcomes of this study introduce promising proteins and epitopes for the forthcoming formulation of subunit vaccines against Q fever, with a primary emphasis on cellular processes and the virulence factors of *C. burnetii*.

**Supplementary Information:**

The online version contains supplementary material available at 10.1186/s12879-023-08904-7.

## Background

*Coxiella burnetii*, a significant obligate intracellular bacterium first characterized by Harold Cox and MacFarlane Burnet in 1935, causes Q fever, a zoonotic disease affecting both humans and animals worldwide [1]. Although many individuals remain asymptomatic during infection, Q fever can progress to chronic stages. This can lead to severe health issues, such as hepatitis, endocarditis, vasculitis, lymphadenitis, and chronic fatigue syndrome (CFS) [2]. This pathogen predominantly resides in domestic animals like cattle, sheep, and goats, serving as primary reservoirs. Humans contract the infection through the inhalation of infectious aerosols or the consumption of contaminated food or animal products [3]. Notably, *C. burnetii* can endure harsh environmental conditions, exhibiting resilience to pH levels of 4.5, temperatures of 62 °C for 30 min, and UV irradiation [2].

Throughout history, three major Q fever outbreaks have been documented in Africa (1955), the Netherlands (2007–2010), and French Guiana. This highlights the need to mitigate infection rates through vaccine-centered preventive strategies [4]. Currently, the only available vaccine for Q fever prevention is Q-VAX®, a formalin-inactivated whole-cell vaccine derived from Phase I Henzerling strain [5]. Although effective in providing protection in animals (Coxiellosis; Coxevac vaccine was used), its human application is restricted to Australia due to significant local and systemic responses observed in individuals with prior exposure to the pathogen. This necessitates pre-vaccination serologic screening and intradermal skin testing, leading to delayed vaccination [6]. A substantial proportion (18.3-45.13%) of individuals at high risk of Q fever show detectable levels of anti-*C. burnetii* antibodies, underscoring the importance of vaccinating high-risk groups [7, 8]. Consequently, there is an urgent need to develop a vaccine that ensures potent and lasting protection without adverse effects on recipients. To overcome the challenges of genetic and proteomic manipulation of *C. burnetii* in the laboratory, a more pragmatic approach involves employing bioinformatics analysis to identify specific immunogenic elements. This approach provides valuable insights into the key components responsible for eliciting an immune response, facilitating the development of more effective vaccines [9]. Recent advances in genomic sequencing and organism-specific databases, along with epitope prediction servers relying on protein and nucleotide sequences, have significantly enhanced our understanding of antigenic components of microorganisms [10]. Among these approaches, reverse vaccinology (RV) stands out as an in silico-based procedure to identify immunogenic antigens from complete pathogen genomes. By using RV pipelines, novel or undiscovered antigens can be detected, which might remain unnoticed through traditional methods. Leveraging data from the whole proteome of pathogens, the RV approach employs various bioinformatics tools to determine and analyze potential vaccine candidates’ properties [11]. Using the RV approach, this study aimed to predict the most effective immunological proteins for vaccine candidates against *C. burnetii*. By utilizing an in-silico methodology, novel vaccine candidates will be identified via different bioinformatics tools based on their biological characteristics strongly associated with the defensive immune response.

## Methods

### Retrieval of genomic and proteomic data

The genomic and proteomic sequences of the most relevant *C. burnetii* strains RSA493/Nine Mile Phase I (NMI), which is present in the chloroform-methanol residue (CMR) formulation, were obtained from reputable repositories (Accession number: NC_002971.4), namely the National Center for Biotechnology Information (NCBI) (https://www.ncbi.nlm.nih.gov/nuccore/NC_002971.4) [12] and the non-redundant proteomes section of the Universal Protein Resource (https://www.uniprot.org/taxonomy/227377) [13]. All sequences were retrieved in the widely adopted FASTA format and meticulously preserved for subsequent comprehensive analysis and investigation.

### Virulence factors and literature review

Virulence factors for specific proteins were assessed using the virulence factor database (VFDB) (http://www.mgc.ac.cn/VFs/main.html) [14], and protective antigens were identified by Protegen (https://violinet.org/protegen/) [15]. BLASTp inquiries were executed against server databases, using a threshold of identity and query coverage greater than 30%, to assess protein similarity to Homo sapiens (taxid: 9606). Furthermore, this tool was also applied to avoid any potential interference with the normal microbiota.

### Automated reverse vaccinology

In this step, the reference genome chosen for analysis was the *C. burnetii* strain RSA493/NMI (Accession number: NC_002971.4). The Vaxign database (http://www.violinet.org/vaxign/) was utilized to identify potential immunogenic targets [16]. The selection criteria and thresholds used were as follows: the number of transmembrane helices should not exceed one, the adhesion probability must be greater than 0.51, and the identified targets should show no similarity to proteins found in humans and mice.

### Manual reverse vaccinology

The analysis of the FASTA format of *C. burnetii* strain RSA493/NMI proteome focused on identifying surface-exposed proteins, specifically those present in the outer membrane (OM) and extracellular (EC) regions. These proteins are particularly valuable as targets for vaccination due to their accessibility to antigen-presenting cells. To achieve this, different tools, mainly PSORTb (https://psort.org/psortb) and LocTree3 (https://rostlab.org/services/loctree3/), were utilized [17, 18]. Additionally, the number of transmembrane helices was examined.

### Assessment of antigenicity and allergenicity

Surface-exposed proteins were evaluated using the VaxiJen web server (http://www.ddg-pharmfac.net/vaxijen/VaxiJen/VaxiJen.html). This approach involved converting protein sequences into normal vectors corresponding to the specific properties of the amino acids constituting the sequence. Auto-cross-covariance analysis (ACC) was conducted, and proteins with cutoff scores of 0.5 or higher were identified as potential antigens capable of stimulating immune responses [19]. To assess the allergenicity of the selected proteins, the Algpred online server (https://webs.iiitd.edu.in/raghava/algpred2/batch.html) was utilized, employing a hybrid approach (RF + BLAST + MERCI) [20].

### Physiochemical characteristics of proteins

Various databases were used to analyze protein physicochemical properties. To ascertain specific characteristics such as the number of amino acids, molecular weight, theoretical pI, estimated half-life, aliphatic index, and instability index, the Expasy ProtParam server (https://web.expasy.org/protparam/) was employed [21]. Additionally, the adhesion probability was identified using the Vaxign database (http://www.violinet.org/vaxign2) [16]. To determine the functional class of the proteins, the VICMpred database (https://webs.iiitd.edu.in/raghava/vicmpred/submission.html) was utilized.

### Identification of linear B-Cell epitopes and human MHC I and MHC II binding sites

To enhance the reliability of predictions, two different programs were employed: the Immune Epitope Database (IEDB) (http://tools.iedb.org/main/bcell/) and the BepiPred v2.0 tool (https://services.healthtech.dtu.dk/services/BepiPred-2.0/), using a threshold of ≥ 0.6. The BepiPred tool uses a random forest algorithm trained on epitopes of antibody-antigen protein structures [22]. The IEDB Bepipred Linear Epitope Prediction 2.0 method, with a threshold set at ≥ 0.6, was employed. The ratio of B-cell epitopes to the total number of amino acids was calculated for each protein. The TepiTool server, accessible through the IEDB database (http://tools.iedb.org/tepitool/), was utilized to identify MHC I and MHC II binding sites [23]. Specifically, 27 alleles were selected for MHC I binding site prediction, utilizing a panel of the 27 most frequent A and B alleles, collectively representing over 97% of the global population. The analysis focused on peptides of moderate length, ranging from 8 to 11 amino acids. Conservancy analysis was not included; the NetMHCpan prediction method was used for peptide selection based on a predicted IC50 value of ≤ 50. To predict human MHC II binding sites, a panel of the 26 most frequent alleles was employed, and conservancy analysis was not performed. The IEDB recommended prediction method was applied, and peptide selection was based on the top 5% of predictions. Subsequently, the ratio of MHC I and MHC II binding sites to the total number of amino acids was calculated across all proteins.

### Exploration of tertiary structure and conformational B-cell epitopes

The 3D tertiary structure of putative immunogenic proteins was characterized using the Robetta tool (https://robetta.bakerlab.org/) [24]. The 3D model quality was assessed using the ProSA web server (https://prosa.services.came.sbg.ac.at/prosa.php). This server identifies potential errors in the 3D model [25]. Furthermore, ElliPro (http://tools.iedb.org/ellipro/) was employed to identify conformational B-cell epitopes with a threshold value of ≥ 0.8. The predicted conformational B-cell epitopes were visualized on the surface of each protein in distinct colors by PyMOL software (https://pymol.org/2/).

### Quartile scoring method

The chosen proteins underwent analysis using the quartile method scoring with ten indicators, comprising functional class (virulence, cellular process, metabolic molecule, and unknown), antigenicity, hydropathy index, instability index, MHC I binding site ratio, MHC II binding site ratio, linear B-cell epitope, conformational B-cell epitope, B-cell epitope ratio and adhesion probability value. The cumulative score obtained from each indicator for each protein was considered the final score. Proteins scoring ≥ 25 points were deemed appropriate immunogenic targets. The Quartile method quantifies data spread by segmenting its distribution into lower, median, and upper quartiles, creating four distinct intervals. This approach allows for a reasoned selection of targets, as it simultaneously assesses proteins using multiple unweighted criteria [26].

### Identification of conserved domains and protein–protein interaction networks

The major protein domains were identified using two specific resources: the Conserved Domain Database (CDD), accessible at https://www.ncbi.nlm.nih.gov/Structure/cdd/cdd.shtml and part of NCBI’s Entrez query system, provides annotations of protein sequences with the location of conserved domains [27]. Additionally, EggNOG, available at http://eggnog5.embl.de/#/app/home, serves as a hierarchical orthology resource with functional and phylogenetic annotations derived from a comprehensive dataset of 5090 organisms and 2502 viruses [28]. Moreover, exploration of interactions between putative vaccine candidates with unknown functions and other *C. burnetii* proteins aimed to infer their potential roles using the STRING database, accessible at https://string-db.org/. In this analysis, connection scores greater than 0.5 were considered significant and considered for further evaluation.

### Molecular docking and immune simulation

The study delved deeply into the intricate process of molecular docking, investigating the strength of binding interactions between putative immunogenic proteins and human Toll-Like Receptors (TLRs), specifically TLR-1 (PDB: 2Z7X), TLR-2 (PDB: 2Z7X), TLR-4 (PDB: 3FXI), and TLR-6 (PDB: 379 A). To conduct this investigation, advanced computational tools were employed, including the ClusPro 2.0 server (https://cluspro.bu.edu/login.php) and PatchDock version Beta 1.3 (http://bioinfo3d.cs.tau.ac.il/PatchDock/php.php) [29, 30]. Furthermore, prognostications of immune responses to these potential targets were achieved through C-ImmSim (https://kraken.iac.rm.cnr.it/C-IMMSIM/index.php).

## Results

### Identification of relevant proteins

**Fig. 1:**
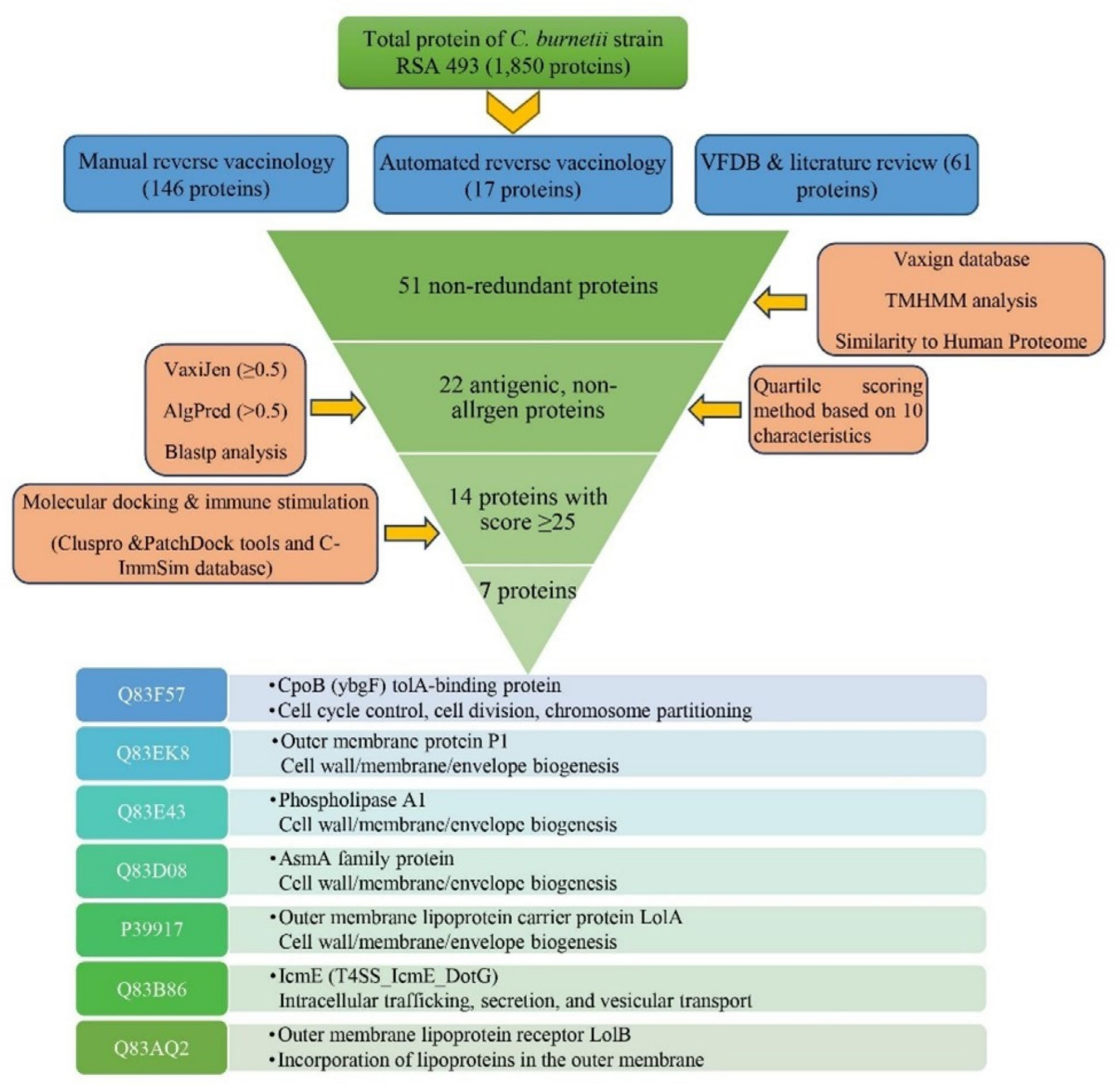
Workflow illustrating the process of identifying potential novel immunogenic targets against *C. burnetii* using a RV approach

The workflow depicting the process for identifying novel immunogenic targets through the RV method is presented in Fig. 1. For a comprehensive approach, multiple strategies were employed to extract 178 proteins for subsequent analysis (Supplementary File 1). A thorough literature review and extensive exploration of the VFDB led to the acquisition of 61 proteins. Additionally, the automated reverse vaccinology (ARV) approach contributed 17 proteins. Notably, the most substantial contribution emerged from our rigorous manual reverse vaccinology (MRV) efforts, revealing an impressive set of 146 proteins.

**Fig. 2:**
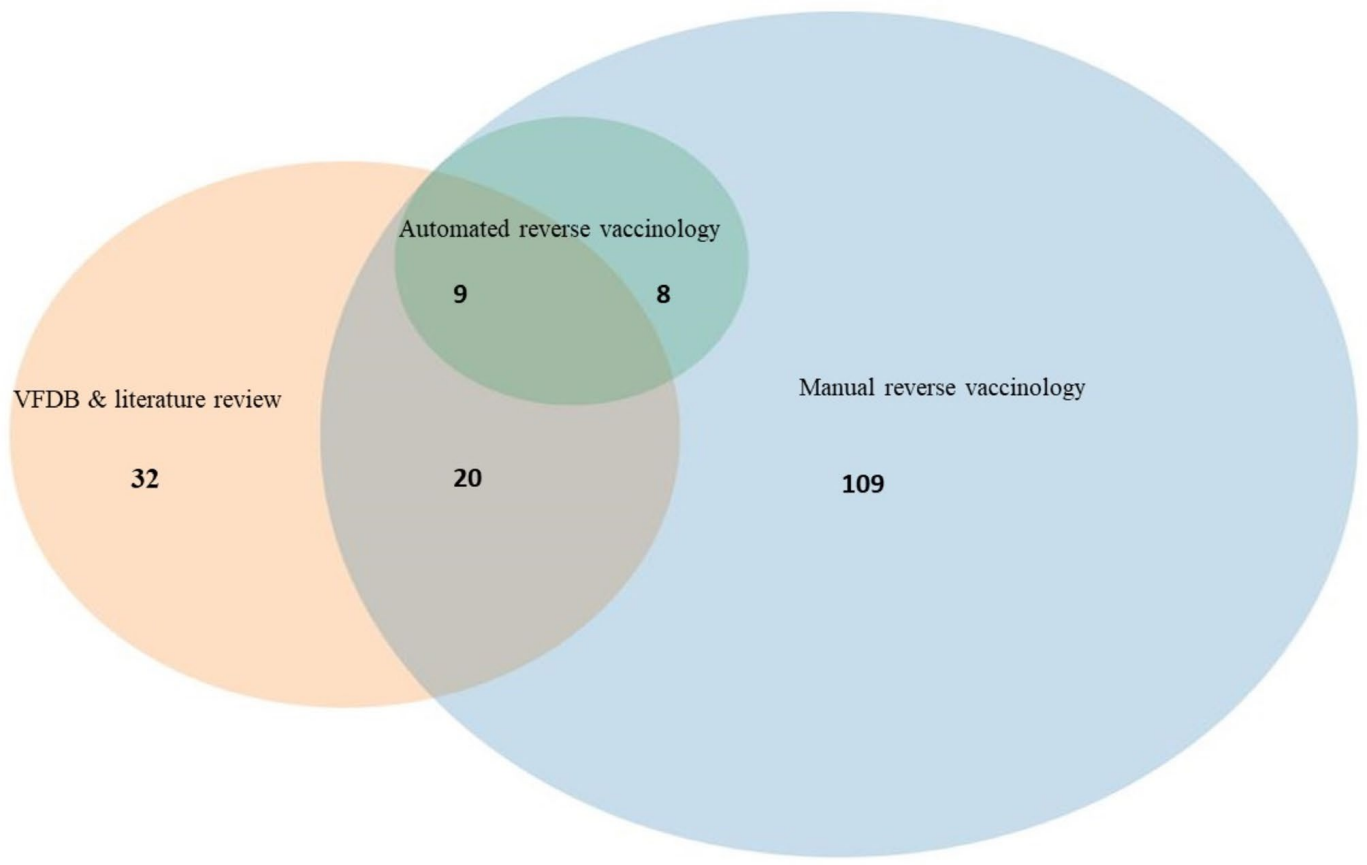
Protein distribution across three distinct methods: VFDB, MRV, and ARV. Notably, only nine proteins were identified shared by all three methods. The MRV approach yielded the highest number of identified proteins, demonstrating a close alignment with the ARV method results. For a comprehensive understanding of these findings, Supplementary Table 1 offers further details

The outcomes obtained from the ARV approach were entirely consistent with those resulting from the MRV process. Supplementary File 2 (Table S1) provides comprehensive and detailed information concerning the aforementioned proteins. Interestingly, only nine proteins were consistently identified across all three methods (Accession number:NP_819641.1, NP_819109.1, NP_820609.2, NP_820254.1, NP_819350.1, NP_819143.2, NP_819783.2, NP_819950.2, and NP_820613.2. This limited overlap underscores the distinct and complementary nature of each method, highlighting the importance of employing a diverse array of techniques for comprehensive protein identification. Figure 2 visually represents the distribution of proteins among the three approaches, showcasing both the common proteins and those unique to each method.

### Assessment of homology, adhesion probability, and transmembrane helices in proteins

According to PSI-BLAST and Vaxign analyses, thirteen proteins (7.3%) exhibited significant homology with Homo sapiens (taxid: 9606). Furthermore, among the 178 proteins, 57 (32%) displayed an adhesion probability score exceeding 0.51, resulting in the exclusion of 121 proteins from the analysis. Using both the Vaxign and TMHMM online servers, two or more transmembrane helices were identified in 15 proteins (8.4%). Consequently, 51 non-redundant proteins (28.65%) were selected for further analysis.

### Assessment of the antigenicity and allergenicity of proteins

After excluding redundant proteins, 51 secreted and surface-exposed proteins were identified. The subset of proteins exposed on the surface and secreted underwent VaxiJen analysis, which accurately identified 58.82% (30/51) of the proteins as antigenic. Among these, a closer assessment revealed that 26.6% (8/30) of proteins were identified as allergens and excluded from further investigations. A thorough BlastP analysis was performed, comparing the putative immunogenic proteins against *Lactobacillus* proteomes. The results conclusively indicate that the putative immunogenic proteins exhibit no detectable similarity or resemblance to the proteins present in *Lactobacillus* species proteomes. Then, 22 antigenic non-allergen proteins were carefully selected for in-depth analysis and subsequent investigation.

### Analysis of physiochemical characteristics of putative immunogenic proteins

Through conserved domain analysis and text mining, the selected proteins were assigned to four different functional classes. Out of 22 proteins, 7 are classified as cellular processes, accounting for 31.8% of the proteins. The next most abundant categories were proteins related to metabolic molecules and virulence factors, each comprising 6 out of 22 (27.2%) proteins. Additionally, 3 (13.6%) proteins have unknown functions. The estimated half-life for all proteins was determined to be 30 h in mammalian reticulocytes (in vitro), over 20 h in yeast (in vivo), and over 10 h in *E. coli* (in vivo). Further physicochemical characteristics of these proteins can be found in Supplementary File 3.

### Characterization of immunogenic epitopes

**Fig. 3:**
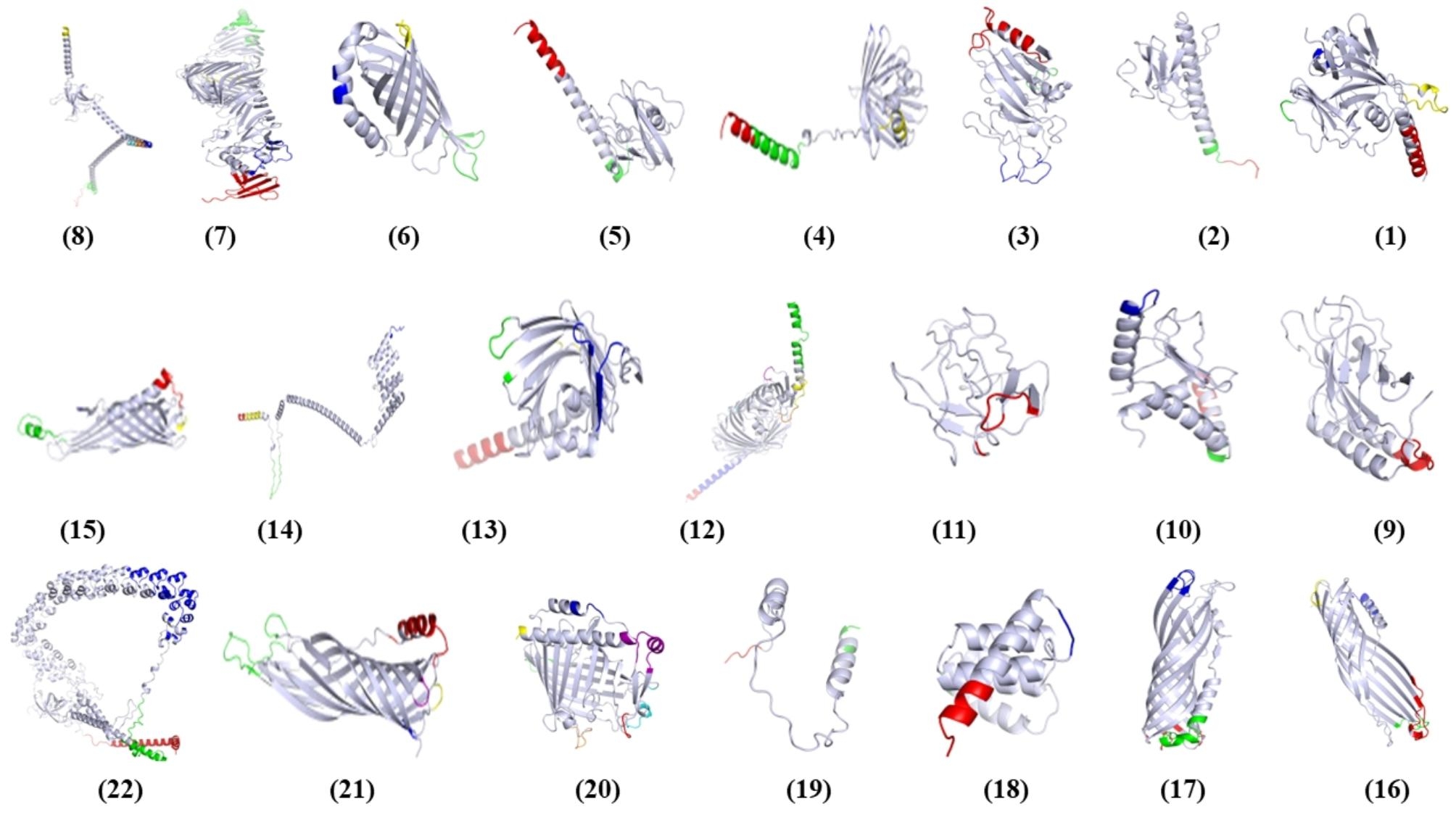
This study investigated conformational epitopes located on specific proteins’ surfaces. To predict tertiary protein structures, the Robetta Web tool was utilized. Subsequently, PyMOL software was employed to identify surface-exposed epitopes in the proteins’ 3D structures. The color and the score of each protein have shown in Supplementary Table S2. The accession numbers for the selected proteins are as follows: (1) NP_821052.2, (2) NP_821049.1, (3) NP_819762.1, (4) NP_820808.1, (5) NP_820094.2, (6) NP_820583.1, (7) NP_819243.1, (8) NP_819755.1, (9) WP_010891173.1, (10) NP_820596.1, (11) YP_002332945.1, (12) NP_819951.2, (13) NP_820185.1, (14) NP_819144.2, (15) NP_820793.1, (16) NP_820398.1, (17) NP_820396.2, (18) NP_820832.2, (19) NP_821009.2, (20) NP_819523.1, (21) NP_819354.2, and (22) NP_820609.2.

The study involved a thorough analysis of 22 proteins, during which the number of linear and conformational B-cell epitopes, the ratio of B-cell epitopes, and the MHC I and MHC II binding sites ratio were determined. These results have been compiled and are available in Supplementary File 3. Additionally, Supplementary File 4 (Table S2) contains the detailed sequences of both linear (Including 27 most frequent A & B alleles) and conformational B-cell epitopes (Including 26 most frequent alleles) for each of the proteins studied. The surface-exposed B-cell epitopes of the novel immunogenic targets were shown in Fig. 3, and the sequence and corresponding color of each epitope were meticulously detailed in Supplementary Table S2.

### Identification of optimal immunogenic candidates using the quartile scoring method and analysis of physicochemical properties

**Fig. 4:**
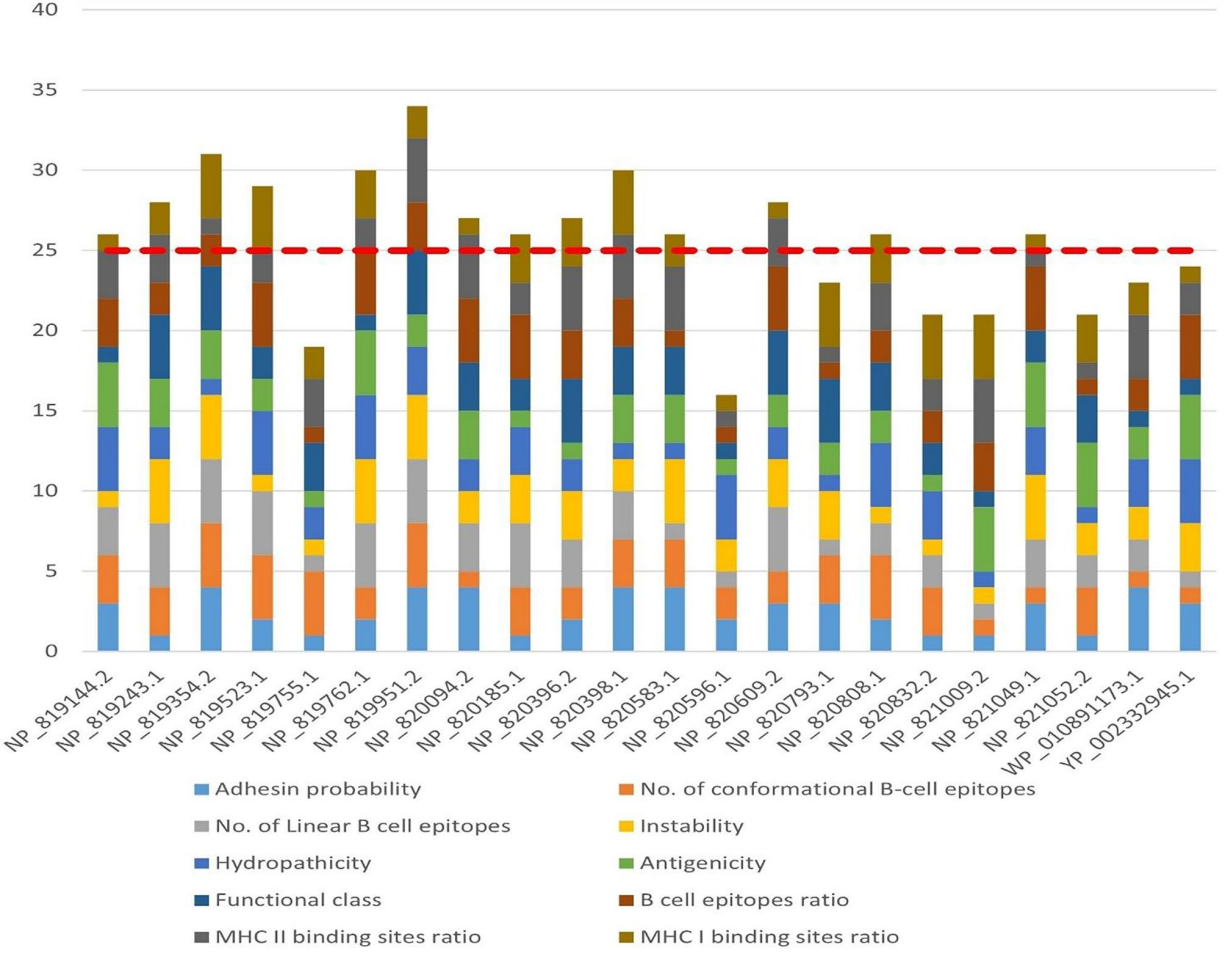
Comparative analysis of potential immunogenic targets against *C. burnetii* using quartile scoring

By employing the quartile scoring method, which incorporates ten distinct features, a meticulous selection process resulted in the identification of 14 out of 22 proteins. These proteins displayed scores equal to or greater than 25. The assigned individual scores for each protein were as follows: NP_819144.2 (26), NP_819243.1 (28), NP_819354.2 (31), NP_819523.1 (29), NP_819762.1 (30), NP_819951.2 (34), NP_820094.2 (27), NP_820185.1 (26), NP_820396.2 (27), NP_820398.1 (30), NP_820583.1 (26), NP_820609.2 (28), NP_820808.1 (26), and NP_821049.1 (26). Figure 4 provides a visual representation. In addition, Supplementary File 3 provides detailed descriptions of the physicochemical characteristics of the 14 selected proteins.

### Protein domain search and protein-protein interactions

**Fig. 5:**
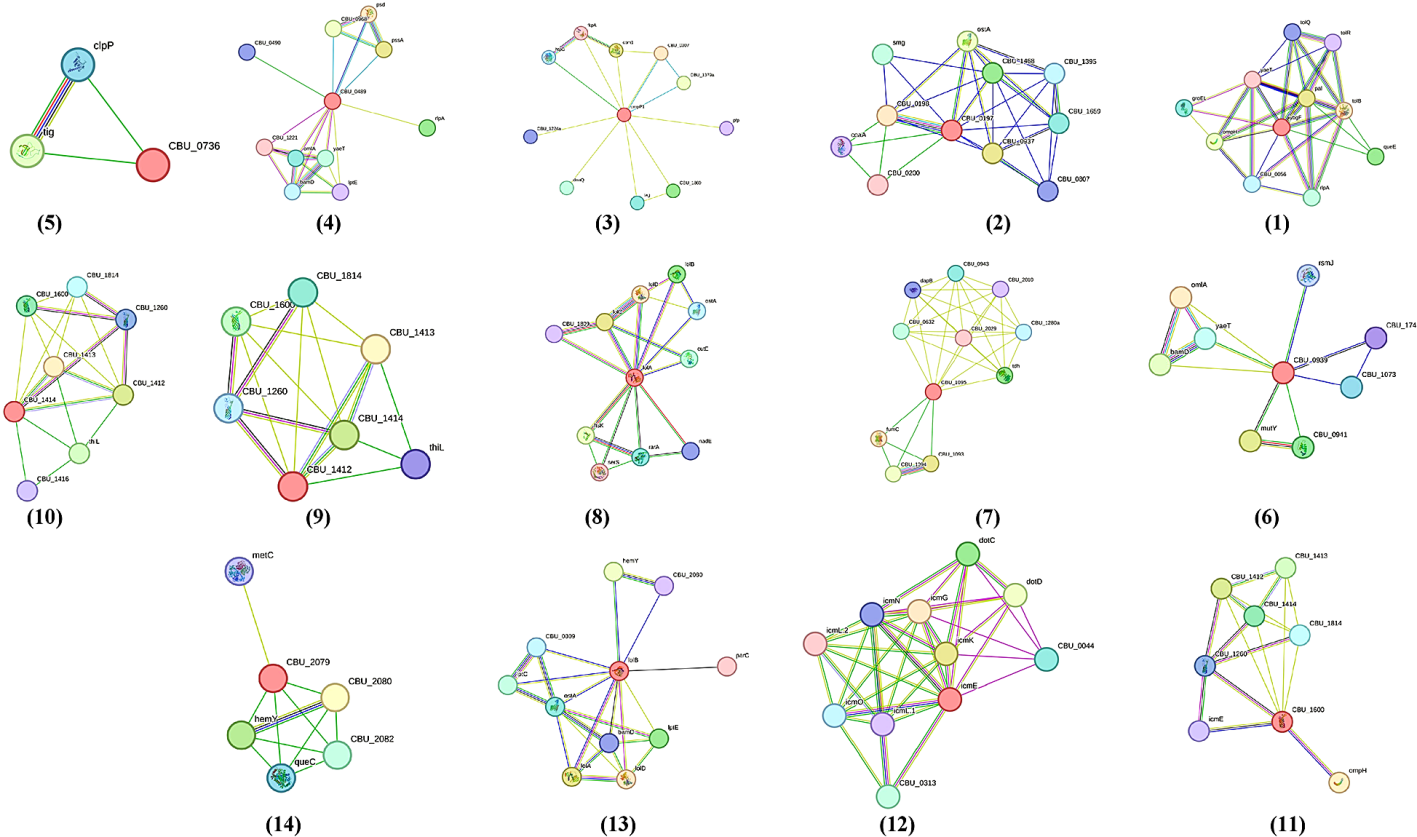
Protein-protein interaction networks of proteins with other proteins of *C. burnetii*. (1) Q83F57, (2) Q83EW1, (3) Q83EK8, (4) Q83E43, (5) Q83DJ4, (6) Q83D08, (7) Q83CL9, (8) P39917, (9) Q83BU0, (10) Q83BT8, (11) Q83BB2, (12) Q83B86, (13) Q83AQ2, (14) Q83A32.

The analyses conducted using CDD and EggNOG highlighted the significant involvement of nine selected proteins (64.2%) in cell wall/membrane/envelope biogenesis. These proteins have been identified as Q83EW1 (Hypothetical outer membrane protein), Q83EK8 (Omp1), Q83E43 (Phospholipase A1), Q83D08 (AsmA), P39917 (LolA), Q83BU0 (Hypothetical exported protein), Q83BT8 (Hypothetical exported protein), Q83AQ2 (LolB), and Q83BB2 (Heat resistant agglutinin). Notably, Q83B86 (icmE) is categorized under T4SS_IcmE_DotG, and it is associated with critical functions in intracellular trafficking, secretion, and vesicular transport. Further insights reveal that Q83F57 (CpoB) plays a role in cell division processes. In contrast, the functions of three proteins, specifically Q83DJ4 (Hypothetical exported protein), Q83CL9 (Hypothetical exported protein), and Q83A32 (Uncharacterized protein), could not be ascertained through these analyses (Table 1). These results contribute to an enhanced understanding of the intricate roles played by these proteins in fundamental biological processes. Figure 5 illustrates the findings derived from the analysis of protein-protein interactions using the STRING database.

**Table 1:** Information regarding the conserved domains of the 14 putative immunogenic proteins against *C. burnetii* was gathered from the NCBI Conserved Domain Database (CDD), EggNOG, and STRING databases

UniProt ID (Locus_tag)	CDD	EggNOG	STRING
Q83F57 (CBU_0092)	Periplasmic TolA-binding protein (CpoB)	Function unknown (protein trimerization)	Mediates coordination of peptidoglycan synthesis and outer membrane constriction during cell division.
		Cell cycle control, cell division, chromosome partitioning (Mediates coordination of peptidoglycan synthesis and outer membrane constriction during cell division)	
Q83EW1 (CBU_0197)	Hypothetical outer membrane protein	Cell wall/membrane/envelope biogenesis	Hypothetical outer membrane protein
		Function unknown	
Q83EK8 (CBU_0311)	Outer membrane porin P1	omp1	Outer membrane protein P1
		Cell wall/membrane/envelope biogenesis (Has lipid A 3-O-deacylase activity. Hydrolyzes the ester bond at the 3 position of lipid A, a bioactive component of lipopolysaccharide (LPS), thereby releasing the primary fatty acyl moiety)	
Q83E43 (CBU_0489)	Phospholipase A1	Cell wall/membrane/envelope biogenesis (1-acyl-2-lysophosphatidylserine acylhydrolase activity)	Phospholipase A1; Hydrolysis of phosphatidylcholine with phospholipase A2 (EC 3.1.1.4) and phospholipase A1 (EC 3.1.1.32) activities. Belongs to the phospholipase A1 family.
		Phospholipase	
Q83DJ4 (CBU_0736)	Hypothetical protein (Unknown)	No orthologs found	Hypothetical exported protein
Q83D08 (CBU_0939)	Uncharacterized protein involved in outer membrane biogenesis (ASMA)	Cell wall/membrane/envelope biogenesis (Protein involved in outer membrane biogenesis)	AsmA family
		AsmA family	
Q83CL9 (CBU_1095)	Hypothetical exported protein (Unknown)	No orthologs found	Hypothetical exported protein
P39917 (CBU_1190)	Outer membrane lipoprotein carrier protein LolA; pfam03548	Cell wall/membrane/envelope biogenesis (Participates in the translocation of lipoproteins from the inner membrane to the outer membrane.)	Outer-membrane lipoproteins carrier protein (Participates in the translocation of lipoproteins from the inner membrane to the outer membrane).
Q83BU0 (CBU_1412)	Hypothetical exported protein	Cell wall/membrane/envelope biogenesis (Has lipid A 3-O-deacylase activity. Hydrolyzes the ester bond at the 3 position of lipid A, a bioactive component of lipopolysaccharide (LPS), thereby releasing the primary fatty acyl moiety)	Hypothetical exported protein.
Q83BT8 (CBU_1414)	hypothetical exported protein (*Legionella pneumophila* major outer membrane protein precursor)	Cell wall/membrane/envelope biogenesis (Has lipid A 3-O-deacylase activity. Hydrolyzes the ester bond at the 3 position of lipid A, a bioactive component of lipopolysaccharide (LPS), thereby releasing the primary fatty acyl moiety)	Hypothetical exported protein
Q83BB2 (CBU_1600)	Heat resistant agglutinin	Cell wall/membrane/envelope biogenesis (Has lipid A 3-O-deacylase activity. Hydrolyzes the ester bond at the 3 position of lipid A, a bioactive component of lipopolysaccharide (LPS), thereby releasing the primary fatty acyl moiety)	Uncharacterized protein
Q83B86 (CBU_1627)	IcmE (T4SS_IcmE_DotG)	Function unknown (protein homooligomerization)	IcmE
		Intracellular trafficking, secretion, and vesicular transport (multi-organism process)	
Q83AQ2 (CBU_1829)	Outer membrane lipoprotein receptor (LolB)	Protein transport (lolB)	Outer-membrane lipoprotein; Plays a critical role in the incorporation of lipoproteins in the outer membrane after they are released by the LolA protein.
		Plays a critical role in the incorporation of lipoproteins in the outer membrane after they are released by the LolA protein	
Q83A32 (CBU_2079)	Hypothetical protein (Unknown)	No orthologs found	Uncharacterized protein

### Docking with TLRs and immune simulation

Utilizing ClusPro 2.0, a molecular docking analysis was conducted to assess the interactions between the putative immunogenic proteins and TLR-1, TLR-2, TLR-4, and TLR-6. The results revealed a notable propensity for robust binding affinity across all putative immunogenic proteins towards their respective TLRs. This was indicated by significantly low mean binding energy values: -1070.0 kcal/mol for TLR-1, -1111.2 kcal/mol for TLR-2, -1235.4 kcal/mol for TLR-4, and − 1210.5 kcal/mol for TLR-6. The interaction of these proteins with TLR-1, 2, 4 and 6 is presented in Supplementary File 6 (Figure S1, S2, S3, S4). However, upon analysis of the first ranking scores from PatchDock, it was discovered that some proteins demonstrated less favorable negative atomic contact energy (ACE) values when interacting with TLR-1 (Q83EK8: -83.81; Q83BT8: -3.57), TLR-2 (Q83F57: -263.20), TLR-4 (Q83E43: -101.47), and TLR-6 (Q83EK8: -8.70) (Supplementary File 5).

Based on C-ImmSim results, all putative immunogenic proteins were found to induce outstanding cytokines (IL-2 and IFN-γ) and T cell stimulation, including T CD^4+^ and T CD^8+^ Despite the high induction in B cell isotype IgM, nine putative portions demonstrated stimulation of B cell isotype IgG1 population (Table 2).

**Table 2:** Immune simulation of 14 putative immunogenic proteins via C-ImmSim web server

UniProt ID	B-cell population (cells/mm3)			T-cell populations (cells/mm3)		IL-2 (ng/ml)	IFN-γ (ng/ml)
	IgM,	IgG1	IgG2	CD4 T-helper lymphocytes	CD8 T-cytotoxic lymphocytes		
Q83F57	460	10	0	4700	1135	260,000	380,000
Q83EW1	480	5	0	3500	1130	190,000	370,000
Q83EK8	480	5	0	4700	1135	310,000	380,000
Q83E43	480	0	0	3900	1135	220,000	360,000
Q83DJ4	470	15	0	4600	1125	310,000	370,000
Q83D08	480	0	0	3600	1132	190,000	370,000
Q83CL9	470	0	0	2300	1130	80,000	360,000
P39917	470	0	5	4200	1130	235,000	390,000
Q83BU0	470	10	0	5000	1132	330,000	390,000
Q83BT8	480	5	0	4400	1132	260,000	370,000
Q83BB2	480	0	0	3500	1132	175,000	380,000
Q83B86	470	5	0	4200	1132	250,000	380,000
Q83AQ2	480	15	0	4700	1132	300,000	390,000
Q83A32	480	10	0	5100	1132	340,000	390,000
**Average**	475	5.7	0.35	4171.4	1131.7	246428.6	377142.9

### Shortlist of selected proteins

Given the consistent and congruent outcomes derived from the comprehensive suite of molecular docking analyses, which were further corroborated by the concurrent elicitation of immune responses encompassing the entire ensemble of 14 examined proteins, it is a plausible and rational conjecture to accord serious consideration to each of these identified proteins as viable contenders for inclusion in the roster of potential vaccine candidates. These proteins possess the inherent capacity to orchestrate and stimulate protective immune defenses against the pathogen *C. burnetii*. However, among these candidates, seven proteins with known functions stand out as strong and promising options. These proteins are identified by their UniProt codes: Q83F57 (YbgF), Q83EK8 (outer membrane protein P1), Q83E43 (Phospholipase A1), Q83D08 (an ASMA protein), P39917 (LolA), Q83B86 (IcmE), and Q83AQ2 (LolB). They showed potential to trigger strong immune responses against *C. burnetii* and are thus considered potential targets for immunization.

## Discussion

Traditional vaccine methods, rooted in Pasteur’s principles, face challenges in combatting infectious diseases. Many existing vaccines were created using older methods [31, 32]. Eradicating infectious diseases requires modern vaccine technology, offering improved efficacy, safety, scalability, and control. Whole genome sequences (WGS) have transformed our understanding of pathogens, guiding the development of protective vaccines [33]. Reverse vaccinology (RV) is an innovative approach that involves dissecting the entire genomic landscape of microbial pathogens, specifically *C. burnetii* in this study, to identify promising antigenic vaccine candidates [34]. This study entailed a comprehensive analysis of the proteome of *C. burnetii* strain RSA493/NMI with the aim of identifying potential vaccine candidates against zoonotic Q fever.

Over the past six decades, developing effective vaccines against Q fever has remained a significant challenge. Q-VAX®, the only licensed vaccine for Q fever, is derived from the formalin-inactivated Phase 1 antigenic state [35]. However, its use is limited to Australia due to hypersensitivity risks. Phase II whole-cell vaccine (WCV) demonstrates limited effectiveness as a standalone vaccine material, providing insufficient protection in numerous animal disease models. Its application to sheep primarily serves as a colonization model, aimed at reducing infectious materials. Furthermore, experimental evidence has shown that Phase II WCV induces a level of reactogenicity in guinea pigs comparable to that of Phase I WCV [36]. To address this, alternative vaccine modalities are urgently needed to combat Q fever global burden [37]. Historically, a WCV was developed through formaldehyde inactivation, demonstrating effectiveness against laboratory-acquired infections but causing adverse reactions. Initial efficacy trials showed protection, especially in individuals with pre-existing immunity [36]. In response to these findings, comprehensive pre-vaccination assessments have been integrated into the Q-Vax® vaccination protocol, including serological analyses, thorough patient history evaluations, and skin tests [38]. Subsequently, further refinements have been introduced to enhance the safety profile of the initial WCVs and reduce adverse reactions [38]. The CMR preparation aimed to enhance safety and immunogenicity but remains unapproved for widespread use [39]. The advent of phase II lipopolysaccharides (LPS) in WCV led to the replacement of Phase II with Phase I vaccines. Concerns regarding outbreaks, bioterrorism, and occupational exposure underscore the need for an improved Q fever vaccine [36, 40].

In this study, a triad of distinct bioinformatics methodologies (MRV, ARV, and VDBF) was employed, accompanied by a comprehensive literature review, to investigate the genome and proteome of the RSA493/NMI strain. The primary goal was to identify novel immunogenic proteins. The analysis revealed an expected list of 178 membrane or surface proteins, which are significant for their potent immunogenic and protective attributes as virulence determinants, as well as their potential to induce antibody recognition. To ensure a comprehensive exploration of potential vaccine candidates, a variety of subcellular localization servers were employed, minimizing the risk of overlooking critical surface-exposed proteins [41]. Following rigorous analysis, 14 proteins were identified as strong vaccine candidates for *C. burnetii* due to consistent molecular docking results and concurrent immune responses. In the context of diagnostic and vaccine development, four proteins, namely Q83F57 (YbgF), Q83EK8 (Omp1), P39917 (LolA), and Q83b86 (IcmE), had been previously introduced. Notably, seven proteins with known functions stand out: OmP1 (Q83EK8), Phospholipase A1 (Q83E43), ASMA protein (Q83D08), LolA (P39917), IcmE (Q83B86), and LolB (Q83AQ2). These proteins exhibit the potential to evoke robust immune responses against *C. burnetii* in in-silico conditions and are regarded as potential targets for immunization. OmP1, a member of the *Coxiella* porin p1 (cpp1) family, has a weight of 26.77 kDa. It is highly expressed in the metabolically active large cell variant (LCV) stage while being down-regulated in the small cell variant (SCV) stage [42]. OmP1 is conserved across multiple strains, including RSA 331/Henzerling II. Its potential as a subunit vaccine candidate is based on its alignment with cell surface attributes, susceptibility to iodination reactions, resistance to detergent solubilization, reactivity in immunosorbent assays, and abundance [43]. In the study conducted by Varghees, Sunita,et *al*., a comprehensive examination of OmP1 was meticulously performed across various stages of the pathogen’s life cycle. The investigation specifically concentrated on distinguishing between metabolically active LCVs and SCVs. The findings from this analysis revealed the conservation of OmP1 across multiple strains of *C. burnetii*, thus emphasizing its crucial role as an integral component of the pathogen’s outer membrane. Furthermore, it suggests OmP1 efficacy in enhancing clearance from spleens of infected mice, along with early recognition during infection [42]. Li, Qingfeng, et al. explored the potential of the OmP1-HspB fusion protein to induce protective immunity against Q fever. Using an animal model, they evaluated the fusion protein’s immunogenicity and protective efficacy. Immunization with the fusion protein induced robust immune responses, including specific antibody production and cellular immunity activation. It resulted in reduced splenic weights, decreased *Coxiella* loads, and enhanced survival rates compared to immunization with OmP1 or HspB alone. The fusion protein also stimulated strong T-cell responses, suggesting its potential for cell-mediated immunity against *C. burnetii*. These findings align with our results, highlighting OmP1 potential as an effective immunogenic vaccine candidate against Q fever [44]. Phospholipase A1, an immunogenic protein for *C. burnetii*, is an outer membrane enzyme in the outer membrane phospholipase A (OMPLA) family that hydrolyzes phosphatidylcholine by using a Ca2 + ion binding site to remove fatty acids from the 2-position of the substrate [45]. In *Escherichia coli*, it plays a crucial role in bacteriocins secretion, which enhances the permeability of the outer membrane, facilitating the semi-specific release of these antimicrobial compounds. A speculative hypothesis proposes that OMPLA may have a role in conferring bacterial tolerance to organic solvents. In our study, Phospholipase A1 was introduced as an immunogenic protein under in silico conditions [46]. Recent studies on *C. burnetii* have unveiled the role of an outer membrane phospholipase A (PldA) in orchestrating the accumulation of lyso-phosphatidylethanolamine and free fatty acids within the SCV. Furthermore, PldA plays a crucial role in supporting pathogen growth in the challenging macrophage environment, which shares similarities with lysosomes. These findings collectively underscore PldA remarkable adaptability to flourish within macrophages and its pivotal role in the integral lipid remodeling processes crucial to the pathogen’s development [47]. The central virulence factor of *C. burnetii* is the Dot/Icm T4SS, which exhibits structural similarity to the system identified in *Legionella pneumophila*. This apparatus plays a critical role in intracellular replication, the genesis of *Coxiella*-containing vacuoles (CCVs), the transport of effector molecules, and the regulation of host cell apoptosis [48]. Another immunogenic protein identified in our study is intracellular multiplication protein IcmE, which displays homology with *L. pneumophila* VirB10 and is conserved among strains [49]. The Dot/Icm T4SS functions as a conduit for the delivery of a repertoire of effector proteins into the host cell cytoplasm. These effectors play a critical role in the survival of the host cell and the development of CCVs. The biogenesis of CCVs relies on the T4SS-mediated redirection of numerous intracellular trafficking pathways, essential for providing membranes and nutrients vital for bacterial replication [50]. Recently, it was reported that IcmE, serves as the basis for new Q fever diagnostic and vaccine development [51]. Hence, conducting further research on this protein as a potential vaccine candidate or for the identification of *C. burnetii* could be of significant importance. During the investigation, additional immunogenic proteins have been identified, notably the outer-membrane lipoprotein carrier protein LolA and the lipoprotein LolB. LolA, operating as a transmembrane transporter, fulfills a pivotal role in translocating lipoproteins from the inner to the outer membrane (involved in transport/trafficking) [52]. Through its synergy with LolB, LolA expedites the transportation of lipoproteins, specifically those destined for the outer membrane, from the inner membrane to the outer membrane. The collaborative actions of these proteins, encompassing LolA, LolB, LolC, LolD, and LolE, are indispensable for the precise sorting and localization of lipoproteins within the membrane. It is noteworthy that these proteins exhibit a high degree of conservation among Gram-negative bacteria [53, 54]. In study of Gerlach, C., et al., LolA was introduced as a basis for new Q fever diagnostic and vaccine development [51]. Zhang et al. embarked on an investigation of immunodominant antigens in NMI strain-infected mice. Their research unveiled a spectrum of antigens, each with varying molecular weights. Employing DNA library expression and recombinant plasmids, they successfully identified fifty-four immunoreactive proteins spanning a range from 14 to 60 kDa. However, it became evident that these cloned antigens lacked substantial protective efficacy. This underlines the pressing necessity for more effective candidates in the development of a Q fever vaccine [55]. In another study, protein microarrays were used to differentiate between chronic and convalescent phases of Q fever using sera from Q fever patients. This investigation brought to light an array of seroreactive antigens, including the surface-exposed outer membrane protein Com1. Remarkably, convalescent sera from both murine and human sources exhibited distinct seroreactive proteins [56]. These discoveries underscore the potential importance of T cell antigens in triggering vaccine-induced immunity against *C. burnetii*, extending beyond humoral responses. Nonetheless, the potential overlap in antigenic epitopes between convalescent and vaccinated sera calls for meticulous further investigation [57]. The identification of human T-cell-specific antigens has been a significant breakthrough. Recent research has pinpointed epitopes linked to adaptive immunity in response to natural Q fever infections. Particularly striking were the robust and enduring T-cell immune responses observed in the Netherlands between 2007 and 2010. These responses were associated with HLA class II epitopes and demonstrated the ability to generate long-lasting immunity, persisting for over four years. Notably, HLA class II epitopes consistently exhibited higher reactivity in comparison to their class I counterparts. These findings highlight the potential link between HLA class II epitopes and protective human responses against Q fever, underscoring the role of T-cell-mediated immunity in countering the infection [58]. A comprehensive approach has underscored the crucial role of phase I LPS in eliciting protective responses against whole cell vaccines in mice. Monoclonal antibodies (mAbs) targeting phase I LPS have proven effective in preventing *C. burnetii* infection, serving as a pre-treatment strategy. This reinforces the direct involvement of phase I LPS epitopes in fostering protective responses against *C. burnetii* infection [59]. Exploring subunit vaccines, research has revealed that purified OmP1 confers a degree of protective immunity upon intraperitoneal challenge, surpassing the efficacy of mock-vaccinated mice. Nevertheless, an independent study utilizing a BALB/c murine vaccine-challenge model reported the absence of protective efficacy among various recombinant proteins, including Peptidyl-prolyl cis-trans isomerase Mip, OmP1, and P28. These findings underscore the variability in protective capacities inherent to specific recombinant proteins in the context of Q fever vaccination [55]. Addressing adverse reactions associated with Q-VAX®, Fratzke et al., designed a subunit vaccine incorporating several proteins, including Com1, sucB, LemA, OmpH, CBU0307, and Q83D52, combined with TLR triagonist adjuvants. Trials in guinea pigs demonstrated that these subunit vaccines not only induced protection but also mitigated local adverse effects when compared to a whole cell vaccine. Another study revealed that a subunit vaccine, enhanced by the TLR4_7_9 triagonist adjuvant, exhibited protective efficacy while minimizing local adverse reactions. This suggests that the TLR triagonist platform could be considered an alternative strategy for protection against *C. burnetii* infection [60, 61]. Furthermore, Chen et *al*., harnessed bioinformatics predictions to examine seven proteins that elicited antibody responses, leading to significant IFN-γ production post-vaccination. The study identified eight distinct epitopes derived from four different proteins: DNA-3-methyladenine glycosylase, Hypothetical exported lipoprotein, Com1, and type IV secretion DotB protein. These epitopes constituted 8% of the overall CD4 + T cells responsible for IFN-γ production following vaccination, offering valuable tools for monitoring and analyzing immune responses against *C. burnetii* [60, 62]. In our study, a bioinformatics approach led us to the identification of YbgF (Q83F57), known as the cell division coordinator protein YbgF, as a potential immunogenic protein. YbgF plays a vital role in maintaining the outer membrane’s integrity and coordinating peptidoglycan synthesis and the constriction of the outer membrane during cell division. Through Protein Microarray analysis conducted on Acute and Chronic Q Fever, it was determined that YbgF is present in both phases of *C. burnetii*. Subsequent experiments employing *E. coli* mutants lacking functional tol-pal proteins exhibited a loss of outer membrane integrity, leading to increased sensitivity to drugs and detergents, leakage of periplasmic contents, and the formation of outer membrane vesicles [56]. Additionally, Xiong, Xiaolu, et al., utilized a combination of 2D-PAGE, immunoblotting, and MALDI-TOF-MS to identify 20 seroreactive proteins in *C. burnetii*, where YbgF stood out as a prominent seroreactive antigen. Their findings suggest the potential of these proteins as serodiagnostic markers for Q fever [63]. Our investigation, along with data from the VIOLIN server, highlighted both YbgF and OmP1 as immunodominant antigens of *C. burnetii*. In a parallel study, a recent investigation utilized online tools to predict B cell, T cell, and IFN-γ epitopes within OmP1. Our investigation, alongside data from the VIOLIN server, highlighted both YbgF and OmP1 as immunodominant antigens of *C. burnetii*. Similar to our study, a recent investigation employed online tools to predict B cell, T cell, and IFN-γ epitopes located within OmP1 and YbgF. Subsequently, the epitopes with the highest rankings, in conjunction with Heparin-Binding Hemagglutinin as an adjuvant, were employed to construct a polyepitope fusion protein vaccine. This engineered vaccine showcases significant promise as a robust candidate in the battle against *C. burnetii* [64]. Likewise, other study identified HLA class II T-cell epitope clusters unique to *C. burnetii*. Notably, the cluster containing YbgF was computationally predicted to exhibit broad binding affinity across various HLA-DR types. This cluster also triggered immunogenic responses in transgenic mice expressing human HLA-DR3 (tgHLA-DR3), along with reactivating enduring memory responses in individuals with a history of natural exposure to the pathogen [44]. On the other hand, Sluder et al. have pioneered a groundbreaking approach by developing a novel T cell-targeted vaccine derived from 23 putative antigenic proteins. This innovative vaccine is designed to induce pathogen-specific cell-mediated immunity against Q fever in humans, while mitigating the reactogenicity associated with the current inactivated whole cell vaccine. Notably, the vaccine candidates demonstrated a lack of antigen-specific reactogenicity in a sensitized guinea pig model. Moreover, a specific subset of the vaccine’s epitope peptides elicited robust antigenic recall responses in splenocytes obtained from C57BL/6 mice that had previously been infected with *C. burnetii* [65]. In a different vein, empirical evidence has provided substantial support for the effectiveness of the *C. burnetii* O-specific polysaccharide/tetanus toxoid (OSP-TT) conjugate vaccine in inducing protection against virulent *C. burnetii* infection in guinea pigs. Intriguingly, OSP derived from *C. burnetii* cultured in axenic media has demonstrated comparable potency to OSP obtained from embryonated eggs, eliciting a strong and protective immune response [66]. Several limitations should be acknowledged in our study. Firstly, our approach primarily depends on bioinformatics and in-silico analyses, necessitating rigorous in vitro and in vivo experiments to validate the safety and efficacy of our proposed vaccine candidates. Additionally, while our computational predictions provide valuable insights, they may not comprehensively represent the intricate nature of immune responses. Therefore, empirical studies are indispensable. Lastly, it’s crucial to recognize that our findings are specific to the RSA493/NMI strain of *C. burnetii*, and other strains might possess distinct immunogenic proteins [67]. These limitations underscore the imperative for further research to enhance potential vaccine candidates and account for the diversity among *C. burnetii* strains.

## Conclusions

Our study has shed light on a repertoire of 14 robust immunogenic targets with the aim of combating *C. burnetii*. Among these, seven are notably well-characterized functional proteins. These candidates showcase a promising array of attributes that render them suitable for potential immunization strategies against *C. burnetii*. Their favorable physiochemical profiles, molecular docking interactions, and simulated immune responses collectively endorse their candidacy for the development of targeted subunit vaccine approaches to tackle Q fever. However, the path ahead necessitates meticulous in vitro and in vivo evaluations to firmly establish the safety and the depth of immunoreactivity demonstrated by these identified proteins. While navigating the complex terrain of infectious disease prevention, our findings provide a solid scientific foundation for shaping the future of innovative vaccine approaches in the battle against Q fever.

### Electronic supplementary material

Below is the link to the electronic supplementary material.

Supplementary Material 1

Supplementary Material 2

Supplementary Material 3

Supplementary Material 4

Supplementary Material 5

Supplementary Material 6

## Data Availability

The genomic and proteomic sequences of *C. burnetii* strains RSA493/Nine Mile Phase I (NMI) can be retrieved via accession NC_002971.4 in the NCBI database, which is accessible at https://www.ncbi.nlm.nih.gov/nuccore/NC_002971.4. Additionally, Fasta-formatted sequences for 178 proteins are available in Supplementary Files 1.

## List of Abbreviations

ACC: Auto-cross-covariance analysis
ARV: Automated reverse vaccinology
CCVs: *Coxiella*-containing vacuoles
CDD: Conserved Domain Database
CFS: Chronic fatigue syndrome
CMR: Chloroform-methanol residue
Cpp1: *Coxiella* porin p1
EC: Extracellular
IEDB: Immune Epitope Database
LCV: Large cell variant
LPS: Lipopolysaccharide
mAbs: Monoclonal antibodies
MRV: Manual reverse vaccinology
NMI: Nine Mile phase I
OM: Outer membrane
OMPLA: Outer membrane phospholipase A
OSP-TT: O-specific polysaccharide/tetanus toxoid
PldA: Phospholipase A
RV: Reverse vaccinology
SCV: Small cell variant
tgHLA-DR3: transgenic mice expressing human HLA-DR3
TLRs: Toll-Like Receptors
VFDB: Virulence Factor Database
WCV: Whole-cell vaccine
WGS: Whole genome sequences
