# Supplementary material for: Unveiling promising immunogenic targets in Coxiella burnetii through in silico analysis: paving the way for novel vaccine strategies
Source: BMC Infect Dis. 2023 Dec 21;23:902. doi: 10.1186/s12879-023-08904-7 (PMC10740251; doi:10.1186/s12879-023-08904-7)
Supplement: Supplementary file 1 — Supplementary Material 1 [file 12879_2023_8904_MOESM1_ESM.docx]

**Supplementary File 1. All protein sequences (Fasta format) that were identified in this research.**

>NP_820793.1 outer membrane protein [Coxiella burnetii RSA 493]

MGRCILYLFCLLSALFLIPTNAAAGGIEVPHYIPIPFVYSGVYVEGLYGYASRDWESYLPYQTYVAALQG

DGPPFGPFVKRGKGGSVAGVDVGYQWNRYFAAEIGWIHLPTVRYLVPSGLPVAGGRISEHSWLGYIALKL

AFPICGNLYAYSKVGAAGLNIRTRINFTPVNDLTQNGDFWSPLFAVGLQYYLNWYWFFTLQYLHVNGYGR

ASAGAIQKLPSPHSDLFTFSVGYKLAI

>NP_820237.1 long-chain fatty acid transport protein [Coxiella burnetii RSA 493]

MVFSFTLITSAIFASGGQSFYPFAFINPAELSQVKHWKVSVGYYAAFVRNKFQGSVTIPTGLSFPTFQTV

TQTFSGIARSSDVSHLPGGQIAYRINDRFVVGLRYSEPYDSTISFADTFGRFANNKSSTRVNDISPEFAF

SFSPKFSIGAGLDIMHLFAEANINTIPFLIPFSPLSVNEGLSKNHASGWGLGWHIGMLINPWLGNYLGLT

YYSRSRYTLHGVTQFNGSFLDGTPIFIKNGIVVPQARPDTFIFSDFQALSEKFGLFLHLTYTLWSILQSI

TVENVAVPNPPYDSNTFTIDFKYRNAWRIGFGGRYAPSENWIFFLGTTFDQTPVPSPQYRGLFFPETNGI

HLWASLTRKFTKHFSTSLMYSHSFYQKRAVDNSFAGVTSLGVNRPVVDFIGLDLTYES

>NP_819100.1 outer membrane protein [Coxiella burnetii RSA 493]

MNPLYRQKILISALAILTLSFTACARKPAPPPPPFSVLRVTTPLTHARQKALAATLQSRGVQMIQQGSRL

QLILPVNRFFLPQTTTVKEKHIETLRLIALYLHNYKRTHIVHYPIKVYAFSGTVHSRRYRDYESNQYAQV

IGSFLWNHGFSPAQLSVVGFGAKHPIASYRTPEGNAFNRRVMIQVN

>NP_820680.1 hypothetical protein CBU_1699 [Coxiella burnetii RSA 493]

MKFLIAVSLLVAALFISFPGDARSRKTPTEDEIRRTLQTVTRQEKALRREVNDLQVELNKVKTRKHAEQK

KSTANTLARPGALEEDIDLDHAPYGYFGALRFRHGITITTSPLLGLKSAFNASDLLYEYPSMNEDLLLLR

QRQYFELHLLEVGDTLDNRAIVVLSGALEGQIIHRRDFDHRTRGDVNLTTAELDIMPMFSTWANGLIVLD

YDDSPPATGSRVTNARIYLSRGFLTIGNLAVSPLYFTLGQMYVPFGKYGSAMLTTPLTKSLARIEARAAV

LGYYHQGFYTSVYGFQGSKTSGSDHVFRQGGLNSGYQNGGFDIGAGYVTNIADSEGMQDNGIPPFVTTFL

PENIRRDVLTQFGGFGETPSGNNLQHSVPALNGHMEFSHGPLGVLLEYITAVRSFAPQDVTFNGAGAFIR

VMHAEIDFTTHYHNKPILFGFAYGQTWQALAFNLPKNSYSFVISTSIWKNTMAGIEFRHDVNYGRTALAS

TTGSSLPVPPANIGGHRNMITLQFGAYF

>NP_820398.1 hypothetical protein CBU_1414 [Coxiella burnetii RSA 493]

MKMKRSLRSLASSIFIFFLSLITINAFAGGPEIPSFNPWTISLGIFGAIYGAHSKHESIVGIGPVDMPIT

AIRNYTHYTKQFGYGGGGQVGIRYHLERSYLGLELSAQGNSRQGNNDSVTFVGSGFLFLLQNEFRINSNV

DLTGILGTDLTSRTHMYGKLGVSYAKLNQKLTVSQWMASSLQTSVQQKLNKNLWGYVAGLGIAEDLGRGI

SLFAEYDYYDYGKNDLKTLRNIFPSSVLVDIYTQKVLVGAYTIRVGFNIEFAV

>NP_819949.1 OmpA-like transmembrane domain protein [Coxiella burnetii RSA 493]

MKIVLALIATVAVGLGTESLAQGAAVEEESGLYTNGDLVGVKPKVTNLGSIDDGGIIYKAGGGYQFNKYF

ALEGNYTRFPNLKSGRVIPEKSNTEIYAAGKAIIPLKKGYNVFAKIGAAQVSITTVPGMEDVSGINLSNT

HSEILPYGGLGVGYSVSQKVGFNLQFAGTPEGDNIPARYAVMGGLSYNF

>NP_820396.2 hypothetical protein CBU_1412 [Coxiella burnetii RSA 493]

MIKGERFMGRRRKVWSSVALGILWGAFSLIGSGSALAGGYEPLPESPWMVYFGVFGGYHHATYQYEALYH

NDGLPRNQLFKSEIWQYGALWGGHVGIQYHFIRPYYLGLNFAGMSNSRKAQFNGSVSLGAGSTTVPVIDT

TLRLKYNFDLTGIFGIDITPALHLYLKGGGSYAKLVEDFAVLQATTSASISQQHQTHHGLWGVVLGAGLA

RDLCRWVSLFAEYDFYDYGNHTLNTANNISVDPVVAPPDVYTQEAKNIRAYTIRAGINVNFNL

>NP_820960.1 hypothetical protein CBU_1985 [Coxiella burnetii RSA 493]

MLFEIKIYMYRKIISQFISLLFLLLINFASKANSIQTYDFPSIWRTHPFYIGLSLGYGSTDWSQLVAKGT

PMEIPLLAVSTPISAGDRGFLYGFVAGYELQPHFAIELNYTRFPNTTVVFDPDFYTIKPIVSMRSFTYAY

NLVGKFMAQLGESGFRGFANAGASFTHRNDLLVKGAHICPTFGVGINYVFIKRIMLEMGFQYYAGYGKAV

INPGVNYVPFLYALTLKLAYRF

>NP_820397.1 membrane-spanning protein [Coxiella burnetii RSA 493]

MNIKRRLKYLTSCLLVSAFFWINSSAWAGGPEIPPSAPWVIYLGGFGGIYVANFEYQGTYLGGSFTVPIG

SNVHQNGYTAGGHIGLRYYFSNPWFLGLEFAAMGNSENATTAESVLAPSPDDLIFNLVNQFRIKSNLDLT

AQLGVNITPQTRVYIKGGASYARIRHILTVFNPATLTPTISLQRTTHKNRWGFLVGFGLGYDFCPWFGIF

TEYNYYDYGRVGLDSLSNIRPNNGADTYHQNVRVHAYSVLLGVNLNFSV

>NP_820235.1 O-sialoglycoprotein endopeptidase [Coxiella burnetii RSA 493]

MKCVLGVETSCDETAVALYDGERGLLAHRVYSQIAIHAEYGGVVPELASRDHIRKILPLIKAALDDAALS

KENIDGIAYTKGPGLIGALMVGASVAKSLAYAWRVPVVGVHHMEAHLMALQLEESRPAYPFIALLVSGGH

TMLVHVEQPGRYKILGESVDDAAGEAFDKTAKLLGLPYPGGPALARLAEQGEPKRFIFPRPMVNQPHLNF

SFSGLKTHAVNCFKQYGGEEQTRADIACAFENAVVDTLIIKCLRALEKTGINTLVLVGGVAANKKLRERL

GQVAVKRAAQIYYPRQEFCTDNGAMVAYTGWLRLNAGEKEDKIIRVKPRWSMAELNIIN

>NP_820135.1 enhanced entry protein EnhC [Coxiella burnetii RSA 493]

MKRIAVFILTLSFFSISYSDKNPVFQEYYEGNYRAAETGLKQLAEKNNGEATFYLATMYMNGFGVRRDFE

KGFDYMTRAAELKYLPAQLYLGNYYFQQQKDLEKAVPWFKKAADAGDAGAQLFTGISYLNGYGVKKNIDI

ARKYFIRAAQNEIPMGQYELAKIFLASRHAGDRRMGRIWLTKAADKYNYPDAQYLLGTMLYTGNEAEKDP

VKGVEWLEKAAANGSKEASKTLDKINRINTSDAKANSENRSEPTPWQIMVGLMQKAGVQLNNPITVTASI

NNFTKTPKSMALDKNSIIKLNLNLVNSKDIPPEKILSYMTQLNYKEEKFDLTVPAYPFEMPPGANNYKEA

FQSLSRVANYGYAQSLFRLGQMYENGLGVQKDPETAFQLYMKAAEQNYLKAQYAIGTYYLQGKGVPQDYE

KAISWFIRAALKGSLQAQFVLGNIYERGIKASNNKILFKNFDRAKAMYSLAVGGNLPIAAYRLAELYVSG

FLNPDNNVSLETQNWKKAYALYQKAAKSGLEKADVALGYFYLQQNQTTLAEKTFEIAQKAYQTNDPEAAM

LLAILYDRGFGVNRNSRKSAEILEKLSKQNNAIAQFMLGNYYLKNKRKENIAISLLEKSANQGNGYAKYN

LAILAKQNKYTKPGENFLSLLIRAANHYDKIKEILADYYLLDTPVPGSEKKAVAIYQELANKQDPAAELK

LGFMNEHGLLFPKDYHKAEEWYQKSAEQGNPIAQYLLGNMYYLGRGVDRDVNKAIDWLKKSAAQNYVPAK

VGLGFIYEMSKHNYPEAKKWYTLASKFHNPQALYNLGLMYEYGKGVKSDPQKAFRLYKDAAQNGLDLAAV

QVAGMYLKGTGIGFDPNTALKMYSQAAQKNNSFATYQLGLMSESGVAQKIDLNKARLYYEKAAKEGSVEA

QLALARFYEFGISVPADISKSINFYQAAAAEGNEFAKQQLTRLSNQGKSSSNAMPFQCVNQVALEKVKNS

FWKKVTDWIAPVPNIDYMNAIDYLNSGKVEQATTALQKIIKVRPNFQPARETVSHYFCQKADRK

>NP_819970.1 short chain dehydrogenase [Coxiella burnetii RSA 493]

MKRILITGANRGIGLELVKQYLAAGWHVDGCYRDKKASNSLFELAAEKKQSLTLHELDVLDEKAIQALGE

HLKNQPIDILFNNAGVSAKNLREFGSIHDTENACEVFKINTIAPLLMVQALLESVEKSEKKLIINMSSEM

GSIAQNVNGNYYVYRASKSALNAITKSLAIDLKRRGITVISMNPGWVRTDMGGEQAPLDVISSVRGMREV

IERVDIKSTGGFLGYDGGEMPW

>NP_820252.1 nucleoside diphosphate kinase [Coxiella burnetii RSA 493]

MAIERTLSIIKPDAVAKNVIGQIYSRFEKAGLKIIAAKMCHLSKPQAEKFYAVHKDRPFYPDLVKFMTQG

PVMIQVLEGENAIVKNREIMGATNPKEALPGTIRADFADSIDANAVHGSDGPETAKEEIAFFFKPDESFN

SIGV

>NP_819381.1 membrane-associated protein [Coxiella burnetii RSA 493]

MRKSHPTQQSLFKTNPQFLAQIKRRQQNNETKDTNSPLSISASLSKHVLAMLPFTSLTTNVIASEIKNSI

GKLCQSTIHLPFNGTTICVKPEIDSLNAPKQYWAMAVENISGVFNRSGFNVTSREILNCLQGEILASSPY

SSSCDDNKKTHQYWNLFAFPSQQSIISNISSFLNKTIAPAIQEILNNITNLIQNDTEKSCDHTNVFGIFL

VAAGAFIVVMGTAIWLDEQSKKNQIRSEQSLEERNPDYQFFQA

>NP_820267.1 hypothetical protein CBU_1274 [Coxiella burnetii RSA 493]

MVRNPFWDIVRNGREKKTMRNILLSLFLLFSLTAALAAPETEFTDPNKPVVVSADSPHIVLKIKSNPTTG

YSWFLVDYDYQLLSPESHQYIRPSGNLVGAPGFEIWQFAVNADAFRVPQVTKISLQSIRPWTVPVNGRKL

KFVVIIHSEKTPALEKKEGQ

>NP_820283.1 hypothetical protein CBU_1291 [Coxiella burnetii RSA 493]

MLKKIYAPLAGTVTDMQVTRYDRIRRSQTLLQLKAGETLTPIVASYDGWVRHVFIKSEQAVTTGQVLFII

DVVDINDFQPDSTEVNPHTELGEAGRRGLERQGQRSFGHPAGPLFDAPEASNGMGHRGSVKPHPHLIGMK

EGTPNKMSANAATNKTAIEQTADNATHDPELAKQLSQQLQQRLQIGSALHKSPTLSR

>NP_821052.2 hypothetical protein CBU_2082 [Coxiella burnetii RSA 493]

MKQTLLRYFIFFFFTLIVESACANPSIQWKALNPGMAYTVVTPAFSSESRPGLFTHLYAWKINPRQYHFN

IVTAKSLQQTALYAAQAAKIKDTVLAINGGFFTPNLEPLGLRISDNKVLSSLKRISWWGIFMIKNNRAAI

TSPQNYRYSPEINFAIQAGPRLIIDGRIPQLRGGSAQRSALGVTPTGDIIIAITDNNLLLTATQLAILLQ

KLGCSNALNLDGGTSSQLFVHTNNFSLQIPSLRPVADLILVKIPS

>NP_821049.1 hypothetical protein CBU_2079 [Coxiella burnetii RSA 493]

MSYIKRDHTALRDIAMKTFLKVVGLAASLSAASVAFSSYQLIIKNNYNQTVAISLFDDQGGTHDAGEVEA

NGQTKITAHLDQASGFCLNVAGKREVVCSYKSGSHPNGTITIDSTGRYCIYNNTKKISGGHGCG

>NP_819254.1 hypothetical protein CBU_0209 [Coxiella burnetii RSA 493]

MRHTFPEGRAERMLGRIRGVLKDPRSQTIAPFTKIDMIIALVKMEMFPKAFPRISNGIAEIFSFLLERLE

DFFQQTSLSESTLADFLDAVSYLNLRHLTPRSTSTLVSRLQTVIERALYLKMMVDYDIDPNEINEWRRRY

QQISSQREGVIQDIAINKIAYDATREKIKSSAYLIEAAIKAGMRPQDALSLYTPYNIYQTFGERESITDI

VWGIITEKTWGFQPVEEISGIYSLYLALRQAFPTDTSVLEQWHDLSSKMLRGMPLEEFERTYTRILYPPS

PPQLVIPATTFFQEPVWGSQRGQAIDFLNQTFSEIKASSTWDYTSV

>NP_819315.1 single-strand DNA binding protein [Coxiella burnetii RSA 493]

MARGVNKVILIGNLGQDPEVRYTPNGNAVANVTLATSTTWRDKQTGELQERTEWHRIAFFNRLAEIVGEY

LRKGSKIYIEGSLRTRKWQDKNGVDRYTTEIIANEMHMLDNRGGGNSGNYGNHSEGGASNKQSAPTSSQT

PTAGDDSSVADFDDDIPF

>NP_819762.1 hypothetical protein CBU_0736 [Coxiella burnetii RSA 493]

MRRKSLLNIFSIIFLSTLTSLAWADMTVTKRVDRYGFLQGVLLQSKDVSFRVNTYGYLESVTPHDPKHET

LIRLDKIIGKEQVPPSAQKLSIKYYPASALNGNGGKIAEANGIRVSYYPCAPATRFSNAHYYGQKFSANN

NSLPFVINPRYRGACSSTSGNGGKVRRIGDINFSYYPSSTFSSSGGKLQRVGDTVITYFPQNSFQGKGGQ

LKNFNAK

>NP_819410.1 membrane-associated protein [Coxiella burnetii RSA 493]

MTTLFKNVTLLTALSLSLVFGLTGCDRRTVGATTGAAVGGITGAALGGKTGAVVGGVAGAAVGSAVTSRR

YRHYH

>NP_819757.2 hypothetical protein CBU_0731 [Coxiella burnetii RSA 493]

MAIVYNCFIFIHMKRKERMKRYSSVCLAVLGGFVAIGLSTAILAANCPKNMQKDAKGYWTSNEPPGWKSY

RPSESDSTLEPKDFGGAVYSPAKKRIACAYKTTNNKWAILLSSVYYPFEASDLKSTTWKYNPQHKDYICG

SPKQTLDTCQFEIKNN

>NP_820182.1 hypothetical protein CBU_1187 [Coxiella burnetii RSA 493]

MGGSLIALITSIFLATSLASPSAKAFDYSDFQPLINKGLSPQALQVGLKAYRWARTHGAVKKPIMTLIDF

KQPSNKKRLWVIDMRNGKLLFNGYVAQGKGSGNLYATRFSNKGGSDASSIGAMVTGESYYGHHGLSVRIH

GLEKGVNNNVFKRAVVFHSAWYATKSFAEKVGRLGRSWGCFAIDPKYSKYVFSKIKGGSFVFAYAPQEKN

DPNFS

>NP_820633.1 membrane-associated protein [Coxiella burnetii RSA 493]

MNKYLLIGTAALLSAASSFCFADNTPTRDCANYLSYSDPQFINLVSHNQSLASTIADALNNCALASACSS

ISVDHCSALLSTRSFISAYFANFKGDSVSSAVPHGGGFSAAPKLPTTALPIVNPPANTPSPVADQPSSTQ

TKKQDSSNIHWF

>NP_819437.1 membrane-spanning protein [Coxiella burnetii RSA 493]

MNSIGLGTDTLIQIGVFGKIFTTMSFKDENPLLQKTMKTKYTLFFIILGLLTVNEISWAKPRANFAPQTI

SCSYSSQQLVCSGFDQQLLTASLEEGPPPQKEKTIYHFVKATAEDPSSPLLVYHYQDASQQSSISLIPLY

DGVKPFARFRTWVYHSPPLYGAYYYTCTTGVAVECPYTNTPF

>NP_820832.2 type 4 pili biogenesis protein [Coxiella burnetii RSA 493]

MFMPWLRFLIIFLMGFAISACEKSYSPLYSDANVYLTIAYYEEQKENTDLANSYYQRAVDSAPNSDRVHN

NYGVFLCRQKRYKAAIEQFVVASKINYKYRVMAYQNMSLCKKLLH

>NP_820878.1 peptidase M16 family non-proteolytic protein [Coxiella burnetii RSA 493]

MRLLIVTISLFISLNVFASESTPLVNIQHWETKNGAKVYFVRASEIPMVDIQVVFAAGSSYDGQAWGLAS

FTNSMLAEGTTTQNANQIAMAFDRVGAQYSNGVDRDMAMLSLRSLTRPDFLKPALKTFADVLTESTFPQK

AFIRVKHQLLSSIEYNEQSPNVVASKAFYSAIYGTHPYGHPPAGTIKTINAITNDEVKSFYQKFYVANNA

NVVIVGDLTREQAQGIAAQVIGALPTGKPAPVLPEAITASGVLRQQIPFLAQQTTIILGQVAIKPASADY

FPLVVGNQVLGGLPLSSLLFDQVRNQRGLTYGAYSQLAPLKYGGPFYISLQTRKDKAADALKITQSVLQH

FVEKGPTLLQLQAAKNNIIGNFPLQLSTNASVLANVTNMVFYGLPLDYLDTYRQNIRAVDTQQVTAAFQK

TIRPAQLKIVEVGSSTPLKGGEE

>NP_819822.2 hypothetical protein CBU_0802 [Coxiella burnetii RSA 493]

MKGKPMPPQLSPRSRPAVVKKELEKNIRTELQHYSKLNIDQRQQWRSESALLLRLTRNNNLIWALSQMAG

NDPVKMQQLYLKYKSLIQANLSKQQQIPQRMQNLAVDQGPNLPANMQLNPNLQNPEFDPMALAVVNLQTA

NILNELWQSKTNDLQSAEINEEELTTENQMHDNESHFEEVWSGIIASQKLHEAASSHENKHEPKPDNIPL

EATKLEEMKKSTLSSILEGGLTPKLAASALAIDEASKKLLEEASLGHHHKSSPMPSPG

>NP_819191.3 cell division protein [Coxiella burnetii RSA 493]

MALGDNNMFELGETSPQNAQIKVIGIGGGGGNAIEHMIAENIDGVEFVCANTDSQALGRSNARVVLQLGD

EITKGLGAGADPSVGRQAAEEARDRIREILEGTDMVFLTAGMGGGTGTGAAPIFAEVAKELGILTVAVVT

KPFVFEGKKRMDVAEEGIKALGNYVDSLITIPNNKLLNVLGKNITLLNAFKAANNVLLGAVQGIADLITR

PGLINVDFADVRTVMSEMGMAMMGTGVSSGENRAREAAEAAIASPLLEDVDFTGARGVLVNITAGMDLSI

GEFEQVGEAVKAFASETATVVIGTVIDPDMSDELRVTVVVTGLGSHAGGGAGVPLKPVKNTKNDGTLDYH

QLDRPTYMRNQEPSKRTVDLEEQRDRDFEYLDIPAFLRRQEED

>NP_819099.2 hypothetical protein CBU_0044 [Coxiella burnetii RSA 493]

MMSRLRWFALTLPFLFVSLAYAGGPEYPPTKNVSVSCVNAEGYFNLILKKGSRIKVTVTSHHLEPLRARI

IHHTLYVRAPWLPPIQKRPVVTVTMPTLTRLVVNGPTNVRADRLQSSGLSILSLGWGNIQLKGKMKVNHI

VQRGQNYISLGWLDSSTLSIDSRGNGSLYLAGVAKMLYARLAGNAILGAQYLRANHIQVQTQNNASAYVM

PLNTLRAFASGRANIYYYSYPKNLTRDTKQSGNVLQMAWRE

>NP_820817.1 dTDP-4-dehydrorhamnose 3,5-epimerase [Coxiella burnetii RSA 493]

MPFEFQKMLIPEVILIKPKVFTDDRGFFIETFKQSDFRRHGINGEFLQDNHSLSMKKGVLRGLHYQLDPH

AQGKLVRVVLGKVFDVAVDLRRESPTFGKWVSTELSSTNNHMLWIPPGFAHGMLVLEENTHLLYKCTAEY

VPESERYIRWDDPDINIKWPIKNNLLLSEKDAAGVFLQRAEINAQYHGS

>NP_819725.1 hypothetical protein CBU_0695 [Coxiella burnetii RSA 493]

MRKLRKKYLIKIAARIRIFFYSGAKYLLGIYALDKVPQIVKENSLASGITFLKSKYKFFRPPVVKFFIKL

YRYLLITDKNLLTLQTDNTYIIHFCCWGKAYAEKVKTYLIPSLLAEGNLPSIAKKYNTTVLIHCDIDTKN

NLSASPIIRHVKNYANIEFIILPRSLIKAYKSNFKYFPFLSSKKITGIISNIKYFLLGALQTQALEIAVK

NKSYVSFMMPDFVLSARFLTNIFFLIEGKKVAIISTFRTDYQKIAQQLEQFFKGTERIQLTVPASVLTTF

QVTNLHPAAKKIIVSENNADFTSRAQLLFETPNGFVLRAYHYHPILLDCHHYNYKFKKDYYPIDNSVLNQ

LLSINIPFDQQIAACNNASDIHCLELSDEHIDQHISTKPKKLNYNELLYAICDMISKNPSTYDTPLNRYF

ISIKNRFESPAILKEGDFVDDDRFFSDLKEVLNTYYYN

>NP_820808.1 outer-membrane lipoprotein [Coxiella burnetii RSA 493]

MSLISNNEERSLRVRYCIAIALSALLISGCTTLRLPNQSTSVYHQQTWAQRYYDLSRISQWNIDGAFSIQ

QPGKTIIAAYDWQQKGMNYRIRIHSSLDIYSVNISGRPGMVTLWRSPRQHYTASTPEQLMQQQLGWQLPL

SNLYYWIRGIPAPGAYQADFDTYTHLIALQQSGWHIRFSQYTTVGSVDLPRTLQLSNGSLAVKIVVKHWQ

>NP_819521.1 ribonuclease E [Coxiella burnetii RSA 493]

MKRMLINATQPDEVRVAITDDSKLIDLDIEIPGQEQKKSNIYKGIITSIEPSLGAVFVNYGSERHGFLPL

KEISREYFLQDIEGDFDSIDINRVLKLGQELVVQVDKEERGTKGAALTTFISLAGSFLVLMPNNPRAGGI

SRRIEGNERDQLRETINQLSLPEGMGLIVRTAGLGRPKEELEWDLKILLRYWEAVKQAAVAKPGPYLIHQ

ESDVIIRAIRDYLRQDVEEILIDDEVAFENARHYINQVRPQFVERLKLYREHLPLFSRFQIEQQIENAHQ

REIRLPSGGSLIIDHSEALIAIDINSARATRGASIEETALSTNLEAAEEIARQLRIRDIGGLIVIDFIDM

TPLRNQREVENCLRNALSQDRARIQIGRISRFGLLEMSRQRLRSSLTRSTQIACPRCNAEGTIRSIESLG

LSIIHIIQEQASKAKNIHFQLQAPVDLATYLINEKRELLRGIEHHYPVKITIVPNQYMETPLYQLRQIKI

DPASLEHGKGMASYKLTKVFKTEAPQKQEIRALSEPAIQQFLTSPPAPTTPRKTPTPGLFKRLMGKMFGS

EEEPSTAVTPPVKPHVPERHKPSSPPRGRRQGGYASRKSTGPRRHHPRSGGGGGGHGQNRHGARSRRGTR

GGRRDHGGGRGGPGYHTQSTGPMPPLGEDVVDVNQLPMSTPPSPPPPFLTDADVPPSRDERRSGGINGDA

DHGAKPHPNGKVSDDNRGNR

>NP_820226.2 membrane-associated protein [Coxiella burnetii RSA 493]

MSRQSFSITNFFSPNNPNSLKHKNPKALEAIKRNLEKKGVLPSAGKKYSSPQLLDQAVQGIVQVLTFSAL

PGLANAGKWATNNIDKLKRVVCGLSLSDAYPGSSICVQPLFSKCFQSWSTLPDDFESKMRAVLEQFGFTI

EVIPSSIIKTCQNVTKKFCEYYDECDFETVIGACPENKTEAEPLINFVKNISASFDGAFQDACSEMHMNA

HDVVTSVNIGLEILGGLLGFFF

>NP_819159.1 methionine-binding protein [Coxiella burnetii RSA 493]

MLKKLMRLLISGVMLVGLTACHQKEAKNEVRVGTIAGPETQLMEVAKQVALNRYGLHVNIITFSDYNTPN

EALADGSVDANMFQHLPYLKAQIEMRGYKIVSIGKTFVYPMGLYSKKITALTQLKTGAKIAVPSDPSNEA

RALLLLEKAQLIQLKTHVTINATPMDIASNPKKLKIVELDAAQLSRSLGDVDLAAINTNYAIPAGLSPSR

DALLTEGPNSPYANVVAVREDDKNDPRLKQLVSALHSPAVLSAAKKIFGDGAIPAWK

>NP_820955.2 peptidyl-prolyl cis-trans isomerase [Coxiella burnetii RSA 493]

MWKKILTSMVIILSVTSISAFAQSTLPAPNATHEQSLDQIVAVVNDEIITQSELNHALTAAKQQFMQRQI

SLPDQKTFKKQVLDQLIYQKLQLQVAKHNQIKVTNNEINAAVARISQANHLSQTALKQKLTQEGISYKEF

RSQLQKQLIISKLQHQALQDTISINKSDIAAFQKQHAGQIASKEYHIATILIPLPASATQAQINHAKGKA

ALVLKQLQKGSSFETAMKMHPGSADLGWRSAKELPQVFVKTVLKMKPNEVTGPIQAPNGFHIIKLLDKEA

KNTVSDQQIQRIVYQQKVEKALQKWLTQLRSSAYIHIYADS

>NP_820941.1 glutamyl-tRNA reductase [Coxiella burnetii RSA 493]

MPLLVCGINHQSAPLTVREKLVFTPERTPLALQSLLAEKAVNEALLLSTCNRTEIYTTVDEAATILRWLS

KQPQLSGIDLRSFCYARRDIEMVRHVMRVGSGLDSMVLGEPQILGQMKQAYLLARRIGAVGSELGRLFPA

VFAATKRIRSETAIGANPVSIAYAVVQLAKRIFSHLNQCQILLIGAGETIELVFSHLYNQGARHFFIANR

TLTRAKQIAEPYHAQAIRLSDIPTYLPKVDIVISATMSQLPLVGKGAVESALRQRKRRPLFMADLALPRD

IEPETAQLEDVYLYNIDDLQTLIAQNRQTREAAAKQAEAMVEMQAIHYMRQLQVHKAGDTIRRFRERVEM

LRDQELEKALAHFQRTNDPKAVIAHFAHNLTNKILHQPTTKLRQAAYEDQVQLLLSAKELFDL

>NP_819085.1 3-hydroxy-3-methylglutaryl-coenzyme A reductase [Coxiella burnetii RSA 493]

MNKWEKIPYGFDESALKRRRQWMKEQGLNWESCSPELSGSMRGLIENHIGQIAIPLALAGPCVIDGTYAQ

GTYYMPVATLEGTLTLSMSRGFYATAQCGGIQTQHIQQMISRSPIFSFKDLNSLKVFSTWVTEHFNTIKN

VAEQSTQYGKLIRIDQYQVHDHLILDFVYTTAEAAGQNMTTFCTHNACEFIRGTLAEHLSFTYLLESSFN

ADKKPSTKTLLAGRGHKVIASCVLSAAVCKRTLGISPRQLAEGMRVARYASTLANIVGINLHVANALTAI

YLATGQDCACVSENAVAFFDILYLPESDEAKCVLTMPSITVGTVGSGMQLPQQQSNLKLINCVGKHSSKR

LAELITASALALEISLAAAIGANKFAKAHMIFGRKKKKRKVHEKI

>YP_002333032.1 cupin superfamily protein [Coxiella burnetii RSA 493]

MIDEIMGGKKYEHIHAYPITIRPLSKEDGGGPLNLYLIVSDGTFNKVKLGPKACNLAMDDKLIKRFSKTD

RSRIIFAAGFHKKVARMQRSGIRELRFLSPVFSLNFRFLVSDGGVYFQIVNFHQRFRVFGNSKSFFFVNF

SRFFRCSKRPRF

>NP_820794.1 primosomal protein N' [Coxiella burnetii RSA 493]

MNPPPFILRVAVPSPLWQSFDYLPLNAEIQSLQPGMRLKVPFGRRETVGILLDVVTHSTLPLSKLKPIIE

IIDEVALIPASLLKLYCWASDYYHYPIGEIILGSLPRLFRQGKTIISEKIEELNQAAPPSLELNEYQQNA

VNQIMASKGFQTFLLAGVTGSGKTEVYLRCIEKLIQQEKQALILVPEIGLTPQTLNRFRERFNVPTAVLH

SSLTDKKRAQAWMMAKRGTAKIVIGTRSAIFTPLLNPGIIILDEEHDTSFKQQSGFRYSARDLAVMRGQF

ENIPVVLGSATPSLESLYNVERRRYQLLSLPGRAGKAKLPSLTIVDLRQKKLIAGMSEDLLISIEKHIKN

KGQVLLFLNRRGYAPTLLCHGCGWVMHCDRCDARLTLHYFPKRLYCHHCGAAKKIPPTCPQCHQQELIDV

GLGTERLEAALQQRFPNDDIVRIDRDSTRTKNSMENKLNLIHNREAPILIGTQMIAKGHHFSHLTLVAII

DADSGLYSADFRATERMGQLLTQVAGRAGRVDRQGEVLIQTHQPNNPFLTLLLQEGYEAFAQALLKERQL

VQLPPFTYLALLRTEAVKQNLPLDFLTKIKELMVNELKEEVDLLGPVSAGMERKAGRYRYQLLFQSKHRK

HLHSVLNKLISLLATQRTKVRWSLDIDPQETI

>NP_820854.1 hypothetical ATPase [Coxiella burnetii RSA 493]

MKDELINLIHQSNPWFKNTTLPVFKDGYIARLQTEKLLLPEWDNLWLILIGPRQAGKTTLAKYLSQALIK

GQRFNNLLYLNCDLLEIRRWLTNPLFIKEAMDELHLERPIILIDEVQRLETPGLLLKACADLKLDIKMIA

TGSSQLEMKSKVQEYLTGRHLEALVLPLSYQEIGEVDESQLIYGCYPAIVKASEKSILLRQIYQDYIKKD

IIEILKVSKPDTMQKLITLIAHSSGQLVNYNQLATDCQVSISTIQNYLSILENTYTIARITPFVGNKRKE

ITSNPIFYFIDNGFRNQSLRNLSTSLNGRQDIGLLIQSAVFQELLKFKEQYFYDFVIQFWRTQNGAEVDF

VLYKNENCIIPVEVKFKTMKKPIISRSFRSFTEAYKPKVGFFITKNFNKKIELNNCEIYFISFSRLFNFF

EILRTRL

>NP_819961.1 hypothetical protein CBU_0952 [Coxiella burnetii RSA 493]

MKKLTVTFLTFISIFFAATAAFAENRPILNTINYQQQVEKWVTTDSADVMVSVNVTTKEKKFDALQHQVM

KKLEELSDGRQWHIDSFSMSQDQSGLEVLSWEVRSRMPLALVNSLRQKIDSLSQAGQQYKIQNVDFEPSL

VEKEKAFAELRQRVYDQVKVELDNLNKSFPNGHYFLHSIDFVSPPLYAANQKELTLMRSAPSEKTAVTLG

RNLMLIANVKVATFLNK

>NP_819978.1 membrane-spanning protein [Coxiella burnetii RSA 493]

MNKKISLFLSLSIALTSGLTFYSAAAEETLDVQFLILAIVVLFSEFFEIKDHLEHTHDHHDAARKPLLPL

PGIDPPNNYSSTNQSQTTNVLLNRTSTISTIVKGSATICTLPFFGFACYGLWCQSKSLMDFFNISTKLTF

IPAIFLWGRYALIPVSHTIRNIWSQEGVEKFTARVTHTAERKGFIQNKAVLFFTFLGLSGTHLPESFLLS

NKTKGWKEILSILGLSIPRAIAHLDHVRALPQTLKEVWQKNRKGKLALLCFAACGAGLIHASPALLSTFY

YWDKTDIELKIALGTTVGIETLTGAGEFLQHGGQHALEFFTSKTIGNPNVNTENEDVEAAAPSVSGTIGY

T

>NP_820789.1 macrolide-specific efflux protein [Coxiella burnetii RSA 493]

MRKRMFQWGVCLMLAAAVFFTGCKRDEKKEPEKDRQIIVAKLQPSVTQLYFKGSLAPLRTVPVLSPVDGT

VTKLFFTYGASVEENQNIVEIHSQKLAEDYRDAVTKYLQAKDTYETSLKSFAGTRALYEAGIISEEEFRT

ERSQHDTNVLNFYQARFTLEKVLREADIDPKTVEGLRIADINAVAQILQKHFRHILVKSPGKGVALFPVK

SEDDKDGKGQLVVGTEVKQGQLILSIGDLSGFTITLQVSEININRLHSGLKATVTGDAFPGITLHGIVTA

VARQSNPEQSEGDGALSTFNIEVQVPGVTKEQRNVIHVGMSATVEIDVENPSHIILPIGAVTPKNGQSVV

TIIDPKTGKETKVPVVTGDTTLTGVIILQGINSGDKVVIHD

>NP_819563.1 orotidine 5'-phosphate decarboxylase [Coxiella burnetii RSA 493]

MEKPDPKVIVAIDAGTVEQARAQINPLTPELCHLKIGSILFTRYGPAFVEELMQKGYRIFLDLKFYDIPQ

TVAGACRAVAELGVWMMNIHISGGRTMMETVVNALQSITLKEKPLLIGVTILTSLDGSDLKTLGIQEKVP

DIVCRMATLAKSAGLDGVVCSAQEAALLRKQFDRNFLLVTPGIRLETDEKGDQKRVMTPRAAIQAGSDYL

VIGRPITQSTDPLKALEAIDKDIKTR

>NP_819929.1 membrane-spanning protein [Coxiella burnetii RSA 493]

MRNDPSQALLPRAPVANKFFKFVIIQYSALTYNAAVLATYWISADRFPEHMKWLENSISQEKGSLKLVPV

GVFIGMESILFLTNQRTMPKRIEALLRQEESCIFSCYSLPERYKTIEAGISAFWKAAVSSTSLWALFTDL

ISLTTLNRSWSIGLGGALTFSLLPTNFMAQIANFLKNYLQDWRLPAWFAWYCGIGYGLCNAALYFNTFNK

VLEPWVGGSLTELDNLFKKILFSLNTLISFKFAYSTSKIYSKRIQKMITPLIEESTPSHPHLTFSGTIDA

VWKTVVTLLSVIRIVNDYHSLTTWEFVVLIFTLAPGVLPQLTFYANEEEAQRPNLWSSFCSFFSCSQDTE

LSAVVSDEAPTPFEQGGSLTI

>NP_820715.2 hypothetical protein CBU_1735 [Coxiella burnetii RSA 493]

MTGGFAMMAVRKISILVITLTCFFLTNAGMAVEIRGINVATVAVSDQSPQTLQTALPQALEQVLIRMSGN

TGVMTLPTVQDAVPQISHYVEKYSYATKTDESGKQQLLLQVVFDTRAVKRLLQNANQAIWSANRPLTMVW

VSIPNGKQSQILASDSQNPALHALKQVAAFRGVPVIFPAMDLEDQINVAQPASTLPNNQQLQAISQRYGV

DSILSGAVVSGENGQLQGEWQLFLNGTPYEWQTSGANVVQVVNNGIDRAADMMANQFATLDSKGMGSLVT

MQVTGVKTLDDYVHVVSTLKHLTPVAKVSVSDMNADMLLLKIKTIGNLDDLVKSLKSVSHLVAETAPTQP

ELNAANLFYRWKSNTPPGDSETPIMLTPPIH

>NP_820094.2 hypothetical protein CBU_1095 [Coxiella burnetii RSA 493]

MAATQKQTILIVKAIIIEETNMNIRSLALCFIAMLGLGISGATFAQGKGISPELEGTLSGFYVKNNTRED

YNLVLFGTHCIHWVKVNGQELKTAVALPKNSEASIELHIDQEGGSCAYEGSAIIIVSKTTKVFNDAVATL

NLSGKLEPGTSSDYKQCEMRSKTYGNQKTTLDVEHITYSCKITLSKG

>NP_819153.1 L-aspartate oxidase [Coxiella burnetii RSA 493]

MPTYDVLVIGSGAAGLGLALSLASEMRVAVLSKDDLTAGSSPHAQGGIAAVMNAADDSVELHVQDTLNAG

GGLCDPEAVRATVTQAKSAVEWLVQQGVQFTTDSKNNYHLTQEGGHSRRRILHAADKTGAVIVKTLAEQV

LSHPNIDCFTDHIAIDLLIENNVCKGAEVYDGEQQRSLRFHATHTVLATGGASFAYLHTSNPNRTSGDGI

AMAWRAGCRVANLEFNQFHPTCLYHPLANHYLITEVVRGEGGYLLLPDGKRFMPNYDDRAEMAPRDIVAR

AIDMELKKNNWDHVYLDISHRPADFIKKAFPTIYATCLNFGFDMTEGPLPVVPAAHYTCGGVMTDLQGQT

DMARLYAIGEVAYTGLHGANRMASNSLLECLVFAASCARAIKRSAAPIPRLAEEAAHPFFSLDSAGKPRE

DSRTVDDFIHQVRKIMWDHVGIVRSDIRLIKAKNDLQKLLQQINVVFPLQILSKSLIELRNVMTVAQLVV

ESALMRKESRGLHYNVDYPHPLAQARNTILHLNPSPTMSAIGEKQF

>NP_819517.1 arginine-binding protein [Coxiella burnetii RSA 493]

MKKNNNRAVSRFFRWIPAFVRMTGTFYFVSCLFVSPVFATDTIKFATEATYPPYVYMGPSGQVEGFGADI

VKAVCKQMQAVCTISNQPWDSLIPSLKLGKFDALFGGMNITTARQKEVDFTDPYYTNSVSFIADKNTPLT

LSKQGLKGKIIGVQGGTTFDSYLQDSFGNSITIQRYPSEEDALMDLTSGRVDAVVGDTPLIKQWLKQNGR

REYVLIGKPVNDPNYFGKGVGIAVKKGNQALLLKLNKALAAIKANGVYAAIVQKYFGQE

>NP_820223.1 sensor protein [Coxiella burnetii RSA 493]

MTPSIRTFLLINLLLSVTLITSLAIIGNLFLAHKDIQTQLDAQLIRSAERTRAFFSDYGIQHKDLSLIQR

NLLKTSARSMLTSSHFRLTKVQIEQLRADKEASNHSEFQIWNNQGKLLLHSAGTPTIPFSNGNEGLSTLW

LHGESWRVNTLYDPATELTFMVAERSNYRQQLENQLTQDSIFIMLITYPFLGFLIWIIVGRGLDTLKKVA

KEVRHRAPAYLEPVDLEAVPSEIEPLVSELNNLFGRLKEAFEREKRFTADAAHELRTPLAALNTHTQVAL

RAKTPEERKEALLKVLAGVNRGTHVVQQLLTLNRMVPEATLHEQNWMDLGKEAADIAAQLAPEAIAKNID

LELITPDDPPKVKGNNTAISILIRNMVDNAIRYSPEKSKVTIRIEKEDNRVILRVIDNGPGIPEELRERV

FERFFRMIGNQATGSGLGLSIVLQIAKLHNAEIALKTPNHGKGLDFWVTFPLIHNFCG

>NP_820583.1 heat resistant agglutinin [Coxiella burnetii RSA 493]

MKRLITLILVSLAIPPIYAGGAVDTPFLNSGPYVGLHGGVALPSGSDLNAGPVVGAQVGYRMGNVRIEGA

LSYYSNSLEANSDAKLRMTTLMANGYYDFNFNAPLVPFVGVGVGWVHAWRTNNGVLLNRGPDNNEFAYQG

IAGVSFRVSPRVTLGVDYRYLGWTDGNGSQNLIEMSVNYQF

>NP_819710.1 UDP-glucose 6-dehydrogenase [Coxiella burnetii RSA 493]

MHGTVVVGFYGMTHLGLVSAVAAASKGYQVIGYDPNEMLIAELKQKKYPIEEPQFPELMVDNADRLRFTA

ELDQLLAAELIYVAIDVPTDAENHSDLQPIRYALEQLQRRLRREQIVVVLSQVQPGFIRAAEFPAEQLFY

QVETLIFGRAIERATKPERFIVGCPDPTKNLPSCYHTFLKSFHCPILTMRYESAELAKISINMYLVAAVM

TSNLLAELASAVGADWQEVVPTLRLDKRIGQYAYLEPGLGISGGNLERDMVTIKQLAQQYSTHAELVKTW

LADSPYYKNWVWRCLQTEILSKTEAPIVAILGLAYKTNTHSIKNSPSMALINLLKTTKSQIQVHDPAVKK

EDREDTATHCDSALKALQSADVLVIMTPWLDYSKLTANDFLAHMRGRVIIDPYRVLNYEALIQAGFYVHT

LGVNSNALKKKEAAYA

>NP_819101.2 HlyD family secretion protein [Coxiella burnetii RSA 493]

MERIPGMRLIRPIFWLLFGMLLLSGCSKNQPVLLQGYVEGQFIYLSSSVSGLLKSLLVYRGDSVKEDQTL

FILDPEPQESELQEAKAKLTQEKENLENLVKGQRQTILNRIIAEREQAIADMIYAQQTLTRFQHLYRVRA

ISKDELDKATADYKAKKQIVNQTEANLAEAQLGERKHLILAQQAVVDAAQANVAKLIWELDQKTVVAPKN

GNIFDTFFKVGEYVPAGQPVTALLTPNNIKLIFYIPEPLLSRLKLGTVVEFKCDGCTIQRAKVTYISPEA

EYTPPIIYSQKSRAKLVYRVECAIPEKNALKFHAGQPVDVYLHEESLSDSHA

>NP_819777.1 UDP-N-acetylglucosamine 1-carboxyvinyltransferase [Coxiella burnetii RSA 493]

MDKLIIVGGVPLNGSIRISGAKNAVLPILAATLLIEEPVILSNIPHLNDVTTMIELLGRMGAQITIDERM

SIEVDCSQIQNVHASYELVKTMRASILVLGPLLSRFGKAEVSLPGGCAIGSRPVDVHIDGMRALGADIEL

VDGFIHATVEGRLKGAELNLGKITVTGTENLIMAATLAEGQTIIHNAACEPEVQDLANFLNKMGARISGA

GTDTIVIDGVDRLSGGSYSILPDRIEAGTYLVAAAMTRGHVRIRDVFPKTLGAVLEKLHEAGARVKIGEN

WVDLDMQGRRAKAVDIVTAPYPEMPTDMQAQFMALNVVAEGQAVITETVFENRFMHVHELQRMGADIKLQ

GSKALIRGKEKLTGAPVMATDLRASAGLVLAGLMARGNTIVDRIYHIDRGYECIEEKLAQLGAEIRRVSS

HVYAARYAAQKRWL

>NP_820080.2 hypothetical protein CBU_1081 [Coxiella burnetii RSA 493]

MRAYYYRLFIKAFKILNWLWENTMKKICVALLMLGNACAVSALAANASAHNLLTPAMNQFLLADNDQDQN

ADASNNQPADQDDNTSNADQNNDQNDQKDQSDDQMDNNSDND

>NP_820097.1 hypothetical protein CBU_1098 [Coxiella burnetii RSA 493]

MTTTFFKLSPKIDYQDRVENPRFTNSQDEEQTLQEWTRGTTFNKPETANVFIASKSKEKTAILADPKRSN

RDLPLPKENIDDSIKYVKNEFKKHGLVDKNISDYILKKGCLNGYVSLESEALRALLKVNPNNEENENDNI

NLNIRLGQSTYFVEGKTKVIYTQRFKIVIIGLDSNSKDIFPVEVKSTIVSKGDKIIHTCDKVTVEAEDAQ

KELFKSVFGMELHETIERKIKRILTALYSKLARIFNFFSPKKFFEPNTDEQSGKKRRLPGFKIK

>NP_820328.1 ribonuclease HII [Coxiella burnetii RSA 493]

MDAPFAKTPSNLLIAGVDEAGRGPLAGPVITAAVILNPEIIIEGLADSKKLSLKKREELYEKIITNCKAF

AIARADVEEIDRLNIFRATLLAMQRAINQLSIQPDKVLIDGHCCPDLPYETQAIVQGDQNVPAISAASIL

AKVTRDREMLKYDAQYPDYGFAIHKGYGTKAHLAAIHRFGITPVHRKSFEPVRQLKLFIPEE

>NP_819062.3 IS110 family transposase IS1111A [Coxiella burnetii RSA 493]

MKDIKILGVDIAKDVFQLCGIDEWGKVIYTRRVKRAQYVSTVASLKVGCVVMEACGGANHWYRTFMGMGI

PTQLISPQHVKPYVKSNKNDRNDAQAIAEAASRASMRFVQGKTVEQQDVQALLKIRDRLVKSRTALINEI

RGLLQEYGLTMARGAKRFYEELPLILASEAVGLTPRMKRVLNCLYTELLNRDEAIGDYEEELKAVAKANE

DCQRVQSIPGVGYLTALSVYASVGDIHQFHRSRQLSAFIGLVPRQHSSGNKEVLLGISKRGNVMLRTLLI

HGARALLRHVKNKTDKKSLWLKALIERRGMNRACVALANKNAPIIWALLTRQETYRCGA

>NP_819806.2 coA-transferase family III protein [Coxiella burnetii RSA 493]

MRRALLSRYQSEVLLGPLNGVIVLGFCYYVAGPIALQNLVGQGALVIKVERKPLGDPTRYVFPPAIFNSL

AHGQLSVAIDFNEEDDRKLLENLFEVVDVIVDNRSVKAKETDKLLTAYLDKPKKAHPLIYCSIDGFPDAK

VNRMPGLDASAQAITGLAYTNCSSPNNPLKVGTPILDITTGLLAANYILANLYLLSQESLPPETKQVIRI

AVSLAGTSVWLQANQFLDALGGRGEYFREGNRDRYAAPFSYYQTKNGLISIATVNEDQFRKFCTNVLEQP

EFHQRYPTIQIRIENQAAFDQDLNQILKNKNREYWLTKCKKYDVPAAPVLTVSQAAQQNFFKGLIKSTRD

GMPVVTHGAANSLFPFKRSLPAPTLNRDHNDVEAALRSKL

>NP_820379.1 enhanced entry protein [Coxiella burnetii RSA 493]

MKITVTTIGALISLMLGQPLIAAQVKSAGVLNPHSFIQLAAAKPAAQSGRLFVFDPKRHRWYAYQNGRLI

KQGRASGGAYGCPKNKSRSCRTPSGTFRIIRKGPPNCKSTRYPKPHGGARMDYCMCYHAFYCIHGSDHVP

NYHASHGCIRVTPADARWLSHNFIQYGTKVVVKSY

>NP_820689.1 superoxide dismutase [Coxiella burnetii RSA 493]

MAFELPDLPYKLNALEPHISQETLEYHHGKHHRAYVNKLNKLIEGTPFEKEPLEEIIRKSDGGIFNNAAQ

HWNHTFYWHCMSPDGGGDPSGELASAIDKTFGSLEKFKALFTDSANNHFGSGWAWLVKDNNGKLEVLSTV

NARNPMTEGKKPLMTCDVWEHAYYIDTRNDRPKYVNNFWQVVNWDFVMKNFKS

>NP_819567.1 hypothetical protein CBU_0535 [Coxiella burnetii RSA 493]

MKLVEHFSRLFWGLIGFFIVIAAGCSTTLERPMPVRSFAGTAACSQNAFLRKYDCSLSKIEVAAERGDPD

AQYALGYMYFYGISTVRDTEAANLWIRRAAAQGQPLAIRASHMIHEHEYPAMGAVQGPSLNPTSHSASPR

AALHYKEVDIHRANTRAPLEPLRHHLPAYRKNTSRHSNPVHDVLKKNSETPQEKDSTVAPPLSQRKGARV

AAPVAVTAMNAGLSAGEKALLNSKASYTVQLMASVDLKAIKDFVKRHHLEGQTHYFHASYRGHQWYVLLY

GAYKSQGEAKAAIQQLPQPLQKLRPWVKSVRVVKREIQLRKVV

>NP_819243.1 outer membrane protein [Coxiella burnetii RSA 493]

MIKWVTGILISLAILVGFLTILMMTPAGLNIGLRIAKKVVPGELNYSSASGIPTGPIAISNFSYRHKGLE

ITVAKLQVKWRLFYLLRGVLHITQLHADNIKIVLPLHTEETKSRLPFDISIHRGLLKNLSIKYPAYDPVH

FKTILLNQIEFNHALNGDIQAQITQPYPVNVYLHSKGTRDNYQLSLQTKSKDINWRMTGKGTRQWIELQT

HEAHTLNGHLNAFVKVYFDPIFKWDINLDMVDLNLRKFNRNWPQQLTVQLKTKGEHKIGESPDFTLNGML

QTPKAYMHIAGEHYQQWNLAWTLNVADFSSLLATATGSLQSHGTISGPTQFPIINGEATGHNIIFPAYRI

GALEGHWNIDTSFNQASTITINAQHVHTPVHRLSALKIDASGQPHAHHIKADIFIDNGEVGRTTVNLLLD

GNLQNKIWRGVFNNFTIQSQKFGKWDIDRSSTLTVTADHATISPFCLRSGPNHLCLQGRWNRIPLSTVNG

TLTVNSGDASIVKLFLPDTIQLHGRLVANFAISGRTERPSVNGTLQLEAGSIDFPQLQVSLTQVRGSVAS

TGSTINYRMEGYSQNQPIQITGQTRLDAPGRPTTLTVHGENLLIVNTHQYIIYGSGELKIDIVDRNIDIT

GTLTVPRAILKPTTFSRAAALTGDVVFIGADEKQTPWRTNINIKIILGDQILLDSLGAKGRLAGELTLLK

PPNQVMTANGRITIIDGTFTTHGRTLDIGPHSAVTFIQSPITNPMLSVRAIRTMKTSPIVPQLGGPTVTV

GLDVEGTLHHPEVALYSSDPNLTQADILSFLIFGHSANANTPANVNLLVDAVDTLNIGGGKTSAGGVVDQ

ITQGLGLTELGIESQTTIAALGGPTGPTQSAFVVGRYLSPRIYIRYSRGITTSINVIQIRYLISENWAIQ

TETSSLGSGVDVLYSIERN

>NP_819942.1 short chain dehydrogenase [Coxiella burnetii RSA 493]

MSYNRLIFRKITDLRFLIMPTVLITGASSGIGKALALVYASHQHALLLTGRDEDALNQVAQKCRQYSVEV

FIYAKDLCLPDAVQSLMKFIEEKQLKIDLLINNAGAGCYRNFSEIPFNSLEQMLRVNIDGLVKLTRQLLP

SMIKQGKGSIVNIGSVYSFVPVASQAVYAASKAFVKSFSLGLQAELKKTGVSVSCVFPGSTESAFRARAG

VVSDSKRFTLPSTIVAYKIYQGVKRKRLFIIPGWYNWLFVFLIQYVPIFWLPKIINFLAYRMRRIEKKPS

N

>YP_002332947.1 hypothetical protein CBU_0098a [Coxiella burnetii RSA 493]

MGCACEAQQKERKTIQYRRVLPKGSSELNKDDCFGVILLGFASSAQPTKHAGD

>NP_819564.2 COME operon protein 1 [Coxiella burnetii RSA 493]

MMFELFKEIFMKKIIQLISAVLITSLVFSAQAKPASEVIKNKLHRHAAVSTQKTGPVDINTADATLLTTL

KGIGVKKAKAIIAYRKKEGNFKSIEALSSVPGISQKTVARLIRNNPHRLVVNP

>NP_821002.1 hypothetical protein CBU_2029 [Coxiella burnetii RSA 493]

MRRLLVSFSIFFCILSGSITYATTQQSQQSSPQVNLYEKPQSNAKILQKLSPAERLIPIYRQKGWIKVGD

PRNGEVGWVNRDQYHEALQKYYQPDIQTVFIRAEHNEKGKRTIDVVAYKNGKKLTDKEAQQLYEQIKNQQ

AKESRYMRHVFWDMDKLMAQQMRAFSRWMDNPWNGNLSDFDPTVVQPLIILSPAHSVSQSAPAISKSKKD

>NP_820216.1 membrane-spanning protein [Coxiella burnetii RSA 493]

MRYLTLITAIATTTLLVGCAKRPQNYLTHARTVPAIRTSADMPVKRGKNYYPVPNAAPSVTQSPSLVPPG

SNLQRFENQTQLQQSFSKRTMATLQQSNQGSPVMAIAEKPAVAWSKVGRALQKTPYQILDQDSTLHSYYV

LDVKSTNNKITKTTPIYRVYLKAQGNQTQVILLNKDNQPAAKDVSQRILTAIQQKVA

>NP_819657.1 hypothetical protein CBU_0627 [Coxiella burnetii RSA 493]

MTPMRLRGDDGALSFLLFTTCGNDTITTTPQRGGIGYSYF

>NP_820361.1 membrane-associated protein [Coxiella burnetii RSA 493]

MMSHNSFSCVREGCGGEIILRPVIIILSTLLLMGCVAGPEQLGFSKQQWQGMSEKERQQMRIAHQEIQKS

LAAQKLIYNGPDIQVTIEGGMAIMPPFLQPYDFRATQFQIQPGRCQRVQLTSWEQIHSVDLSACYDGLTL

SLDSSRYDPTKSKGTLLLTYNPLWKRGFTYRGLSSSGYVRLKNTNITVKIIPI

>NP_820451.2 hypothetical protein CBU_1468 [Coxiella burnetii RSA 493]

MIKILLRSCLKWGMMTAASVIILFAVLISGVRMTVPLLNHQRDFFEHWASHALHQPVHIGQIAKSWYGFN

PALTFQKVIVTDPTQHKSLLRVSQLSISVNLFQSLIHWRLFPGHVWLSGARFNVFEGKDGKLNVEGMIAQ

KKQVFTDLGEMKKVFLGLLNQSNITLKNIDIYYHTAEGQLIPLTHLRLKVTNGLLHQQIAGVGSLSQTVP

TRFRFLLQGSSKNAELYLDIKNLVFSQWESSYFLQKYFKRVSLTDGRGNVQLWAKFKNDELESVQSVVKS

DQIRLSVAKSHSPLFIDQLNANLYWQRYANGWGLTADHVNLQMNGRQWPEHTFGVRVTHSQSGMRLLLKS

DYFNLSDMQTLTAEIGYQPKRIQTLFQQLKPSGILRHFTLLYSQRAQTPYYHIITDFEDLNFQPQEGWPG

GAHLMGAIDATAQHPASGWRFQVSQSDIQNNNLHWHGEGFLSLPAKGSPFVNLKGKIGLKNINAIKNYLP

SRGLSPHLRAWLNQAFSTGEITSGTVNFKGPLDRFPFLHQEGSFDAIANVDRVTLNYHSGWPALQNIKAK

VMFHNNQLRIVASHANISGNPLDHLKAVIPDLKDPVLSVSGHSTSNLANGFKFLKAAPLSVAKRMQSTTA

QGPMNLNLKLRIPLDHSNQEVRAEGQLAVKDGQFSLNNWGIMLDHINGNFHFINEDFSADPVFAKWLGLP

IAFHIATLNPSSQSPVLQFEMSGELAMQALQKKFKWSILNYLNGATTYRALLNLHGKDSQQDSLSIATDL

SGIQSTLPAPYNKLSKEVSLLTSHLSFYGNKLSKIKVHYQGSSSDAATSLLVSPVNEGWSVDIQNPLIAG

NLLIPNRLDDYWRGYFSRLYLPEFKAEKKQWNSQALPPVDLAVDDFHYGKKILGKLALQTSRTMEGLRID

KLAITAPLFNVDATGMWRTKIGRSQTTLSGSFTSPNLGDLLKQWKITHALEGGKGRADFSVKWPGSPDQF

KVAQLNGDIKLNFYSGRVTELTKTTESELGFGRLLNLFSLQSLPKLPLNLANFSKKGFAFNFFRGNFLLS

KGVAKTQNASLVGDVAWVQIKGLIGFANKNYDLHLQVVPNITSTLPLIVGLAGGPLAGVIAWVADKILAP

HVGKAAQVNYHIAGSWNKPDIILPAPAETDLSP

>NP_819577.2 LemA family protein [Coxiella burnetii RSA 493]

MSNLALSHDRPIIEGYNQKQGVTMTLLIILLILIILIGGYAISIYNHLIGLIESIRNNNKQIDIQLDRRY

KVFESLIEVVKKYMDYERSTLKDVVALRNQAQTAKQAGDNDARMKAENKISTILSGLHVVFEQYPDLKAN

QNAFQLQETIVSTENKLAYAKQAYNDSVERYNAKKKMVFPSMVVSAFPSKLNFDFPYWQLPPEKIIKQED

YKVNL

>NP_821017.1 hypothetical protein CBU_2046 [Coxiella burnetii RSA 493]

MRSALKARWIPAFAGMTVKKINLGINFSILGWVFY

>NP_820984.2 hypothetical protein CBU_2009 [Coxiella burnetii RSA 493]

MIRSGKMRKLINSIIGVALIVVIVLLVLPLGMSFWLKNNYPSILTRLSQAHNVSLKLINFDRGWFASKAV

IQVIIPNSEDKTTQPIKFTINQHIFNGPFIFSKNNHKVKLHCAKALVYTTSNDPNFTFHSSTLLRFNNSS

KSSLYASNVNVANGQEQIVLKDTNLEILYNPLTQRLVLNAVIKSALISEQQKTILIMDNITWRNDLHHAT

PLWEGKRSLSLNKFTYYLTPEQLIEVKNFILENQQNAANDTTTFTFSSHADSIKDTSLNLAPLDIKFSLT

QMNTAALVNLINTALNENHLKLNPQQLHQFHTPAINLLAQGLEVSLAHLTFGTEEGQVSVQGQLHLPAQN

QSPDLSQIMVNAKGNLQAKMPMAWLKKELSRIYEDKKVELDDQALTPEQIADQQIQYWINNKKLIPQNQD

VELTINYDKGKLLVNNLPSHAPQQ

>NP_821009.2 hypothetical protein CBU_2036 [Coxiella burnetii RSA 493]

MMFPNSLKSNPFNFKLRHSRAGGNPGAPSVRCACVRWIPACAGMTGFMFLVFTLGVMI

>NP_819206.1 type 4 major prepilin protein [Coxiella burnetii RSA 493]

MQRNRTAGFTLMELLIVIAIIGILIIIAIPSYHTYTRRAHFTEVVQATAPYKLGVKECYQMTNDLSECSA

GDNGVPPAIASGSGTGLVDAIEIQNGVITVTPQVKYGIEAKDNYILTPTPEREGLTWASSGGGVAAGYAN

>NP_820757.1 hypothetical protein CBU_1777 [Coxiella burnetii RSA 493]

MFVPVFIIAATSHTEQSIQSPKTTITIPALHDHLVQKHKTSLQMWNLLILNSEVSPNLNEI

>NP_820138.2 membrane-associated protein [Coxiella burnetii RSA 493]

MRKLPMPPPKGTTETESFKRLAKAYFKSPVIQTSLIFLSLQLLGC

>NP_819473.1 membrane-spanning protein [Coxiella burnetii RSA 493]

MPDKPKVTKMRTKPIIASNNSEQTELGQLISRLDSKVQSLNGLVTILQQTIFSKNQVHNTLNLTNEAREK

LEKDINEIIKWYWNLKTLSTALNELTFHMQSSKFLGFQKNNLSQTDIGILYQHEIAIDWTLRSIEQWCPE

LAESIKPYEEKEAKKYQEQIEQNQLEIKPYRPSLAERFGQTEFLNYKVSGASLWLRRLKLIFLIVGSIVL

NVILITLAVVFLMKPANITTMFTGAGLGILSTLLTMLSFTYIAQSWKR

>NP_819417.1 membrane-associated protein [Coxiella burnetii RSA 493]

MRNFIKIIVTSIMFLPLMLLTGCASIVNGTHQSVQVVTPPVSGASCLLSNDKGKWYLKSTPGYVTVHRAY

APLAVSCHKKGYLPANRLVHSKTKSMAFGNLVFGGIVGAGIDTANGAAFDYPDGIILPMQKLKNPRSLRK

>NP_819450.1 type 4 pili biogenesis protein [Coxiella burnetii RSA 493]

MEFIQNSVMRVNLSGFSLLELLIAMAMAAILTVIAYPAYRYYLVAANRRSAEVGLLRIASRLEEFHSLNE

TYQGATLSHLKIENESRDYLFQLSQLTTESYLIKAIPQAKQAADHCGTLSIDQSGHQKASESGCWN

>NP_819755.1 hypothetical protein CBU_0729 [Coxiella burnetii RSA 493]

MDTKVNYTLVGFFIILLGAASIVIFLWFSTFRHRQTFDTYLVYMHEEVSGISTQTPVRFNGVKVGYVQKI

EINPADPQQVVLTLKIKHETPITTSTVATLRSEGIMGTDYVALKALTATAPPLIAKPGEKYPVIPSEPSL

LMKLSTALQEVTKTIKELSDNVGKVFDEKNRRAISASLVNIQKVTKTLSDNSENIDATLHSMKQLMKNSA

KASEQLPTIMHQLQDTLANIKITARQFDRAGHGIELTVGDAHTAMQSMSTQILPTVQQLLTKLNATAAHL

QQLSIELQHNPSMLVRGKYPPPPGPGER

>NP_819491.1 thioredoxin [Coxiella burnetii RSA 493]

MAIMELTQSNFDNVVSQHDLIIIDFWANWCAPCLTFTKIIEEVEKDYPEVVFGSVNIEKEKRLAEEFNVK

SIPAVMILRDRVVVFAESGALPREGLRELIEKAKDLNTDILREAQKQKARE

>NP_820169.1 hypothetical protein CBU_1173 [Coxiella burnetii RSA 493]

MKKHFRLILILLLNISLGAAYADKLTKEVCWHQLWRPDGAYWSYFAPGKSDPTVECLAMGEKQCTIQIGS

DNPGASAPFYGGVNQPKGGWQVYKVYQSLDALFSDVNQGELKKAYNKCKNKK

>NP_819784.1 sensor protein [Coxiella burnetii RSA 493]

MITKKPIKKPLILLVEDNPFIQKIHVQLLKKLNCEVTLAIDGQSALALYSDKFDLIFLDINLSDMNGMEV

CKAIRQRTSSTPIIALTSESSKNKPRYFSTGINDFIKKPTTQAALKRVLNYWIQ

>NP_820562.1 trp repressor binding protein [Coxiella burnetii RSA 493]

MPFILVLYYSRYGATAEMAEQVARGVERVNKIEARIRTVPSVSPKTEATEPDVPKDGPPYVTHDDLKNCV

GLALGSPTRFGNMAAPLKYFLDTTSALWQSGSLIGKPAGFFTSTASLHGGQETTLLSMMMPLIHHGAIIV

GVPYSETELFTTTAGGTPYGPSHMAGADSNWPLTQTEKNLCQALGKRLAEISLKLKA

>NP_820473.2 virulence-associated protein I [Coxiella burnetii RSA 493]

MKMAANRMRPIHPGEILAEELGFLDKMSANQLAKHLAIPTNRVTAILNGARSITADTALRLAKFFGTTPE

FWLNLQDAYDIKMALKKSGKKIEKEVTPYDQAA

>NP_820355.1 hypothetical protein CBU_1366 [Coxiella burnetii RSA 493]

MKKWSLIVGGCLLFLELSSSFAASKPAAPSAGGMQEVVATLQQQIQQVQDSVPKQIKAQTDATQKQITQL

QQAMQKQITVLQQQIQQVQTQLNNEIKQLQKEIHEVEMIK

>NP_819361.1 enhanced entry protein [Coxiella burnetii RSA 493]

MKSLLITAITTAFLWGGVAVADDTLDDPAYTYALTLGDQDNPEDTADFVESSPNTAFLDENPYGLIADAQ

NVVQDYSRLPNQIRAPGERVFIFSPRILRWAAYDADGYLVASGKANGGAAFCANLGRPCQTPVGSFRISR

KGDASCVSSRFPLPTGGAPMPYCMFFSGGNAIHGSPYISNRNTSHGCIRVYPGAAAWLSHYFMRAGTKVI

VLPY

>NP_819774.1 hypothetical protein CBU_0748 [Coxiella burnetii RSA 493]

MPRNMKISLLLIVAACLSTLLILRKTTSDERPSQAQLNHRPNAFMRDVNYYQYDEGGLLHSHLVSPLITH

FPYQNSFQFTRPHYLIYTDKRIPWNITANSGKSQQGIKRIYLWDHVKVHEPPQPTEPETTITTSTLTLFP

ERSFAKTNDPVTITRPNAVIKATGMTSNLKKGLVHLLSHSRGVYEVEPAPGKKNS

>NP_819093.1 3-oxoacyl-[acyl-carrier-protein] synthase III [Coxiella burnetii RSA 493]

MLYLHGLGHYHPNNIIDNSFLESLNINTNNEWILDRVGIHERRTALPLNYIRETYNKNPTLAFQQMETSY

ADGAARAVHHALSRAGIETNQVGLVVAGECILQYTLPANASVIAAAAGIQSMAIDINTACSSFATQMHYL

NHLDESILPEYIVVVNPGFFTSIIDFTDRNTAVLFGDGAAAAVVSKRHESAWRLMDSHVDSNPKEWPVVT

SPTGGHFSQQGSRVQHFAIKTTVSEINYLSKRNAVNLNEIYFIGHQANLMMLQSAASRAGIAPEKHFYNV

DRFGNCGAASAPSVFSQRWDSFKSNDQVLMSVVGAGLTWGSLLFKHL

>NP_820133.1 membrane-associated protein [Coxiella burnetii RSA 493]

MTVEHFVAFCPNLFIIGLILTQFVRLFMKKTTVALSLALAITGGAAWAGKYTLVTPHTPHLQQGWSSANP

QDVNRVDATGGVAFLTITVDKQSPGPSPISNDGIKVNCAGTNVSVRPGSADKCTLEKGQYVTWADDGQKP

NHGATGMVEIN

>NP_820634.1 Icm secretion system protein IcmX [Coxiella burnetii RSA 493]

MKNFRVLGIASFLALGVASTSALADIDPMSGVIKAIKEVGLEVQALAIASKKSVSNMKYQLDKNLDLALQ

ADVEKNNALQTVKNNAGTNTQNQISGTLLQFPEQVINASQLNDAQMAATIKNRKNLIPNLTTAIPASDTL

YLTDAEDPLANTYGVAKPDSLYDNYFNFDSLFAPSAYNSDQQQAATTYLQYLTKPYQSLTDNIHFSELKD

NLNKLSAEKRADKLKSFLNNPAYQKFQLAVRSLIATKSLAIDNFNTLLNERVPVKGLGAKVGMPDDPHLP

KGYASPLQVENYIANQRINSPDWFKQMKTASPAVVAREQVLILAEIESQLERNHLDNERLLATLSLMALQ

GTKNSEMELQTNTAADLNKLIDQIGK

>NP_819649.1 hypothetical protein CBU_0619 [Coxiella burnetii RSA 493]

MKKLLAGFCLIALPFSFVWADTSLLQGAIDYSPNKISKFDWLQGYDGSQPCGSCTGSPSVRNGACGIDTS

VSGFQFQINTNGQAACNNIVVWQCPPPANSKNKPHSLLGVNPCNFNNAYAIGVACGGDDNKNPDKSCKLP

LVGQYQYDEGNKGTLKKVTYFK

>NP_820760.2 hypothetical protein CBU_1780 [Coxiella burnetii RSA 493]

MITCTGLNINNLAREASGMSRGNSSFSFPHQHPENSDQRLSETPAFVGNTSVILPSSGRFLSPLHSLLEG

KQHFNFDCEPTPTSLLPASSQPLVNFIDLSEETSSSNLQEAIPEEKEEDDPLLSQDITEKIIAYYRAKTG

ETELRIIGTTRANFRAVLDARKIVDKRNVPVLERLVDFYQEKKENEGLVGPRHKQFATVLRYIEKGVYCL

QQWFSNDQIRVLMAKISYYTFVQIKDMPHLVSPDSMQRAVKIYLRDGNENELCQRLESLKANFVRQSPNA

SLNSSNESTPRSETKSPSPLYSPSHNSHPNFFSLSGMSHSLQASGGSLVNWQHMNQLWNQLVSEKKEFLR

LTPASTFCSRVCELIDVSGWTWEDIGTLLAQVCNPEQAPRDLAAFLTQIKTDVIHLQNTGNFTQAQIREL

FMNYPKARTKLSEYIDEYSSANTVFPHFRDVHQVMELMKEWASASSYFPQLFSDHTYTTRWSQPYVESFL

EIHNSTLKIHLNQLIQPLQPYTNGSQPKRKHHTYAPGAN

>NP_819589.1 rare lipoprotein B [Coxiella burnetii RSA 493]

MWCNGIMRKIRLIIPLLMVIFISSLSACGFKPRSPNDIPPQLRILYLDAPNPYDPLVVQLSRTLRALNVH

LTQTREAAPVTLRIGHIGWETVIPTILYSSNATTYSYTLSVDFVVETQDGRTIKGPANLTLTRSLLQNAN

QVYTPNATRLMKREMTRTMVTLIYNYLVTGLAPPPPRVREKGA

>NP_819720.2 polyprenyl-phosphate beta-D-mannosyltransferase [Coxiella burnetii RSA 493]

MGRLSTMVAERARKSLNRDLKTVDGKIVLDLAVIVPTLNESAVIASVLESIRDNLPSVRYTICVADGGST

DGTVEIVKKMSESDPNIILLHQIKDRPGNQRNAGARMALEWLVKNTSHTVFTEIDSDGAHSAEELMNGVM

AVSLLKFDFIIGSKYLYGSKVVGRALYRRFISYCYSFLARILFSRRLRDYSNSYRFYSYETAKLILSRKF

SYSSPIYLLEILITCMSNGLKILELPSTYAERNTGNSKIIFTDVIKGFFIMLSIGFKYNFSRYKAFQSNQ

GMKYD

>NP_820790.1 type I secretion outer membrane protein [Coxiella burnetii RSA 493]

MKAAVYIGFFLLILFSEMGSYGNFALPAPCTTMVESHAYFHPHAPLTPLPFPRAFHQRAKHLSLSEAILL

ALRNNPDVISSELQRVVDKFALEVAHNEFEPQFTLGGTAGYAWRSKPTYSLNAGVSVKTPLGTVLETSYG

TSFTGGPGSATITITQPLLKGAGWAYNIIDFANAVDDERVARLTFKNSIITAVDAVIKAYRTLVEDYNKL

TIQRRTLLRIEQTVRQSELRVKAGKLAPSDLLQQQANLASTRLSMMQQQSSLDADYQAFLKTLGLTATAK

VIIDQRIEEAIYPIPSVDAAIRLALLNNIDYQTSVIQLRAARRAVISAKNQARWQLDVVASTTIGNGNGN

GGGSSISPISQGSAPITGGGGGPSLGFTLNIPINDVQAKAQIIDARIELEQAKLALEEKKEDLIRNVTNQ

INQLHNQYAQIKVAEHGVELQRRTLENAQLKLRFGKTTVFEVNQLQDQLLEQETDLVAQRIEFLNAITDL

DNTLGITLDKWGITLRY

>NP_820928.1 membrane-spanning protein [Coxiella burnetii RSA 493]

MFSYLSTCRAFPDLKKIINSRSTDAFLRGLARAMSSKMFIELMGKLAGYEETMAGNICSIPVFAITAYTS

YVGTQWWSNDPRELRSYVANRSSLYQTFLRILDALFRGIARGDTMSTVILLIGTLNSNLLPLAGVLAVVT

AGGGAFTSYNRTFSRTEYVRLMPRESDSDSEDVFSPYSPLSPFEQSTLP

>AAO90589.2 transposase [Coxiella burnetii RSA 493]

MDPPLKDGVIMRQHRISIFKKRRRSMKDIKILGVDIAKDVFQLCGIDEWGKVIYTRRVKRAQYVSTVASL

KVGCVVMEACGGANHWYRTFMGMGIPTQLISPQHVKPYVKSNKNDRNDAQAIAEAASRASMRFVRGKTVE

QQDVQALLKIRDRLVKSRTALINEIRGLLQEYGLTMARGAKRFYEELPLILASEAVGLTPRMKRVLNCLY

TELLNRDEAIGDYEEELKAVAKANEDCQRVQSIPGVGYLTALSVYASVGDIHQFHRSRQLSAFIGLVPRQ

HSSGNKEVLLGISKRGNVMLRTLLIHGARALLRHVKNKTDKKSLWLKALIERRGMNRACVALANKNAPII

WALLTRQETYRCGA

>WP_010891173.1 DUF807 family protein [Coxiella burnetii]

MLQANLTFLGKTSVGPIMPRSYFGNAGAIQAPRIQKTATGEYTITFLTSRDIKETQPPAVHINFQDEVNG

AFCSRLVKPVQFTYHPENSQFPWKILFQIAVQKGDGSYTDSNIVMITFAMANCNNQAVFNSCKNRVNAEL

NSCKPDDWECIADANTALQNCYLQCEPDLII

>WP_012569658.1 DUF2076 domain-containing protein [Coxiella burnetii]

MNQNDQQLITQLAERLKKAQPVAKDSEAAELITQVIGSQPDAVYLLTQAVLLQEAAIRRLQQQVSELQAK

VHSKKSFFSSLLGGARQSNSNESAPYGRTNPFGQSSFLGSAMSTAAGVAGGLFLFEGLNHLFSNHSTLNS

DSMLNNAGVSDILESDPLQGQGQLLDEGFGTGDSFMDESSSGLDSNDFDDGFGSGSDW

>WP_078377999.1 Dot/Icm T4SS effector CpeD [Coxiella burnetii]

MSDVNDYWYAKNEKGQIRPEFKNCTVEWKEDFVPPGRFNHCRLEKVTFKNIAVSEEDDFFKGAELTDCSP

SGATFANPSNFGVVKAKDLKSDLGKWFNEDDESEPWFEREVNKERKLYENNRKIHRFEEENKRLKSENQQ

LKDELKLLKARNSEKEAGASFGESSKKRSLLNEGLEEERTSEDFGPSTSSASTRLNLSIFSSPKRETDSE

TETEEESTKTKNQIKLT

>WP_230593246.1 hypothetical protein [Coxiella burnetii]

MSRNSTTSPLLAEEKKENYKTFSSDDFVSIHLLSEEVELLESEQVAAPDRLLALPLDMFKHLIPFIDVQT

KTRLLVASRSVRDLVKPTFSPAFFNVQINLHDVKVAQQALSEIAAKEIRRQNRLTARAYRLLLLIGALLL

ALGGWGVAADALAYSEAVNNFREQCGSDKGIDCDQTPPLTPHCVSLCVEANEKLGGEIFFGGMSIAVGVM

LCVGGALLLRNFYTTSASSVSVTQQQAVNDLSLVLLEREYPQQFQKIQPLLNQLENPSTFSKLESNLQNL

AIHLERKLKNSVENEENKENENQRSQNNQP

>WP_011109640.1 Dot/Icm T4SS effector CoxU3/CpeC [Coxiella burnetii]

MRKSSAKTNEDVSPSERDHLSELNPDTLKKIIHNLSARDSYSLSLSNKSLRNKVGAEFEHRAKTLNSDVK

KLHLTIK

>NP_820596.1 hypothetical protein CBU_1614 [Coxiella burnetii RSA 493]

MLNLYFQKLEKRIYEACFPSFSSKKAEKKKKKEKKYEKIIAEKKWERLKKKLDDFKSIIEKHLFDPIIYQ

GNGYVSFTKTSNDDVQPVNDDKTDKATISQANNPHIFISAPKKIIEASTPEDLDDCTAACCFLFNKTFN

>NP_820605.1 Icm secretion system protein IcmJ [Coxiella burnetii RSA 493]

MMALRNIQLSASDSNWRLFMVRKADTAFLAFQAKVHERDNYTCQYCGFRAKRYMEIVNLDGNYRNNRLTN

LATACGFCSQCFFLDAVGKGESGGGTLIYLPEMTQGELNALCHVLFTSIATESSSATEARNIYRSFKLRS

QIIEQQLGEGLSHPALLGQLLVDTKTEKMSLLKDALETKLRLLPDLARFSVQVEAWARDGLEELGSVYNT

RD

>NP_820193.1 hypothetical protein CBU_1198 [Coxiella burnetii RSA 493]

MSIRPHLDNQDIDNRELSKSQKQNRGSVVNYATLFNPGVETLRRDPYLWELIVAINSRETDQVEEVLEKT

EREKASERKPETIPNNRSGLGALISLSPTLLPDYPIEYAFEQWKLTPAPDMAYLEALVRHRKQVNMLIPP

DKAISDTQIVELAKGDESLESIVALSMNSKEPSNTRSL

>NP_820212.1 membrane-spanning protein [Coxiella burnetii RSA 493]

MRTSHQNQPSSPFLLPPDVLPLIFYGLTPVDLARLFVVAKGARPWFETFFASDTDYKAYLQLNIPQLFMV

IGNNLFFDYSLVWIARAGVWGWGNNYTGQLDFGNIHEMTKPARIPLDSFIPEMAPDDYVVQFASADFLSG

KARSLVATRQGQLHEYYWERISNTRSEFIQHRHYELNVTIVQLLSDYRHAFILTEEGGIYGWGFNISGEL

GLGDRFERSQPTELPQNFFPYLLERGDRIVQLNFADRYLVARTAQGHVYVWGFNYRGRLGLDDEQDRLHP

VELSWDFFPEWHPDDYIIQVVVGDRHTLALTQLGYIYAWGKNSYGQLGFRDQQDRNRPALLPWNRFPGLL

ERNDRIIELIAGGARSLARTEQGFIYIWGARWLLVRGDPEFRHHPMLLTENYFPGLRRRGDHLVELIAGP

DHTLARTEQGYIYAWGRNYYGQLGLGDRRIQRQPALLDPNAIPPLSQWRNAFSFIKSFREEKIEDTDSFS

YAPFSRK

>NP_820552.1 hypothetical protein CBU_1569 [Coxiella burnetii RSA 493]

MPITSLEFSRDRIMQFLEKLKFFETVICPISFFPELEASKRIRNKANLVLAIADSMEGTQKIKLEIWPFE

EYAARDFTQKDRLWYHSLDGLAAVLKELKELNHSISLSIIFMGETHDINDLSDDLTMSQKSHPGQLLKLS

TIRNKIRYFYDTNRESLRCFFKKVLTNCPNVTFQISNPPYLKKSIHDPKDLINKTAQDYGELMNLDIVDE

IIQKLKENISAYLPNSSEKEHKVTQDEINALKQKLKIQLKLDEDLKIYDKVMERIKNATLNSGLAKLKIA

EFIEITPPSSSSDENIHYLMRKFRARGPFQKSKTKTSAESFNNEQIMKISFELQQQLYDSYIAVIHKPPF

SFNQLTQRYNEILQEFFPSSETSPPAEVKSNDQERSSKRIRRESDENDASTQCYRALLACTQGLKNAILS

ETTAEEKVATLKKLSELLKRTVSEEGVIEPEITQQSARNGKEEKEPTPLENKSSEQHLVYATQKQLHKLE

ERIEFLEALYERSDDDTEASSLSPSERNSPNSSSFFSTSRATDLEQKQKDTSPRNNN

>NP_820616.1 Icm secretion system protein IcmQ [Coxiella burnetii RSA 493]

MTSFDLSNLRLNAKNEHLKQQLIECVDEQKAQFLQSAEVFYAKARRTEADYRHLCEAIIQATGQVLSAAN

WEESLFLRNTLKPIKKLYEEALALKEKLDGEQAGQAFTTPALTENKVKLYVSLYQSNGHDLKQWALQLAS

LESYMVGRPIYQNEADAMQAIRQKLSQLSEACVVVAVDQSKIISQENRSRKDRLGNLLTTVMPNAIKSEN

IIEFIHQGKRYHYVNQARELILKTSETN

>NP_820624.1 Icm secretion system protein IcmS [Coxiella burnetii RSA 493]

MQLANKLTALTKKIGANFTLKGRHLAYSEMFSDTGLLPGLTKRADQLASLCLGYGLGATYEDTENSLLGV

KVKFDEFTPDVLRLFCILDVIYELVKNSPSKDAVSLDELMYD

>NP_820802.1 hypothetical protein CBU_1823 [Coxiella burnetii RSA 493]

MPKLSNRDLQNLRDSSKWSEETINTIYKRLMELICNINNESKQEDDEQQELGELLRLENVNQQTVLIYLA

MKDPTKLIVLVENLKESHSREFMYAIKQKGKMDHNVLSCLLLYHPGKFPELIRLFDDERNISELFSGICS

VATYNLSKENFDVVSINKLEALFYCAPDQQSLIKAALEALINQISSPYYKIELLSQLITRTRSHRKQRLI

ENIYRDHLLSALLQVKKDVPSLVALSRLVSFSGKSYLKNVILSLKKAAPSNDEISYLSRLKSQAEGDEIS

LIYYIVNYGRNSGKVKKPGTTATSQLLFEPPKTSTKTSQKFKRFLNLFLPREKILEEKEMAEWPTNKRP

>YP_002332945.1 hypothetical protein CBU_0089a [Coxiella burnetii RSA 493]

MAQNQNGEPVSALLGSEYIIAKNATEGSFNFRMQYTDSNGGQLKTQSAQISPQKQNKFTVKGANIRVYVV

TITSKEGYTQWRLPNCKSTGGALEVKVFAKGSYKQITCRSLKR

>NP_819096.1 hypothetical protein CBU_0041 [Coxiella burnetii RSA 493]

MRDGTTRVNTTGMRLKTITDVLIKLSQSSTHYDSAIFTSHKVKKFNLTTNENEPIEIWVYDDTSSSFPIL

TEYLKKHLESLIAAANHIFNRNEQLKKHWHETLVYIKKALEERLKFNIDLKQARKLVRDLINEIGFFWIN

QQPTMITTASQLYKGFLTKLFEAVEIAIPDYKRNHILVIQQTGDIIKCEYYERGTSRSLRSNVENALIEY

RFAENTHESEKTKDFPSAHYRGKLGLCNVATCFSATFDANNETTSCKIYYRHASFPPIDLYPLYRQERKE

LKNTKQRYLKKKNEMEEGGKAMVNWQNRAFKTQQCREGIKLITTRNMEMLRDLMRKNRSELELLPAERPP

LIYTNISLLTVKQWMDKDFQEEQYHDTILAAERLRSRGNRENEDKFVPIMFNFGVNLQAKWQRRKSWWRP

SLPKEQVFENDRAFFKFNHLVIERLQLIASKKTFSFAKTQALPHLWELNEIASSWNRYEKTLDCLANAYD

AACNNYEKDPSKENIEYLQECEEKLSQAKKNLWKVADTHIAFYRKDFKQLIANRSNFSNDDSELQAILKD

WKMFNSLYLRDAWHDASFSIQTSIGNLTRVVGSELSINCKSADDRTTGFCRRFEGSEEGKDASSPHVRND

TDGGSIKFTGWAKTKKAKAIAKTEKKIAEAFSTHKLKKAVVAKSKIPTSHSLISSSSFWKLKAPDTSPFL

TTNLPHSLSG

>NP_819163.1 hypothetical protein CBU_0113 [Coxiella burnetii RSA 493]

MSATQLLNNAIKDLVTKTAERIEREQVDNFLKAIQKCVSPSPSTMFTTSKAESVSSKSEEEEHPSPAPGG

>NP_819656.1 hypothetical protein CBU_0626 [Coxiella burnetii RSA 493]

MSIDSDESPLEVSIYAKGNLAQDRTTNQDNKLRCQCYVSLQEERILSCVMEVAGPMEDVQQLKKVLEEVG

IENVIVPEEEIPNDIIKQGVIFAAKIGQELSRVDNFRDELKRHLGNRCLQEQRKSSLPSKIDSHINLIKK

YLEECPHIIAGISAWMSDLMDLLIQCWEELSKDKEVQEAITLLKENETDTKKGVVYFDTRLGFSLLQPVC

DVVEKKLRAENKDQFTVLGTLLQKLVVQYASFYEFELSKGQTDQRDLDGEASLKPSFNSEGLREFKTALD

YLSAETNQELSTLNPVARETCKRINSFNSTNLLEFLEGWYDANENESIVNEIVSYKWPSSMNDFIAEKQS

QSSDRTSIDAEKQQNFSQSLIDAKMLLKTYDGYSRDQFVQSTNHLSREKIKQFFIRNAFKIAKEKGLEGQ

ELIDYFRLVRQCDTVKSRYYFSFFGKSVGKSQASIFLEKVIKFAEDLQKGLKKKEKINLEKIGAQEKGLQ

WCKVHYRGIPFKDIMDTIEKDKEMSIEKDKKMSIENMLLSLVELKESYLTPLEKVKSKLSELEDNSKKSD

VDWELHKRLSLKERKLDEGLQSLNEVEEVLSLVRSKKGLDCQFKNEPLETFALRTIRSAISNLEQLPKKG

KQVKTVEAIKRNPFFVPISPASSTSSEVSSDRSSPGSEIPFFCLPPQFGASDELKSTSSTPRMNT

>NP_819665.1 membrane-spanning protein [Coxiella burnetii RSA 493]

MREEKEEDVMYVALHESEGRPHAHFAPSLGVSDPYVAVFLSWVETAGIFDRSSSQSVKAKIIQALLYIVS

FAGSAVTIELGWRFGDTAVGLLNKVFNKIFKTDVSFGDSEDWPSKFLGGIFAGASYITISGLCVFACHRL

VKHWNARTSEKKLMEEDHRVVRSTLGGFNNFINAPASSLAQVLFSVKYLSSPWNAVFALSAFVSLTVLNR

EAFNELINRAVSWCFGDDHQIKEERHQLTTRFKRARQLQWTSEDRSTFFEPQIPLPEKLKYLWQKAGGQW

EIIESPQSGAEEGTPLLSNASESSRDREWVNKIIKGTIVGMSPSPALVYALVVYEELNHRLGKGIAISAA

TLTGTGSFALFSVQMLKALYGEKSKVDKKVEYVSILMGFFATSPLLYVLMQYALDVPALWIFVPFLAIAS

TAVRSHGAKQLLDATIASWSEVSEEELFRKKISQLAEIFPKFSPIAVTKIHGFFSTPVDNDNDMPEPSDN

RLMTLV

>NP_819667.1 coenzyme PQQ synthesis protein C [Coxiella burnetii RSA 493]

MKQKITNHFTSPTMMEPLVDTTSTADDFFEKLEKETIEIVMETPLIYQFDRLNDKQLQIFAQQFYYYGYH

FPRFLGIIIWRSFDDKIRSSVASNLIDELGIISSENIDPEQSHINVFIQFAKAVGLNEADLKNSCPRDYT

RETLNKIENLLVHAPFMEAMGCMSPGLENALSHYSYNIYKGLQNQHRFTEESLYHFKLHSVLDKEHGNNL

KQSLLPHLKESKINRELLRKGALTAAKAQRDFFNGLQKDFSLL

>NP_819986.1 membrane-associated protein [Coxiella burnetii RSA 493]

MFFNVLQSNPLSNASMWLIKTFREHPRGTLATLGFFVGATSQLSSPSLGPDPEVFCLEEACQWAVFLLEI

AGSAGKIMIYSTLGYLLGRAQPEAQLEYARSNQPVLLNSQTLRFLESENIQNRQSLNLSGAWRSSDNSDN

DDDNDNAYLQVLRNA

>NP_820359.1 membrane-associated protein [Coxiella burnetii RSA 493]

MPDKTDSLTILETLIDANYQLEKELPPLIKKVDDEINKFKKLKELPSAEILHPAHEKKILKFVEEINTET

PTLQSVKLAIQEFVCLRKISSLISKFYFYQLRSYQGQLENFAVLVKLKSRLLINNLVKLLEVSVVKDAEL

ESIASVFLNQYLPATASATFSTITTIYLKSKGIKVEEIRRSIRENEIDYLDSAFKKYQEKISSGLEDNVN

PAMEIQEIEDTESLDGPSGEYFDALSVIEEEDEFFDASDKEAPNASESGKSSLFSVLVEWLKILLMLPIN

KLFNNYKSNAVPKEENNQAFFVKPTKHNLPIEKEPSRTWTWIRYIGKV

>NP_820393.2 hypothetical protein CBU_1409 [Coxiella burnetii RSA 493]

MSRTPNRNNPSSFFPSAEKKNRRLRSSSVRVMPAPAASQSASSKKRKRGTIDFEQLYDHFNKLKKTYYSK

FGMDFNPQIKRTEAAEGPFDVFISDLNINLSEKDTFSFFDAAFRFYFEATTLHYRNSNPTFTDCLSFLKA

RASWLSNCNLAISGFKNILKEFNFLRNENIEQKPREKMTEVLNQLTRLFNFFRDVKRMNLTSTVLEGLGD

ELKLILASDDLKSLIPNWINTQEKEILPYLPKRRSQNGSEALRFWHSLSEEKLSEHSPVLPTIDVRCD

>NP_820589.1 hypothetical protein CBU_1607 [Coxiella burnetii RSA 493]

MRIFSDKTGSNQVEANKRKAALEKNVILNQNIKRNPAIGTPCVPRTEQKASTTVYTPTFFQSLEQNKIQE

LRKKCVKAINKMNLPEKTHLEKFSTRTGLAMGAMIPASPNFSVEKQREKEKWEANERLKSCR

>NP_820647.1 hypothetical protein CBU_1665 [Coxiella burnetii RSA 493]

MKNKIVLRQLLAAKSIIPVGIYAGPRLDDSTLRNVKTYQALIAMLNNGLPLDGSELNCLIPKLGDLRAFD

LCCDLYYRVSPSLDTFVRMIRAAAKAGSFNAGEIIYNHALGWLSQFNYDPLYYSELVNARLFFLQKIGTA

EAVKEAIHFFLSSLKRGWCDIKAYPAVVGAVKQSFGSGYMDPFYQEIQGIFFNGIILLDKIDNAEISTGV

TREESQSIRIDLLNKMLEAASWNRDFERVDECFAELEKINGDDYRTYRNLMYTRAIQYRYELPEKVDDEI

SKVFGKAYERGWLVPYTINSFFKALKSYVEQGNISKEGKETCFSHAKAVFEQAERISNCDEWTEYWFIEL

AVICGHEEVRLPLIEKMINSNKDHVFKRWLGNIEVNPRHCMKSVRFTDFVNDQFWKAKNNKAKLFNLIHH

YLAHGKKAVNIKVIYSLIGRLGNFKYYLAEFLFLKVKNDPKHRYNQRLWEAIIKVAVQKRANLNLDILIT

NLIETVQTSPRNFQGKAVRLGENLCRVLKAHYAHNPDAEDPQLLSKLYDCIGERRVSMDDLNRSYSNLGF

FHQRLPAYPREEYKHEFSL

>NP_820668.1 hypothetical protein CBU_1686 [Coxiella burnetii RSA 493]

MPSSSKDLRKKLKNYCENQSEFDSKILNFPPQDVLAELVVIYPMPNAQADRIYEMLQQLALHCKTQQQAL

DNCLEDIRDLFQIIIEISDKDKSRKKDIVRFIELLQNFFKDNLLTGKEIYDLISIPSEAAKGSIFEQLDK

VGLTSQIKKLLGQHHEQITIDQQIAHLLVARFEGEELQNYLELVKQIDHFPQNQLVVDHLIKQIPLITRY

IKEKRQLTDVDLINSILDVFLQLPNTKSEIQNLFLTLIAILNPKEQLDYISLILMRLIDNRFSFSPEGID

FLMNSKLKDIMIGKFKLRAILIFNLATQNLISESITKTLVKKYIDNKGTLLDEIKNFLLNFNHSVNQFID

HFIKLTFLIYITQNNESVWRFLIKSKDREELEMKKKLFLESLCLHLKGSTDNNFPKNYADRIEKLDFSDL

LQIFISKSKLMDLTQLNPALRACGLETPNSSSRETSALNNSTLKFLKISVSTFAPVLHYLMTQGLLGETI

TKAFHSSELESALFNYIKTLESSQQEKTIQLAFQPISPFNHFFSDSTKEELKIIPSSINQSINSDEEEEE

KGFDEDFTDSDEESSDVDLPPPPTDFEKIKVQSYLIFDRLIMKFNPAETEESFRSQFLSVNEHANQFLND

KDVSWDKREEFCQAFRSDRPGSSADEASEYPEEQKPDLAVNCIMYTLWYEVAVKAQWASEYYIAIYDYAK

KPIREPSTAGLPFSEIYSTTWDAVLNYITENKTQETTSLSALLNQQGMFPSNSGESPAEQPGPTEEPTNG

YQP

>NP_820773.2 hypothetical protein CBU_1794 [Coxiella burnetii RSA 493]

MEADIMSRVDPGLKDLFTNLLSEIESNILDKGQLFKSATPSSITLHYLPGYLQKFSTLDYRHLSSDEEHH

LKKTIQSFIDELQCCPFNKKIYQGQLCQRICGLLVEFGKAGVFLPDMDNSLLWLEMSNNDMGKFTKRGKR

NKRTRDVDGAVTIYTANVSTLTKTESWNSIFEAQDILPHDCSESVESINKYLIAWKKEKGRNWEKSSRAP

AMQFLEVYVQQRLGFQPVISQNKNTTSSNSTHTLFRSPPPGDRKEPAPTFIQKSNISLTSHR

>NP_820804.1 hypothetical protein CBU_1825 [Coxiella burnetii RSA 493]

MLVSNTSKSYIKGAIEFLREQDKSITIGAKADTILNHLRHQVGNNKNSFAYYVVNYDRDKNFVKETGKTA

TSRLLFELPTRVQEVIGIVTNFFSPRRKRRDEPKTDNHNLEMTKK

>NP_820988.1 hypothetical protein CBU_2013 [Coxiella burnetii RSA 493]

MPSINLTTQQQRAWQELMRLEADYGLSLALRHFAANIIEQKFGLKISVGGSLYQQFDSEREKAAFVKKIR

TSGKWGNDTVLACVLDNLGYQAAIYLKGQPPYLTLTHNESTQLRMDIANDAVVDQKAQEAGSHWLNVNAS

NLSRSSVANKSIPGDCLYEALALQISYDRFTVGKEYPDTLPNGASNEEKPVESVNPSPEAYLTIEKAAQL

FEAEMKKADAFYAKAVETLQAATPSQLRALYDQARIIVGDTDSYLKYAPIHAVGAPDKATRQKFQKGIED

EHIRQLREDCLKALSRKQLIHILAREVWRNPAILQAPEIKPFYSQINKEQKQPALSEVGLFAPSACGKEV

TKEYNDSNPHLQLVS

>NP_821001.1 hypothetical protein CBU_2028 [Coxiella burnetii RSA 493]

MIEPPKELPPIFVTPQTSDESLQNLAITLSTFPIENREGVQVFEVPKIADDCRSYVGRGYEAYQDTTAKN

PQGPAWEPDSTWIKLESNNDFTCRKGFEKVYVYEGHYARQWGDGTILEKLGSTHWGATPVVRLIDPHAPI

VKKYYGEVKEIWIRPYQQGLVPIPYLRAQSSYRRGPFGKHHLFKSVYDKKEIVIQRLAKDDWKAHYQWAK

KQQQLRTRFFKPEEEKHQESNATFNPQASIAISLKAST

>NP_820338.2 hypothetical protein CBU_1349 [Coxiella burnetii RSA 493]

MSVFSVRFFLILIRGYRMSRQQSSKKLKIEGKINLTYEDISWIASTFKFEIDQPPEETLAVLSQSVSEGA

FKGASVLWVKFFHLAVESEKIRSEGKRLSEVMDRNPDEFFENLNRWLKNNYHNTHFVSNFFKLLQRQLSP

EEEPHSDQEEKNSSELKASWEEAERYTTFGLIDYITQNYLDYSWDNLSEIILTLMEGIIKLPLCEGIKFL

ESYFESTSDSVALFLFRCKFQKPDFESRQLTNLLALIEYISPLSDSDFIAFLKNQAADEANVPLSDNWVF

LCLYTECLNKIALRLLQCSPIDLENFLASTYDEDKTFYKEILAAATCHFDINDFEQLLCYAEEPEAEFND

HSEEHESYKFYIEKILLILKMVYPLRVDEEPPEPEAVFEIVEKIKSISTDELNQIIVMKRNFIDFIFLSL

QQKMGTDDFEKLVTDLANAHETNPDILSFHVDQLINSSINARPTLKNEFLEAAFYHIKIIYAQNPSHSKI

PALLNFLIRETLGTPVLNLDIIREEILKSWQKFTPKYREKRTYETFTKICIECNELEKTLRPKLKGIFNH

PTSPSTSPNGNTSSSLDFG

>NP_820476.2 hypothetical protein CBU_1493 [Coxiella burnetii RSA 493]

MSRKNIPEEESKSNNSFPETQGNPLVLQNSMVKPQDDSQAVKSKENIFKLLVLNEEIQQLPGLSIKISDD

KKRVYIVLWLFYTLLLDDQKSSISIEAFRSGVLYKLIKVLNCPLATADASILELETYLEQHCRSGEPSVA

GAIKLLRKQINELNYSAAQSLQYIKIEDANEYVLHATTPSSISTKEIIECIYSSSVWRQLSRTDDFTRLR

LSSLLIYFAKAIGKETISRSIVNPESNQETNSTDPRFFHQKSKKQKPYEPILNLLCTMLESLPPTVEHNY

GLDRNYICELIMHAAFKNDDPRSMLDNWTQLAKRGEEFVEHFQAVRTAWSRISNHSRSYIDQLPDIEIQT

TKDKTIKGKRQRFRYVLRKMASPHVASLGLFVPSCQYLGPAVGNRLTLTAMQSRDAGFYVITEQRISTEE

KREDAYPPRIVANTVAFNAKIKSKRYLVFNKWTKLHRQISDETMEEVIQNFQKELLQRHPQYPQYKEILY

GIETHPNPSPTIFSADSLLQGSNIMELEAIDRHIHIWRRTFDSAKVRLFCTKNVHDKQNKQTTCTCS

>NP_820513.1 membrane-spanning protein [Coxiella burnetii RSA 493]

MPLSKEEFQKQYSQQLQLRMYQCLQNKPESKSGEGEGEPRLTESQLIVLFLSLSAQKEGGSEREIDPDSE

LGKKINELGIYLQNNPNISLQREINQAHQLVTQLREKAYRAVIENNEGAGSAELSDDAHRAYAKDSERFK

AQSVYYTTLEQREAKSEEAHSLFVLWMIDPSCFCPNYYFYNGFYSPFDGIGLIGYGWAKLMEVQLQAISY

IGAHTAQAMQGILHGAAHAASGCFQLDGDAGEALLFVIIGAVVAGIFAGGAVEFISAAEDAAAGGPGKVI

AMGTTGAATGGACAALVALGLVSGPPGWLALGGVGITSLMVLAAAAIHGGLSKNSYELTEADKKRLKDRG

IDNVIAEQTIQTLNNEKEKIGYTMLGTSDHTRKKVIKKQIWDIKSGKLTALNLGATVQWRNENDLIKGYL

ELAQQSYSLKGGTEKLVRDLIVKHEIQNALREGRNNILDHTAIITSTNDLLRNNNKSDIGKSLMNALFPQ

NTPKNIEIANLLQEGNAELLNAISRLVDYARKKKLSRHSRHDKAADSAINIAHRLIYILSEGNSIDINTI

LRYSDDVMNMKQKSWKQYMPESLRKARTIATLGVWNVNNVKVGRFGGHRTEGGILLAQVRKIAKVYNDKN

FSTNPNVDFLRARLI

>NP_820840.1 membrane-spanning protein [Coxiella burnetii RSA 493]

MRNDDDTHSTLTSSSEQVSESKIVPKDSQKPSDPNVHQNGTDVTDSAKQNASLKEVTVVTLPPDLKALEG

DTHPTPMPSSREVLFNPNVHKNGTPLTHSVDPNGLSKDDEVTIVALESESKALEKELKKKGINYFKIGKT

IANVAYTASLCLVFYKLSAPRGVADGVISALVNAPSAAIFFDQFFGKVLINYNPKRVTKTHFALSLTGLA

AGNIAAIAGEQIAEDAVKGSSAWIFYGGLGVSLIYTFTSRTLGLPGAVGYLLNPLLGRCRPFPELEQFRK

DLEKNYLNPAVQRINEKTPERFIEVFYDTIKENPNKWTGYDIYKNTIEKAFQVGGLIAVQHFFGLFQQLA

AEGWGKISPQLENNCGLNSISAATTAFFYYLLVLKLPPTFRKSLEQIWILGVHNSRTLPAKIIKTALLLA

TLGGAGVAAYFSGSGMAEETARYDQLAQNGTLPFCVNNPPLSPWEGIDGNVNELWMTIVAGVGCNFTGML

FLFNPILDFFIKKSHENPWCIQSMLAVLESAPILNSNQLEKELGSWIPLIQGKRPTAIQAIENKRSLVKI

AENTNLPRPKFWFCGDLWKCSSNDENTRLLSETEDPVPSALTA

>NP_819064.1 hypothetical protein CBU_0008 [Coxiella burnetii RSA 493]

MSNFQKQQKAVSDSPALSNFFISVIFKSVTSIPIYLSSNWRTTNPPKPQPLKLTQGRATFVS

>NP_820154.1 hypothetical protein CBU_1157 [Coxiella burnetii RSA 493]

MVMKRISLSKLLPFFTILLTLTFTGCASIHHGPQRSMSSAISKYRGYAFKNLGPYFKRAGVSYPPKDISL

LVFKNSHRVQLWAKDRGPWRYIRSFPILASSGGPGPKLHDGDHQVPEGVYKIVGFNPASRFDLSLMLNYP

NSADRYYARLDHRHDLGDDIFIHGSDASIGCIPIGNKAIEQLFVLAYLVGERHIKVIIAPDDLRYHAPIY

GAVHPRWLPQLYAQIRQALQPYG

>NP_819746.1 membrane-associated protein [Coxiella burnetii RSA 493]

MPKETKTDSIKQEFATFSDDVAKLRGDLSSIIHKLTSFSKTEASMMKNNFVNRGKDVIHSVESKCKKKPL

ATLGYAFGAGIITGIISRKIARRK

>NP_819934.1 hypothetical protein CBU_0921 [Coxiella burnetii RSA 493]

MICYIHAMSHFKKALMDPTAFYKEPKAVLVDSSLTKKEKLQILRQWEYDARELMVAEEENMLGDASSSSM

LSRILKAIHELDPSYDGTKSSGTKHGGFSSETNR

>NP_819434.1 hypothetical protein CBU_0395 [Coxiella burnetii RSA 493]

MKHSLKLIILVIAVILLSACHRETLKGSGKVVTQTRQVPQFEHIKAHGDVKLFVTAGKPQQVAVKTDDNL

QSYIVTTVKGDSLEISTKGARRLVPSTPIVIEVSAEELESLATAGSIQTEVKGIEDDSFDVRASGNSQLV

LEGRTDKASINIEGNGQIDARQLITKEMSLSVSGVARAIVHAERKLDVKVAGDGEVIYFGNPPFLNQSIF

GKGKVEKGSAQLRKGLVN

>NP_820953.1 organic solvent tolerance protein [Coxiella burnetii RSA 493]

MKQGKSFIFYCLVLLLCGFQQLSSAVTASIAKAIKTTDRKQRVSETLPTGLSYRRFYQHIAHLLGWVPAP

DLVCRGYFKEPLILTEHPHPGPATKEPAIVTAKGPSMVTAQGVSILRKDVVVTQPGRIVEADKAYIYRDS

KTGHVTKIILIGHVRLHEADKRIVADKGTLTLYPKTAILMNAAYHIYNGEPYFYKFKYPFDAWGIAKHAV

RDASNVITLRHATYSTCKPTAPAWSMSATTLVLNRNTHRGEAYNMLLHIGRVPIFYFPYFNFPIDNYRKT

GFLIPYAGHSSSSGWFFALPFYWNMAPNYDLTLTPEFMSERGLNLQSLFRFLSTKSSGTIYLNYLPNDKV

FQQFRETTLSKFPPSVLAEHPVFIPYVDKLKKMKNQRAFFSMNETTLFNSEWSSRVILNYVTDPYFFQDL

GGQLGGSSLANQLLNQIDLQYNGLHWQFMGMLQAYQTLHLISQWTTPALDQYSRLPDFNIVGYYPDIARH

VDFNFNAEAVNFDYRSDFVPDKPRGQRFHMRPGISFPFYFASGYIIPQLWADATAYNITHFQPGQAHTSS

RLLPIFDIDSGLYFDRNFHLGHRSFIQTLEPRFFYLYVPYQNQDRFPNFDTVLLPFSFEQLFALNQFTGN

DRLQNANQASFALTSRVLDAQNGSPILTANVGFIYYLENQRVCLTPGCTPSNYHYSPIIGELTFYPFPYW

SFTGSLAWDPNLGQTNNTSVELAYNNGGKKADIRYLFVHGNEDSIVTPTTLIVPGNAYSQNTNHVISSGA

WPLLKKWNAVGYWDYNITERRTDVYSIGVQYNTCCWALSFSIRRTYAGLKVDPNGALQRQYDTAYGFELQ

LKGLGNLGTAPISTVTVLDAMNNGVSNDVR

>NP_819951.2 hypothetical protein CBU_0939 [Coxiella burnetii RSA 493]

MTKGPFMRTFIKILVSVVAALLLVSTISVVVLTKLVNPNDYKDRIDHYVYNQTGRHLMLKGNVGWSFIPW

LGVDLKQVELSNPVGFQGENLANIGEIKIKVRFWPLLMGRVELDKITLDKATINLIKNKNGKDNWAQWSH

SAVTKNNSKNTADNNKKMTAPVTELKIAGINVCNTTINLINQKSSSTTSLKKINLTTGPIGNAVNFPVSM

QFTLASNQSKNTFNATLTADADLNFQQSNYRLSNIKFNGTLLRPTLPPVPVNIVTNLTANLNQQNISISP

LNSKLTNMQLNGKIEINQLQTTPRLSIKLGTANTDLKQLLTTLRGTSPLTGQLSFDTVLTTSGNSKEDFL

SHLNGSGKFAVQNGTILGININEFLAQANALLTHRSAPRLKNSKTTAFSNLTGTYLVKNGILNNNDLKMN

AAPITAKGLGAINLVNNRINYTLTAAYTKSGAPPQFEVPILIHGTVNSPTIRPDFSALASKILTNEIEKK

VKQYTGGIKKLNLNNFFH

>NP_819523.1 phospholipase A1 [Coxiella burnetii RSA 493]

MGKAIRKVGLFVIGILVILPFQSGAHTHTKTTSQLKNSRNDCRHIKRYDKKTKHYYNVVVCKHATPPPPS

FEEKLQEILSNLTPAQQSLLEKRLARQRQVTKTRAGISFYEPTYILPYYYTGRPYHSIYEGNTPENQQLD

HQEFKGQMSFQFPIWYDMFGSNLSLDISYTQLSYWQFYAKSQFFRETNYEPQLFLSDHFSPNGLAAIGIN

HQSNGRGGNLERSWNRLFLDFTFTGAHWMVDVKPWILIFKAESSDLHNPDIYRYLGYGRVVFAVTFHRQV

LSLMLRNTVESGFKRGAIELSYSFPIHGLLHGYIQFFSGYGQSLIEYNHYTNSVGVGIIISNWI

>NP_819078.1 hypothetical protein CBU_0022 [Coxiella burnetii RSA 493]

MGTNQTKQRETQPPAYQKLQEKLAVLNSLKNNSYFDELERLNSLKEVEITLGESTLLLGQVSAGEKETIL

SLNDQLAGEYKGALLVHFKKYLLPEISQTKNFFKEEFEKFEDILKTDSKNINFPVQRVAHSAFNTLKGLH

DELLELENETILSTASFDTQYSTLKKTQKSAYDTCQNLKAILKKNRRYDFGRKIYDNLAFIVRWLGKQLG

LIDPQKSIDIHPRYKRTVKALASVPEGLVKHGLFQTSNQDSDLNLAGSMFSPITPRKGG

>NP_820652.1 M23 family peptidoglycan-specific endopeptidase [Coxiella burnetii RSA 493]

MKRCLVVIIIAFLLCGCVESKNFAPVVNGWLQPKARLGSYRVKQGDTIYSIAWAFGLDYRALAAANRLSP

PYRIEAGQTLQMTIIPRGAHVSGRFAAAPRWQSPPPLQPVAHWRWPAKGRLIGYYSVGMAGNQGINIAGH

YGEAVRAASDGVVVYSGAGIRGYGNLIIVKHTNTYLSAYAFNKRVLVKEGSRVRAGQKIAEMGRTNSGRV

MLHFEIRRNGQPVNPLRYLS

>NP_820868.2 type 4 pili secretin [Coxiella burnetii RSA 493]

MRFSMIKTAIGLIFLLSMSYSLAERDPFILSPSTQAAAMKTLIIPIHYADAKLLAAFLNSKKSGILSKFG

YAAADVRTNQLWIREEQTHIYLIQSLVSQLDIPIHQVLIKARIISISDHNLHSLGILFGTKTNNAQSNDG

LIEDKPNSTIQFGVADIPIIKFKNGQLLDLTLSALEQEGRAQVLSCPELMTNNRQTAIIESGEAIPYQEK

TGQGNTSVTFKKATLRLKVTPVVLPGKRILLQLNVNQDKMSSFLVNGVPTIQTQQLTTKVMVNNQETVVL

GGIYEQSTQQEEAGIPGLHKIPILGGLFRHRQRSSERKQLLIFVTPQIISPYEKKSHQYLFNRPHGRGQN

QRR

>NP_819244.1 outer membrane protein [Coxiella burnetii RSA 493]

MKEHLCTIFILFCWFFSGLAWAQQLPAATNTLEVQIAGLPPTPLENVFKRLSEKHETIKHDFVPATIRKF

YEEIPKEIEEGIKPYGYFKPHIHGYIRHPYPHFWFGHFTVNPGPRMQFTQVKLQITGPGAYDRAFLHLYE

NFPVKAGDFFDSEKYESAKNDLFNVAAGRGFFKARMIRNQILVDLKNYRSTVIIVFDTGPRFLFGTTDFS

ATPFHESFLHRFLQYRKGHYFSQDKVRRTREGLANSDYFSTVVVTPEPQKAQGLYVPVKIHLDMQPKKQY

NFGLGFGTDTGPRALVSTNLRWINPYGHRFNAYLRASPSNSALVANYIIPGSNPATDLYTFSAAFLDQDQ

DTGKGRSGRLSVSYQTNVGNWQQIISLTALRERYNLRDLPRTNAGVLYPSISWEHRHADNTPNPSRGHSI

VATISGASESVLSKTSFLQTRLDTRFLFTAWERTRFIIRASVGYTAIKNILNLPLSLQFFAGGAQSMRGF

SFNSIGPGRGLFVGSFEIQEKIVKNIYLASFIDVGNVSDRLFNEKLKIGVGPGIVLLTPVGMFELTIANA

ISEPKKPWVIQFSMGSVL

>NP_820520.2 peptidase M23 family non-proteolytic protein [Coxiella burnetii RSA 493]

MQRILRFLLLFSIAWIVFPFVFPFLHGSSSRGLSAGPRETTNDGMTVSMMILPEAFAATPTDLKQINHKI

ETLKAVLSKEKNKRTFFLKRLKTAEIASGRIRLQLQKTEAALKKESQLLEKLNHDQTTYQAKLATERAEL

ADHLRAAYMIGREPYLKLILSQNDAQRVSHLLMYYHYLSKGQLSAIHDLQSTLARLRQNQTSIQAQTHIL

QNLQKQQSDERAHLEALKQERQQAIGELNNKIKTKNQRLAELLADKRLLEQTLSRLEKQHQIEAIMKQDF

AALKGKLSWPTKGSVLPYFGIPIDQSELKWDGILIRAPEDQPVYAVAGGKVVFAKWLPGYGLLLIISHGH

GYMTLYGRNHNLYKKPGDMVQKGDLVATVGRSGGYEKPALYFAIRHNAKPLNPSMWCHRGENQ

>NP_819463.1 hypothetical protein CBU_0425 [Coxiella burnetii RSA 493]

MEGVIYSPTVVDSKPHELTLLFPNCNKVTLIGDQSLSSQIENLHKILGEPKAESEAIKTAFENHGETGQY

PLISLLRTLRYHLNATDFEYSETQPIETLTFKRTSFYEGLRRRPGLAWESCTIHFIPHSALTELLVQDNS

QLFRTIDAILVKTSETDTVPNELQQVLQSLKSDSLILSTPSTIQSWFKNPPQKTDLKTVYDTTITLDLSQ

AKNLIIAPPQPEEKQIEKTPKEKMQTLAENLTKEVTTKKLSINGIPTVINEWESEQNLKKEIPSLWGEFV

QDIKVLKNKEPEIVIQTIKGWIEGLYYNDSTSSDKEAAIRVKVFLTHFLMIRILPLLNKDLVELFQPLFY

GNHSHLGGKDYYYKLLEKHTTQNTELSKVLQSYSGYKAPRANWDQPSIRVKDVESWINNIKPLSAPPTSS

NSWLRSPFWQPSHKTQTPEEKVEQKFASKGPSAST

>NP_819814.1 hypothetical protein CBU_0794 [Coxiella burnetii RSA 493]

MKIIKLVENIYKDLEATNKKFSAAITNEARGSELFKKICDKYEVICNNTQTNAAKGRMAELLKAYYLEAW

LGKLQAEENYLSENPENENILLENDKKLEGITKSLEELLLEANKFYPSIISKKSKSKFYSNFLSDCKETY

NSINKYSNSWEKISNRTKANYYYNFSEDLIEKSKESHSDEETFLHRCSVYLKFCISFYDEAEDQVSKEET

EKKLEEVNSRIEQLKTARIRKRKLLSISSIDEDKAFKKKIGEKTNEPPLFTESNGSNTKEKETKEALTAV

NLLNPYPEPKSTDDKETAHCSYRKPDERSTVSNHPSLSTCWAGIFSSVNSNSGSFDPTNRNSTSDEHDKR

RLPPKMRHKAGRDKDELHLQIDPGIKMLEVAATAINLPNSTPELKNTGDETAHCDYRKEQPSVSNPPSLS

TFRSGTFFHANLNSNSSNPTNEERTSECGKSHLLPKMRQKARFR

>NP_820409.1 17 kDa common-antigen [Coxiella burnetii RSA 493]

MKKIATIGATAVLAVGLTGCTPGNNIPGATLAGTAAGGLLGAAFFHGEGAWIGILGSALVGGVVGNFVGK

RMDEQDRINMARAVTNTPVGQQASWTNSHDVTYTVRPIRQYHQHHRYCREYQTLVTIGGQTKKAYGTACR

MPDGQWKIIR

>NP_820539.1 membrane-spanning protein [Coxiella burnetii RSA 493]

MPSDRNGDSGTLLPLPGLWPTETTPLIERHKAATILDHFYSFLRKLSLPASLIQSLFSNTFFIMEFFKLI

ELDNPVIEGVAISMFALGTTGSFISTVSPHVTKERHSPHPRVNQFLSDCLYIFDFEIRLQEEVNQNQEIW

FYFNDDQLKEDFKKEITDINELRETLKTLVDETYPNHTLAFITQDELEACNNDLPTQQRIYYWKKQPVNT

NLTHVFIDIHSHMEGMLGGWPRAIIFRYLQAILPFMSGVLSVNHVLQHFIGGEEDMPLPRYIPMQVFAIA

IALFKLSITWERKFKRGEKWWAEFYLRVKLGKSSICEILKICLTSPHNLLALCTYTASMFFFLEKGAIDQ

ILKITCRISTACEHDFNALNPPPLSGCKLPVAYSLTAMSTITLIFTQIITDIDRYLQNQNAASGFCEKVT

RLFQCLCRRDQNRETNPETLLEDLSSDSRQTTQKHFKVWVAVIAANTLEGCQQGLGIFTSMLSFLAHVTQ

QSPNAVLKTVAAVFAAGASLSYWGWLHRQGKDFLTKTAETVNQIKDYLPLSIFSCPSSHDSHEPIVANEA

PVNYAPV

>NP_820185.1 outer-membrane lipoproteins carrier protein [Coxiella burnetii RSA 493]

MNTIKILIGLLGIFLFSLSGIVSAQSDATTQLSQLLSNFRTYQAKFNQITFDGQDRVIQQSHGRVMIMRP

GRFRWETDSPTKQIIITNGKTLWVYDVDLSQATQQPLAQKTNINPASLLSGSVKDLKQKFTITISPTPDA

ATFQLVPNLGKSLNFNWIRLKFSKKQLTEMTVLNNLDERSIFQFSQIKVNAPLSSTLFEFKPSRGIDVVK

Q

>NP_820559.1 membrane-spanning protein [Coxiella burnetii RSA 493]

MRPEHKKPIQSVSNENATSNVTVDVEVHESASINIPEFTPTKKEVNEEPQPSTSAGGEEIETIQNKPKSQ

SINLSESTPTKKRVNEEPRPSTSADGEEIEIIQNTTKSESISLPVSAPKDINEEPQPSTSVSKEKKEVVV

NLDVVQDAPLSSTNSSEFIRSYGTINEGAGTSQNAAEKPTEIVLDVSHLIEQLRAALTPSTTRQYDADSY

FKIVSILMSLSLSVGPALLSLGLSLQDDNLPSGPTRWVFAVLCGLFSSVQSGMVSHMTFEHYLSQLKFLI

HTLKKRELSQNRLEEGIEAPANVNIYKKLLKKSPSFFVYSLYEASKWIALYFASALPTSLAEQVSYLKNS

AFKYYLRASTMIVGYTALENFLTTATALLPSLNNKHILNGEKRRFRDAVITILKQNCHDAQDKNTEAAQR

LFKLISNQSTAIEPHKILEDLFENSRGNPIFLQDTSSYLQWALTLVFLTVYVGSSSGDYRAAVQDPDNTD

AEIIPSILGDITFASLVAQIAAGNLISAFYNRFLIFCRHEWPSLLINSGCTLLTIFTVPGPMAVSVHEKM

PFFITYGLTPLGTMLDYTIFSSELSQDIYLRYKFRSGGESYRHQRAVIETFYQHFQEKLEQLSLEEFIQW

IKISLEKSPDLFKGYEAYLYKVLPWAKEGNLTPSQILDILNDKPSEAMKTINAAGHLGLKHKSEKLFIAA

MAALVSGATFIPENNLFFRIILDHVFPATSITLGLTFNFCLFPKRIPEEKQPLLPIVQNNEENNSPQESN

PSCWSKTISFFKYVMLLTVPSLVGAVTKKVAHFALQDIFNNDQVEENSEIIGHAATTATSFVVVYSMKH

>NP_820667.1 hypothetical protein CBU_1685 [Coxiella burnetii RSA 493]

MRTTISTVTDNPMIHLAKLGHELIKLDPDLISENSWSESNLLKQLESLVILDNINKKKQIIEDLFKKLKE

NPNPSDSEKLISQLTLLHSFEIGVLKHLIEEQEKLESLQKSPKDRIKETIRLIGLKILLYNADKYLEEKK

NIVGGKISRASFKTLEKFDLLHIAEDLLAEKKEYFSLWNENDPSQGLKKLLDRLIESADSYKAVAEENKK

ANSNASDKSDFMELLAKEIDIPPSKTLKALDELKKRARNLYQFIYQNTHIYTHYHNVPPCVFNFDQEISK

FQQNYIKENILNLLEQHIRDLEASGLSLDLRIDLETIIKITKKFTDKLIERDNFYEKITELRLVEFFSDF

SKMIKTFIDKKNSSLDKEEALAAFEEKWSQQFPSFNLNDDDSETNNDTVGRDNLDKKYISKLTEIFSNLI

RNIKHLISTAKKNSLANNSSADRLNFCLFKVVKNCHRSVNHHTDYSISSIKIK

>NP_820797.1 membrane-spanning protein [Coxiella burnetii RSA 493]

MSRLPSKTKYHSSHRSLNRKTPLLQRSSETNSLRESGIETASSQLSLAASSYTPIDEEMTELELKIYLFL

FTRALNHKLGYTQENNPDDKAQKAGIDINLDCLNYLLEILYQNLEPQLEKGANLSYHKSSTARKALEYHE

TLNSRLKKKIAKDAEHKNPLTLLRILNTKTTSISTLIGTGGGIGITGAGAIAGSAAAGIGTAVTVGVLLF

YLCWRTTSEYWKKKNAEKLFEHTDQIDNENIIELVSALSAFYIVKEFEHKNMQKASLKLSLNPESLFEIF

QNIYHNKSFRLPSQLPIQKELKAIAKQAKIDADKIHTHLIEYVNTNKAQSISINLYALIIPSFAYNSEES

PTVVKTVAALHAWLWALDKTLDMKDFLFRNSFEKRLEKVRAGVVKTMKTFKEEVNNVLLETDDSSSGVSL

LADEDKTDRVKEWVNKQKLPSPGALSPIKSASHLALFSSLREQKDKAVNSSGSRLSLRLGN

>NP_821023.1 hypothetical protein CBU_2052 [Coxiella burnetii RSA 493]

MPKNTNPDPRLSLIASKWYEERKKTCPWIKAQKPTENTIGNFVNLINNPSDELINPTLEALLLEDTVITE

HLPFSGRIGHLLRASKNLLNYEFTHQEVQELFKVLSLKELLYFSNMNGVFKKLREFNFTKEAIFEAVLKI

KEKRENEENVSLLEELMQCLQKEQEPSRVPVRMETPPQSPDFNSGNSISTSNPSTLLVSPPPPGDLLKER

DEPMNSPSLGSPSFFFSPPPDTLKETVLLRTPLFKPESSEGDSLLRSNSFSLGNSLFSERNQILCLSSEG

GSNEENEDNEDNDLVNAFLK

>NP_820798.1 membrane-associated protein [Coxiella burnetii RSA 493]

MRPIHMAGEEDLQPINLKISIYLESLNTERPSRYMNLMKERLQKKMEQQVKKQKSKSRSKAKEILTNNSK

IIATATSVPLTVGAYGILVGSATPFLASQTNSFIQSSVHRGFENLTGSGLMSTIMNPISNGLSSVLGNTA

EAAITIATPIAVLYFVPTLFRLFFYVSKKMVHLLANLFDSTESHSLNSEYFESGLPLLTLLCNLSTLAII

DDLTLRLKAVGIEKISINLIKLKNKILNKENSNLNVEEKMLYDLAKKNSEKLIALALKIGEEKIFKRIQS

LESVTSLKAQIQKFIPKVDMWANGKIDFWITVMGALIDKESVQARETFFNTLQSISTLKTPNAPNPHSLF

QLDMEPRHKQREKFSPTDQPTYGRQAF

>NP_819144.2 tol system periplasmic component [Coxiella burnetii RSA 493]

MRLIKMKIKTLCVSSALAALMLSAPLTWADAPVEDISAQPQPTKTTVSPSETPETAIPTAPVSLPTTQTD

LTVTHRLARLEQQLNNIINMNLPQQISDLQQRLAQVRGQLQVQERNLELLNNQQRSFYRDLDQRITQLKN

LNSNNSDSSNDNSASSSQKPSSGDTSNTNNIQLQDSNTYRQALDLLTKKQYDKAQASFQNYLNDYPNGSY

VANAHYWLGEIYLQQKDRKNAAHEFQTVRDKFPKSEKVLDAKLKLAIIDAEDGKIKQAKEELTEIKKQHP

ESTAAQLANIRLQQLEEVDSATTTP

>NP_819642.1 outer membrane protein [Coxiella burnetii RSA 493]

MIKRLLSAICLSVAMIWSVAAVAQTVGLVDMRQIFQTAPQIKDINTRLEKQFSPQREKMTKLTQSLQQNL

QKLKRDEAVMGKKEAENLRKEIQNDESTLRQQQQQFQQELFVAQNKAMSDFMSKVNGAVKRVAERENLDL

VLPKDTVLYAKNSKDITSNVVSALK

>NP_820610.2 Icm secretion system protein IcmK [Coxiella burnetii RSA 493]

MSKVRKMVIRKIFLTLLIGASIIPVLALADNAAFAYGVRSLITDPAQNSPSTQPRTADKLRQNQQAVIDN

LLNKARSTANPTSQNEASSASSSGQNPGLSDDAFANTIRNMMPLSPDQIRTLHYLFDQSQQAAAAAPGVP

PKPTSASVIVNLSPGATPPIIRLSSGFVTSLVFLDSSGAPWPIQAYDLGDPKSFNIQWNKKDNTLLVQAL

SHYKAGNLAVVLEGLDTPVMLTLMPGQRAVDYRVDLRVPGLGPNANPDLDGLPATESPELLNVLNGVPPS

NSKPLTITGGDCQGWLIKGHIFLRTRLTVLSPGWISTMSSADGTHAYELQTTPVVLASQRGQLVKLMIEG

L

>NP_819354.2 outer membrane porin P1 [Coxiella burnetii RSA 493]

METTTKLAIGVSALCCLASAAFAGGPDIPMIDMNGFHIGLGFGYKSYTYDQVGTVTVTTNGGTVLSVLHP

VSASITQFGPVGELGYTFASDWWIAGVKAQYQYDNVRSVHIMDAPLVGSNYSYRTRLGSHLTAMLLAGIK

VNEANAVYLEAGYSTVWGKTTLFGPGPVAVSMKNRLNGGIAGIGWRHYFMNNVFLDLSYDYALYRSKSNS

VTLSSATASAEGTAIGVSGTVQNPKRVAINGITATVNYLFNI

>NP_819660.1 FKBP-type peptidyl-prolyl cis-trans isomerase [Coxiella burnetii RSA 493]

MKRLILPFLSVGLLLGTTAHAATPLKTEQDKLSYSMGVMTGKAFRKHDIKIDPQTFSMGLSDAYLGKETQ

MTEAEMRQTLQQFEKQSLQKMQHKMKQTAQQNAEKSRAFLTANKNKPGVKTLANGLQYKVLQAGQGQSPT

LNDEVTVNYEGRLINGTVFDSSYKRGQPATFPLKSVIKGWQEALTRMKPGAIWEIYVPPQLAYGEQGAPG

VIGPNEALIFKVNLISVKKK

>NP_820612.1 Icm secretion system protein IcmL [Coxiella burnetii RSA 493]

MTIDAEQLALQKNNLYRDNYRRVMAFLLASVVITVSLLAVLSYQIITTPKPAYYATTTTGRVIPLQSLDM

PVVTNTYLLQWAALATRAVYNLDFENYTKQLDNASSYFTPTGWESLTNAMKSSGAIDSLKNNKLFMAGIV

NGPAVILDQEVVHGRYSWRVQLPLLVTYTSASIQQKAHFIITMDIIRVPVIDAAKSIQINRFSAVRG

>NP_820626.1 type IV secretion system protein [Coxiella burnetii RSA 493]

MSRFQLLKTIAIATFTTLLLTGCATRSTADEPPLAAMGYVNLNSLPPGSGQINNIREQALRETATMLGAR

GALALRAVHIDAALEKQATYLDHLFDFNQLLLKHNVLPPVIVESQANLNLADDDTIRTADKTYKIVADAR

FVTAPPTWRSYLWLSYKKPDLPSATLLPKDKSEAQVWNFYLKQGWQNGLQQANEIFAANLNRLKRDYLGM

VLYRKLLAQGMITSPVVAKVDLGVTGDANQIRINDEIMRITAQSALQPDSSHWNPVLTDGASSP

>NP_819077.1 hypothetical protein CBU_0021 [Coxiella burnetii RSA 493]

MSRQPSLTRQVYRDKKNQPLNKLLVFLFNLRSDNLPTGICPGPFQELAHSVPPEKRNKLIAQFGEWANFL

YPKKEESAQSAPINGFISFLMEVILINPYDYNENFYKKLAEIIKIKKSKGLTWHDCLKDWLLTELTALDI

TIDDLLKQNNLEQYGQFHERYDKLTSNFLSLNKSLPLSELEIYIFFSNKNRELKKLCGSLASMEKCQQNL

ELIPETELAAISRQLSILDQEQCSAQIIASRQSEIPSSISSLPETSPEDWQRSIRIPIESINMSIVGAIQ

VGLAHYKKKRGLTDGDIKNGKSKSYILWDNLQRDIKENPRRNYYPHLIVAFEKYANKENSAKTCILREMA

SRTSTLNDSSPFKFKKNTALRYFGRLYQMIYQEKSYELERLLEPIFVLTEKIDGLENERISRESNSSENE

ANLVNSLAAFNQITRKCIDIDLPRLRQNNEIISLIKDIDLMINRLNFPLSVEVINQIMTLLNGWDNSINK

LKEAKEVNPTLMARLSGSRKALNEKLKEEYGKLLQNLQGNYESIKANPNFTLDELKAFEKELNKVKQLNI

TELSSLGVNSNTIKDQIVKKEGQLRSYIEAYYNNHIKLKIIEMQNLLRNEKKNFEEISSTHQNTPPHVQG

TLSSFSEQLKTSLDTLSSISAPSDFSSTDVIQGPLDTLRSTQTNLLGQCDLFTISLEFEEEKYNTFSIKL

LHWVVEFARKIRNFLSNLFPFIEKKDKPRSPYEVTHTALNTTREAVSSLHVPQGDKQLENKLGHQSHSHA

EQKISTATLNTQGMFRTKSVKETDVNKELDFEPIASFTK

>NP_820935.2 hypothetical protein CBU_1960 [Coxiella burnetii RSA 493]

MSPYFASLHTGYLSRGVFSVRIQRIYYPDQLSVDSTVTLDSRNYHYLLNVLRLREGHKIILFNGDGNNYG

GRITRIQKKQVEITIDSKEAGRTESSLFIELGQGISRSEKMDYAIQKAVELGVNRIVPLITERTSTKPKT

QKLAHWQGVLISGCEQSGRCCIPELMQAQSLSDWLSQKREGLSLVCHPNAQNSLADFKTTSQRITLLIGP

EGGLSDDEVQRATQSGFQVLFLGPRILRTETATVTALSLIQAYFGDLQRL

>NP_820618.1 hypothetical protein CBU_1636 [Coxiella burnetii RSA 493]

MTWKLNEIIGDKNRTIHNGVTRFVYPGFAVEDNGYLISEFNRFSRLVNLQILTEMEADRPIEAVIPVYSF

KEVPPRERFPLRKGVQEDRRKRMFQALLDAFMSVLKPGQERLTVCFDPIGEELIASLLTKEIFQEARKSN

FDYAKNKWQEQLDNQYEIKIPAVLSTLNWALNEVFRNHRSSSSSSSQNLLFTIDFIENDFEKILNDHPDF

ESLNDEQKSTFKREWLAIGKAFEQFFFKEENMRGLLEVQSLSEKKGIHLRLRTSEKRSDELTSVINGILP

ESPNLIVTQSSSISSSSSTPQGRQPLGEEKVSEKKKSTPSRWNMDQIPDLPPGKYTVSLGSRDVFSDNYH

PSEQPKRPKLNADRSLTRNPRGQCAAFFPHRQGVTTSGLVL

>NP_819641.1 outer membrane protein assembly factor [Coxiella burnetii RSA 493]

MPSIMKFRKRFLFAITFIIFCIPFTLTAYGFVVRSIQIQGLQGISADTVRSYLPIHEGQEYTAQRGQRIL

QSLYRTGFFETVRLARRGDALIISVKERPIISLIRISGNKAISTKKLRPILNKLNISEGQPYDPTKLHEI

EVGLEQQYGAMGYHAATVSTRVVKETKNRVALYIHINEGGIAKVGSIQFVGNRAFSARTLRNQFKLTTPG

LFTWISHRDRYSQVKLEEDLQQLSEFYLNHGYLRFRVVSHDVQWSPDKKHVYITIHLVEGPIYHLSGYTI

TGETYGFKDQLYRLVKLKRGDIFSRQVIINTNKAIGDFLGDRGYAFARVNVIPTIDDQQHLVHLTFNVAP

GERVYVRRISFFGNQRTDQEVLRREMRQYEGSLYSLSKIEESKRRLELLGYLSDVKYTPQIVPNSPDQVD

LNYHVKEIAAGRASIQGGYSDVYGFLYGASISEPNFMGTGKYVSIGFQNSQYQQNYSFVYNNPYYTTWGL

QRGFSIYYSRVKPNTKFNLSSYVEDGYGADVTYAYPISERNSIGFGYGFEHIKISQLDPAIAAPSVLAFL

GTTNGVQNTSRDYNQFKLNGGWTYNGLDRAIFPTKGLYTGIGLEIGVPVLKSSLGYYLATYSAKYYQPIW

HGFILNLLATVGYGDGFGHDRLPFFKNFYAGGIGSVPAFSPNSLGPKNRYNSFGAIGGNLETIFGVHLIL

PQFISERVRTAIVFDAGNVFQVPRFPGDIAVPARATIPDPEAATRPQIIQNDRFSLKNLRPSLGLAVEWY

TPLAPIDLTLAFPLNRRPGDNFQAFQFSFGVSL

>NP_819109.1 type I secretion outer membrane protein [Coxiella burnetii RSA 493]

MKKKFLVFVLILFSLVAAPLLYAEDLVQVYCQALASDPTFQKAHADWLSARQNLPIAMSGTGTPGSGLFP

YVDITAGLDRTFQRIEAGSSSVSGYFNQHNYQVTVTQPIFNYATWKAISSASFSVKAATATYIEAAQDLI

FRTAKAYFDVLDAYDQLQFTLAQKESFYHQLVTAQEKFKVGLIAITGVYDAQASYDQAIAQEIQDRNNLD

NQLENLRAITGQEYRSLTSLKKSIPLVIPHPRNIDAWTAVAERQSFAIQSALYTMLAQRETVKETAAQRY

PTLTGTFSYGAQQRGFPPSLSGPPTTGEFLDTTTTTATAGLNLNFPVFQGGFVTHSTRQEEYNYLSASDQ

LNFTHRDVVRQTRQAYLGVDSGISKIRADRQAIISAQNKLEATQAGYVVGTRTMVDVLDAVTSLYQAQQQ

WATDRYSYIINIITLKQQAGTLCPHDLAQINTWLGKAVRFDVEKPVNAKVFTPSTTLPHTVKHVHSPKGV

QRATRSHRPLIKASRHHTSPVHSASHIETVHAHYTIQLFASRTLAEATAFKNKHRLHDLRIIHQNGWYKV

LSGHYSTRQAATIALHRLPSSLQKLKPWVVRVPKVQSTSIKPLSLQKKATHLPPPR

>NP_820609.2 Icm secretion system protein IcmE [Coxiella burnetii RSA 493]

MKGCKMAEFSKKFLQSAKFRVIAAAVAAVALIAVVGVIWHHKATEDAFKSTAEVSSPPTIESLPGAGNPS

DAYVKTQNIQNAQQASEARKGGTSFVPTITRPSFLGSEDQFEQDQPSAPTTDLKKRNCPIKKVVYMYKPN

PASCTVDNLKLARSAGVTAEELVCQSCSCPSLRLAGYTAGELKEVGYSTVELRKCGFSIAQLQAAGFSAK

DLKAAGFTAAQLKAAGFSAGELADAGFTPDQIKAAGYSSAEMQAAGIQTNNPDCDLAALKKARASGVTAA

ELRQKGCGLAALKAAGFTAAELKDAGFTAAQLKAAGFSAKDLKAAGFTAAQLKAAGFSAKDLKSAGFSAV

ALKQAGFSNADLKDAGFSPEQIQAADKVAKVCDVEALKAARAQGISAKELKEKGCGLAALKAAGFTAAEL

KDAGFTAAQLKGAGFSAADLKAAGFSAAQLKAAGFSAKALKAAGFSAHDLATAGFNASQLKDAGFTADDL

KAAGFSDQALSAAGFPPSSGDCSVKALKKARMAGISATELKEKGCGLAALKAAGFTAAELKNAGFTAAQL

KAAGFSAKDLKDAGFSAAELKAAGFGAKDLKDAGYSAQDLKAAGFSAAQLKDAGFDAQALKDAGFSAADL

KNAGFSAEALKNAGFSAAQLKAAGFSAGALKAAGFSASQLKAAGFDAKALRDAGFSAGELKAAGFSPEEL

RHAGYSKGDLLRAGYTAEQAGYPPSSPPGTEVSQSAQRPPLSADNSAASVSGLNNSQSSAMPSINSDSPE

ARLRALQKLQQEQLNEQQRRDVEQQMQGQMSLQAQKLMAGWSNDSGQAYQVALQQPATTPVGGNVSSQQG

AGAAAKPTGPVIKAGTIMFAVLDTGINSDEKSPILATIVTGKLKGSKLIGDFSRVDKKVLLKFNLLNVPS

FDHTFGINAVAIDPDTARTAIAKSVNSHYLLRYGSLFASAFLSGLSQGIIQSGSTEECFFGICHRQYSKL

NTAQYIALGMGNVGEQYATVMGNNFNRAPTIRVPGGTGIGLLFMSDITLPQPLPAHQNT

>NP_820254.1 OmpA-like transmembrane domain protein [Coxiella burnetii RSA 493]

MLKKIVIGVTATAAFGIGAGALAGGSVDQSYNNTSGAGFYVRGEAGYGLVDKKSGTSKVNFTGVTLTENS

HTNTKKSRGFNGRVAIGYAFNPYFSLESGFTYYHPAYRDVNIAGSALPLFSSVQAEGRQKINLYSIDLMG

KATLPIDNFYAFIEGGVAYVHTKFVAFTETGTAVSPLLPPVTSSVAVKVPSSSKGYIRPKAGIGVGYNIT

QNIGVDVSYSRVFGQGKINNTNYLPNLNAVTLGLTYKF

>NP_819350.1 outer membrane protein [Coxiella burnetii RSA 493]

MSVKKLVTLATLTMASVGVTSAALAGGPDYVPAPSYAGVYLEGNLGYAYRPWRKDATTVVGLAKQARLLG

SSSRGNGGFTFGADLGYQFNQYFAVEGGWFYLPKVKFTTTGVVNGLAAGTYTVKSGMAYAALKGMAPVYE

NTYVFGKLGVAYTYNRANAGLPTNKIPATFGSRSRFWNPLFAAGVQYYFTPNWSVNAQYTFVPGYRNASS

KRFVAPVTHLFTVGLGYKFLM

>NP_819143.2 peptidoglycan-associated lipoprotein [Coxiella burnetii RSA 493]

MSRKFTDKIKGIVMNNLVKNSGLAVIALATLNLSGCKHHPAGANAATGLSDGTGAQAYALAEGKGYQGQL

KKDSEGRIINPLVAPANQTYYFDFDSTQLRSLDLGAIRVQANYLATHSTAKVRLEGNTDNRGSREYNIGL

GWRRDQAVARILEQEGVAPKQIDMVSYGKERPAVMGNNENAWRLNRRVNLIYEAY

>NP_819783.2 ComL family lipoprotein [Coxiella burnetii RSA 493]

MFYNGRICLALNPEEGPMKKILFLATLLLILSGCVRKDVDPYQAYRGKTSAELFTSGERALAKKDYSEAV

KNFEALDAIYPFGPHAEQAQLDIIYAYYKNNDTSSAIAAADRYIRLYPRGRNVDYAYYMRGVISFDLGLS

WLQKLARVSPVSRDVSTLQQSFTSFATLAEVFPHSRYTPDALTRMRYIRNLMAQREIMIAEFYMKRRAYV

AAANRGSYVVQHFQGSPQVAKALAIMVQAYRALGLPKMADASNHLLQTNYPHTLEARKLRKA

>NP_819950.2 hypothetical protein CBU_0937 [Coxiella burnetii RSA 493]

MTCDRVVTPPEILRGETTMTSKLVISALGLCVSGALSTTLASTPATTNQQITKRIDYLQAQINELRTQQK

KERQKKKAPYRKCSKKSCKYHSSRLSIGPYLHKTPAFDGSDLIINVPTVREDARLLLLQHQLEEECRALG

VPLPEMPRVVFSGKLEGQTSYGSTYAGSRNANINFSGAEFDTYVQANPWVSGYMALDYDPDELADGSRVF

MNRAFIMIGNLSRFPFYTSIGQVYVPFGRYSSMMITTPVTQALGRTRARAITLGYQQTGNNALHAELYGY

QGLTNNFSRSNHNDQWGTDVGYEFSNGDRVSGEIGASFISNLADSQGMQATAFLDNETLRHRVSALGVYG

SLAIKPVVFIAEYISALKSFDINDVNFANRGARPTAFHTEANYTFKTGSKPSSIGIGYGHTSQALGVGLP

QDRYSVFYNVNIWKDTNFALEYRHDVNYTRNAISTGTNPTPAKVVADLGKSDNVVTAQFDLYF

>NP_820613.2 OmpA family protein IcmN [Coxiella burnetii RSA 493]

MRIKHVLSVKQTSHSPYEAKRDTGIASFRFVSYGLLLALGMSLSACSSYTTVSSAPVYTIPAPKPSLAKI

RAHYIHRLQADGVQVIKLGETMRFVLLSDCLFKPDSANLRSDYRPTLKALARLMKTYDKVNVQVAAYTDN

NGHIERQQALTTRQAQVVASFLWSRGINARLAYAVGYNRKNPVDYNGSSHGRFNNRRVEISFRFYPEYVP

YA
