# Supplementary material for: Unveiling promising immunogenic targets in Coxiella burnetii through in silico analysis: paving the way for novel vaccine strategies
Source: BMC Infect Dis. 2023 Dec 21;23:902. doi: 10.1186/s12879-023-08904-7 (PMC10740251; doi:10.1186/s12879-023-08904-7)
Supplement: Supplementary file 2 — Supplementary Material 2 [file 12879_2023_8904_MOESM2_ESM.docx]

**Supplementary Table S1.** The distribution of unique and common proteins (in total 178 case) from *C. burnetii* using VFDB, literature review, and manual and automated reverse vaccinology strategies.

| Number | Unique proteins | | | | Common proteins | | | | | | |
| --- | --- | --- | --- | --- | --- | --- | --- | --- | --- | --- | --- |
|  | MRV (109) | **OPPA** | **VFDB (32)** | **OPPA** | **MRV & ARV (8)** | **OPPA** | **MRV & VFDB (20)** | **OPPA** | **MRV & VFDB & ARV (9)** | **OPPA** |  |
| 1 | NP_820793.1 | 0.72 | NP_820605.1 | 0.57 | NP_820953.1 | 0.54 | NP_819463.1 | 0.41 | NP_819641.1 | 0.44 |  |
| 2 | NP_820237.1 | 0.48 | NP_820193.1 | 0.68 | NP_819951.2 | 0.60 | NP_819814.1 | 0.66 | NP_819109.1 | 0.42 |  |
| 3 | NP_819100.1 | 0.41 | NP_820212.1 | 0.42 | NP_819523.1 | 0.58 | NP_820409.1 | 0.39 | NP_820609.2 | 0.58 |  |
| 4 | NP_820680.1 | 0.46 | NP_820552.1 | 0.50 | NP_819078.1 | 0.18 | NP_820539.1 | 0.39 | NP_820254.1 | 0.70 |  |
| 5 | NP_820398.1 | 0.65 | NP_820616.1 | 0.32 | NP_820652.1 | 0.58 | NP_820185.1 | 0.55 | NP_819350.1 | 0.45 |  |
| 6 | NP_819949.1 | 0.55 | NP_820624.1 | 0.38 | NP_820868.2 | 0.49 | NP_820559.1 | 0.48 | NP_819143.2 | 0.70 |  |
| 7 | NP_820396.2 | 0.53 | NP_820802.1 | 0.19 | NP_819244.1 | 0.47 | NP_820667.1 | 0.30 | NP_819783.2 | 0.29 |  |
| 8 | NP_820960.1 | 0.63 | YP_002332945.1 | 1.06 | NP_820520.2 | 0.60 | NP_820797.1 | 0.66 | NP_819950.2 | 0.52 |  |
| 9 | NP_820397.1 | 0.68 | NP_819096.1 | 0.40 |  |  | NP_821023.1 | 0.34 | NP_820613.2 | 0.72 |  |
| 10 | NP_820235.1 | 0.46 | NP_819163.1 | 0.26 |  |  | NP_820798.1 | 0.49 |  |  |  |
| 11 | NP_820135.1 | 0.44 | NP_819656.1 | 0.45 |  |  | NP_819144.2 | 0.68 |  |  |  |
| 12 | NP_819970.1 | 0.38 | NP_819665.1 | 0.44 |  |  | NP_819642.1 | 0.38 |  |  |  |
| 13 | NP_820252.1 | 0.38 | NP_819667.1 | 0.27 |  |  | NP_820610.2 | 0.50 |  |  |  |
| 14 | NP_819381.1 | 0.38 | NP_819986.1 | 0.39 |  |  | NP_819354.2 | 0.63 |  |  |  |
| 15 | NP_820267.1 | 0.62 | NP_820359.1 | 0.27 |  |  | NP_819660.1 | 0.58 |  |  |  |
| 16 | NP_820283.1 | 0.61 | NP_820393.2 | 0.55 |  |  | NP_820612.1 | 0.48 |  |  |  |
| 17 | NP_821052.2 | 0.76 | NP_820589.1 | 0.61 |  |  | NP_820626.1 | 0.42 |  |  |  |
| 18 | NP_821049.1 | 0.70 | NP_820647.1 | 0.31 |  |  | NP_819077.1 | 0.40 |  |  |  |
| 19 | NP_819254.1 | 0.27 | NP_820668.1 | 0.39 |  |  | NP_820935.2 | 0.42 |  |  |  |
| 20 | NP_819315.1 | 0.67 | NP_820773.2 | 0.35 |  |  | NP_820618.1 | 0.41 |  |  |  |
| 21 | NP_819762.1 | 0.75 | NP_820804.1 | 0.34 |  |  |  |  |  |  |  |
| 22 | NP_819410.1 | 0.87 | NP_820988.1 | 0.53 |  |  |  |  |  |  |  |
| 23 | NP_819757.2 | 0.52 | NP_821001.1 | 0.42 |  |  |  |  |  |  |  |
| 24 | NP_820182.1 | 0.47 | NP_820338.2 | 0.40 |  |  |  |  |  |  |  |
| 25 | NP_820633.1 | 0.41 | NP_820476.2 | 0.4 |  |  |  |  |  |  |  |
| 26 | NP_819437.1 | 0.28 | NP_820513.1 | 0.54 |  |  |  |  |  |  |  |
| 27 | NP_820832.2 | 0.53 | NP_820840.1 | 0.46 |  |  |  |  |  |  |  |
| 28 | NP_820878.1 | 0.43 | NP_819064.1 | 0.49 |  |  |  |  |  |  |  |
| 29 | NP_819822.2 | 0.53 | NP_820154.1 | 0.44 |  |  |  |  |  |  |  |
| 30 | NP_819191.3 | 0.56 | NP_819746.1 | 0.21 |  |  |  |  |  |  |  |
| 31 | NP_819099.2 | 0.56 | NP_819934.1 | 0.31 |  |  |  |  |  |  |  |
| 32 | NP_820817.1 | 0.41 | NP_819434.1 | 0.72 |  |  |  |  |  |  |  |
| 33 | NP_819725.1 | 0.28 |  |  |  |  |  |  |  |  |  |
| 34 | NP_820808.1 | 0.59 |  |  |  |  |  |  |  |  |  |
| 35 | NP_819521.1 | 0.61 |  |  |  |  |  |  |  |  |  |
| 36 | NP_820226.2 | 0.29 |  |  |  |  |  |  |  |  |  |
| 37 | NP_819159.1 | 0.33 |  |  |  |  |  |  |  |  |  |
| 38 | NP_820955.2 | 0.38 |  |  |  |  |  |  |  |  |  |
| 39 | NP_820941.1 | 0.31 |  |  |  |  |  |  |  |  |  |
| 40 | NP_819085.1 | 0.45 |  |  |  |  |  |  |  |  |  |
| 41 | YP_002333032.1 | 0.54 |  |  |  |  |  |  |  |  |  |
| 42 | NP_820794.1 | 0.33 |  |  |  |  |  |  |  |  |  |
| 43 | NP_820854.1 | 0.24 |  |  |  |  |  |  |  |  |  |
| 44 | NP_819961.1 | 0.42 |  |  |  |  |  |  |  |  |  |
| 45 | NP_819978.1 | 0.37 |  |  |  |  |  |  |  |  |  |
| 46 | NP_820789.1 | 0.67 |  |  |  |  |  |  |  |  |  |
| 47 | NP_819563.1 | 0.62 |  |  |  |  |  |  |  |  |  |
| 48 | NP_819929.1 | 0.30 |  |  |  |  |  |  |  |  |  |
| 49 | NP_820715.2 | 0.39 |  |  |  |  |  |  |  |  |  |
| 50 | NP_820094.2 | 0.66 |  |  |  |  |  |  |  |  |  |
| 51 | NP_819153.1 | 0.50 |  |  |  |  |  |  |  |  |  |
| 52 | NP_819517.1 | 0.44 |  |  |  |  |  |  |  |  |  |
| 53 | NP_820223.1 | 0.45 |  |  |  |  |  |  |  |  |  |
| 54 | NP_820583.1 | 0.63 |  |  |  |  |  |  |  |  |  |
| 55 | NP_819710.1 | 0.40 |  |  |  |  |  |  |  |  |  |
| 56 | NP_819101.2 | 0.41 |  |  |  |  |  |  |  |  |  |
| 57 | NP_819777.1 | 0.50 |  |  |  |  |  |  |  |  |  |
| 58 | NP_820080.2 | 0.86 |  |  |  |  |  |  |  |  |  |
| 59 | NP_820097.1 | 0.53 |  |  |  |  |  |  |  |  |  |
| 60 | NP_820328.1 | 0.29 |  |  |  |  |  |  |  |  |  |
| 61 | NP_819062.3 | 0.31 |  |  |  |  |  |  |  |  |  |
| 62 | NP_819806.2 | 0.30 |  |  |  |  |  |  |  |  |  |
| 63 | NP_820379.1 | 0.41 |  |  |  |  |  |  |  |  |  |
| 64 | NP_820689.1 | 0.49 |  |  |  |  |  |  |  |  |  |
| 65 | NP_819567.1 | 0.55 |  |  |  |  |  |  |  |  |  |
| 66 | NP_819243.1 | 0.66 |  |  |  |  |  |  |  |  |  |
| 67 | NP_819942.1 | 0.48 |  |  |  |  |  |  |  |  |  |
| 68 | YP_002332947.1 | 0.48 |  |  |  |  |  |  |  |  |  |
| 69 | NP_819564.2 | 0.35 |  |  |  |  |  |  |  |  |  |
| 70 | NP_821002.1 | 0.45 |  |  |  |  |  |  |  |  |  |
| 71 | NP_820216.1 | 0.34 |  |  |  |  |  |  |  |  |  |
| 72 | NP_819657.1 | 0.93 |  |  |  |  |  |  |  |  |  |
| 73 | NP_820361.1 | 0.80 |  |  |  |  |  |  |  |  |  |
| 74 | NP_820451.2 | 0.48 |  |  |  |  |  |  |  |  |  |
| 75 | NP_819577.2 | 0.64 |  |  |  |  |  |  |  |  |  |
| 76 | NP_821017.1 | 1.18 |  |  |  |  |  |  |  |  |  |
| 77 | NP_820984.2 | 0.43 |  |  |  |  |  |  |  |  |  |
| 78 | NP_821009.2 | 1.09 |  |  |  |  |  |  |  |  |  |
| 79 | NP_819206.1 | 0.76 |  |  |  |  |  |  |  |  |  |
| 80 | NP_820757.1 | 0.73 |  |  |  |  |  |  |  |  |  |
| 81 | NP_820138.2 | 0.35 |  |  |  |  |  |  |  |  |  |
| 82 | NP_819473.1 | 0.46 |  |  |  |  |  |  |  |  |  |
| 83 | NP_819417.1 | 0.31 |  |  |  |  |  |  |  |  |  |
| 84 | NP_819450.1 | 0.45 |  |  |  |  |  |  |  |  |  |
| 85 | NP_819755.1 | 0.55 |  |  |  |  |  |  |  |  |  |
| 86 | NP_819491.1 | 0.26 |  |  |  |  |  |  |  |  |  |
| 87 | NP_820169.1 | 0.57 |  |  |  |  |  |  |  |  |  |
| 88 | NP_819784.1 | 0.57 |  |  |  |  |  |  |  |  |  |
| 89 | NP_820562.1 | 0.37 |  |  |  |  |  |  |  |  |  |
| 90 | NP_820473.2 | 0.33 |  |  |  |  |  |  |  |  |  |
| 91 | NP_820355.1 | 0.41 |  |  |  |  |  |  |  |  |  |
| 92 | NP_819361.1 | 0.33 |  |  |  |  |  |  |  |  |  |
| 93 | NP_819774.1 | 0.46 |  |  |  |  |  |  |  |  |  |
| 94 | NP_819093.1 | 0.45 |  |  |  |  |  |  |  |  |  |
| 95 | NP_820133.1 | 0.62 |  |  |  |  |  |  |  |  |  |
| 96 | NP_820634.1 | 0.34 |  |  |  |  |  |  |  |  |  |
| 97 | NP_819649.1 | 0.76 |  |  |  |  |  |  |  |  |  |
| 98 | NP_820760.2 | 0.34 |  |  |  |  |  |  |  |  |  |
| 99 | NP_819589.1 | 0.59 |  |  |  |  |  |  |  |  |  |
| 100 | NP_819720.2 | 0.52 |  |  |  |  |  |  |  |  |  |
| 101 | NP_820790.1 | 0.66 |  |  |  |  |  |  |  |  |  |
| 102 | NP_820928.1 | 0.11 |  |  |  |  |  |  |  |  |  |
| 103 | AAO90589.2 | 0.28 |  |  |  |  |  |  |  |  |  |
| 104 | WP_010891173.1 | 0.58 |  |  |  |  |  |  |  |  |  |
| 105 | WP_012569658.1 | 0.38 |  |  |  |  |  |  |  |  |  |
| 106 | WP_078377999.1 | 0.58 |  |  |  |  |  |  |  |  |  |
| 107 | WP_230593246.1 | 0.55 |  |  |  |  |  |  |  |  |  |
| 108 | WP_011109640.1 | 0.91 |  |  |  |  |  |  |  |  |  |
| 109 | NP_820596.1 | 0.50 |  |  |  |  |  |  |  |  |  |

MRV: Manual Reverse Vaccinology; OPPA: Overall Prediction for the Protective Antigen; VFDB: Virulence Factor Database; ARV: Automated Reverse Vaccinology
