# Supplementary material for: Unveiling promising immunogenic targets in Coxiella burnetii through in silico analysis: paving the way for novel vaccine strategies
Source: BMC Infect Dis. 2023 Dec 21;23:902. doi: 10.1186/s12879-023-08904-7 (PMC10740251; doi:10.1186/s12879-023-08904-7)
Supplement: Supplementary file 3 — Supplementary Material 3 [file 12879_2023_8904_MOESM3_ESM.docx]

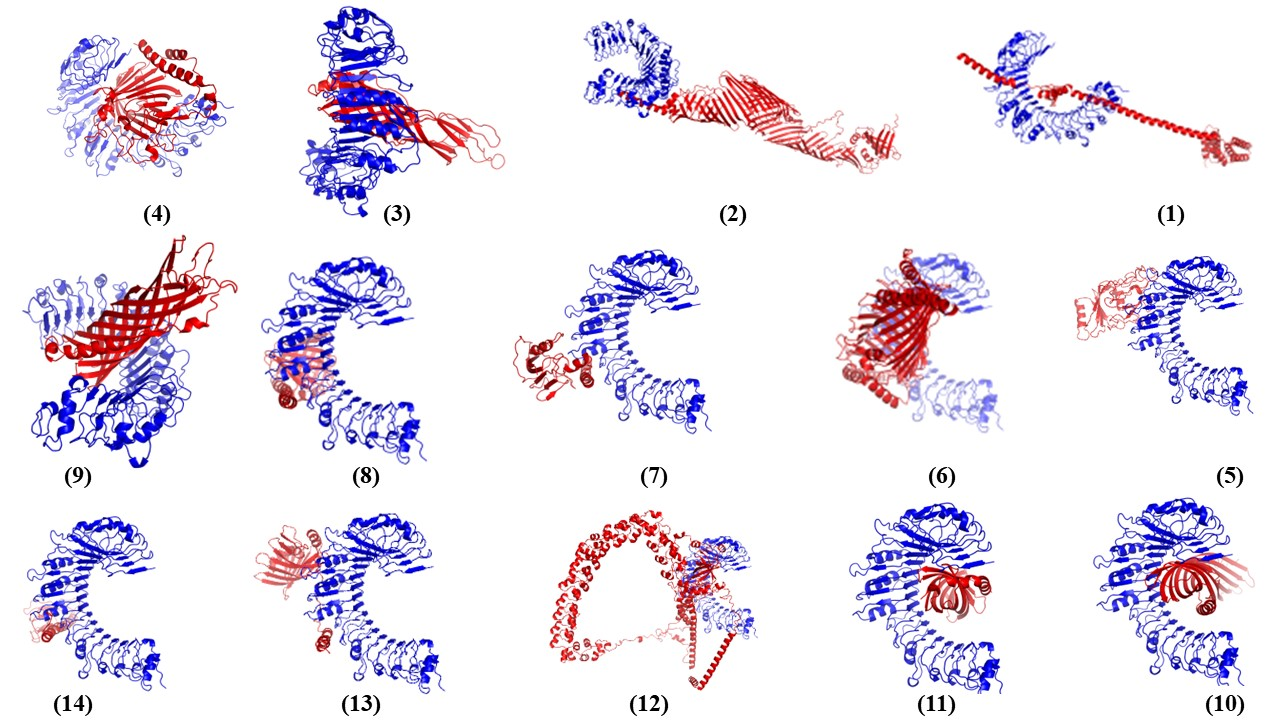


**Figure S1.** The Interaction of Toll-Like Receptor 1 with 14 Proteins: (1) Q83F57, (2) Q83EW1, (3) Q83EK8, (4) Q83E43, (5) Q83DJ4, (6) Q83D08, (7) Q83CL9, (8) P39917, (9) Q83BU0, (10) Q83BT8, (11) Q83BB2, (12) Q83B86, (13) Q83AQ2, (14) Q83A32.


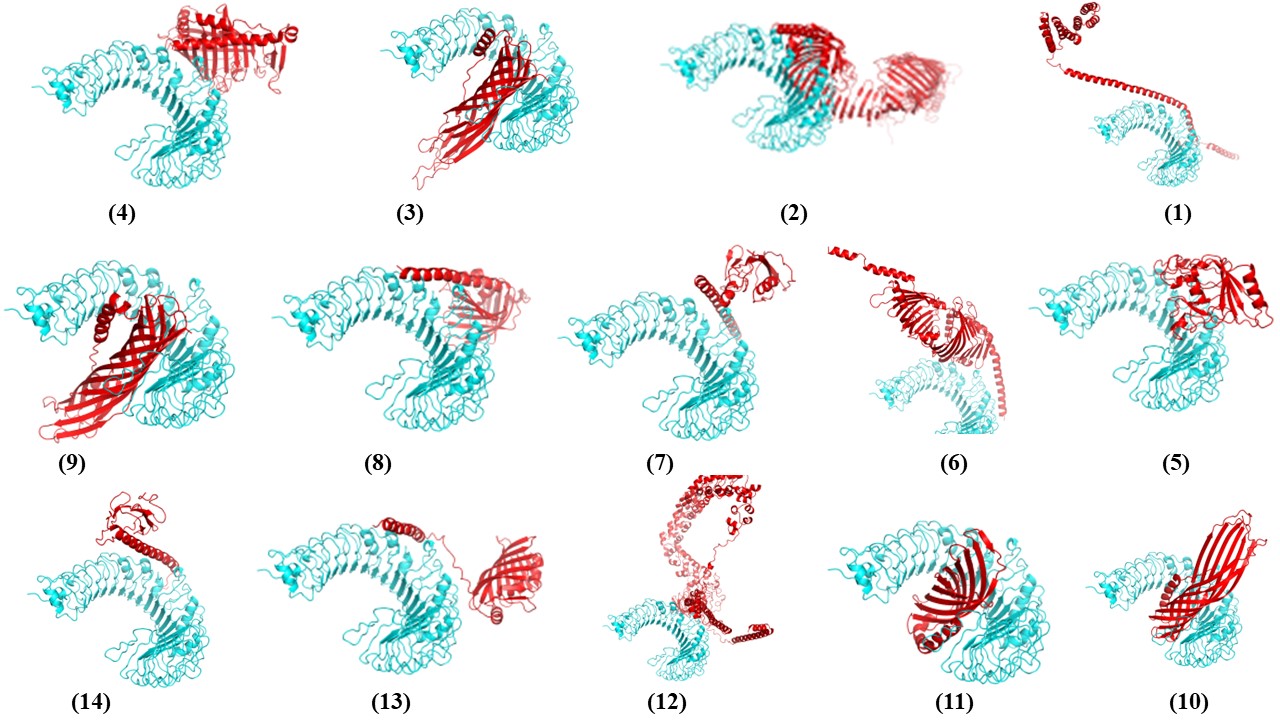


**Figure S2.** The Interaction of Toll-Like Receptor 2 with 14 Proteins: (1) Q83F57, (2) Q83EW1, (3) Q83EK8, (4) Q83E43, (5) Q83DJ4, (6) Q83D08, (7) Q83CL9, (8) P39917, (9) Q83BU0, (10) Q83BT8, (11) Q83BB2, (12) Q83B86, (13) Q83AQ2, (14) Q83A32.


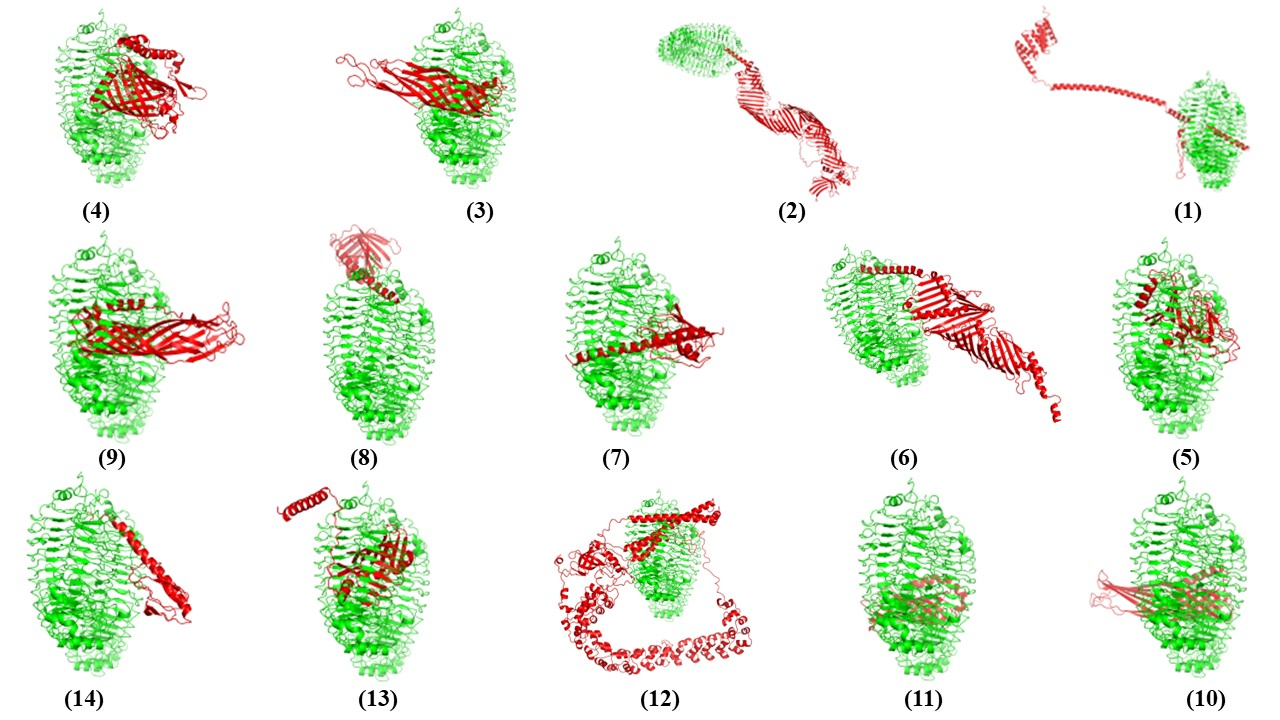


**Figure S3.** The Interaction of Toll-Like Receptor 4 with 14 Proteins: (1) Q83F57, (2) Q83EW1, (3) Q83EK8, (4) Q83E43, (5) Q83DJ4, (6) Q83D08, (7) Q83CL9, (8) P39917, (9) Q83BU0, (10) Q83BT8, (11) Q83BB2, (12) Q83B86, (13) Q83AQ2, (14) Q83A32.


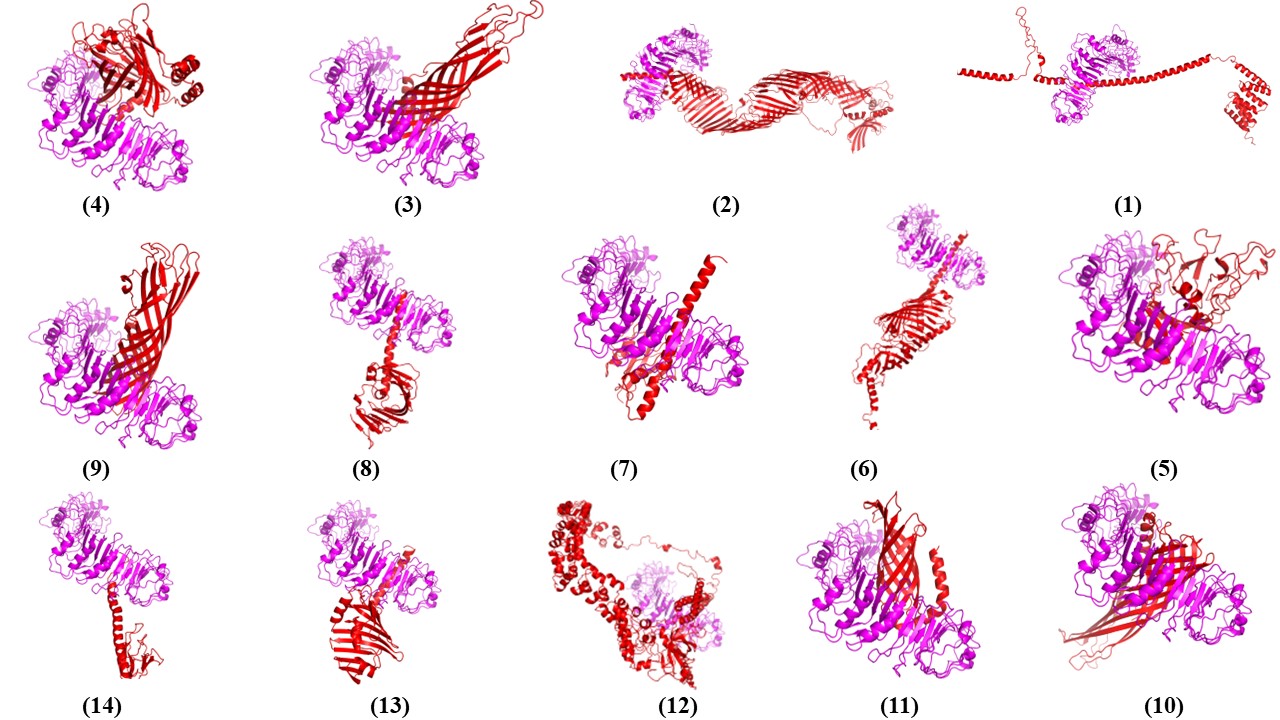


**Figure S4.** The Interaction of Toll-Like Receptor 6 with 14 Proteins: (1) Q83F57, (2) Q83EW1, (3) Q83EK8, (4) Q83E43, (5) Q83DJ4, (6) Q83D08, (7) Q83CL9, (8) P39917, (9) Q83BU0, (10) Q83BT8, (11) Q83BB2, (12) Q83B86, (13) Q83AQ2, (14) Q83A32.
