# Supplementary material for: Unveiling promising immunogenic targets in Coxiella burnetii through in silico analysis: paving the way for novel vaccine strategies
Source: BMC Infect Dis. 2023 Dec 21;23:902. doi: 10.1186/s12879-023-08904-7 (PMC10740251; doi:10.1186/s12879-023-08904-7)
Supplement: Supplementary file 4 — Supplementary Material 4 [file 12879_2023_8904_MOESM4_ESM.docx]

**Supplementary File 3.** Physicochemical and Immunological characteristics of selected proteins.

| Accession Number | Subcellular localization (servers ) | Ag/non-Ag | Antigenicity score | Allergen/non-allergen | Allergenicity Score (Hybrid Score) | Similarity to human proteome | TMH | Number of amino acids | Molecular weight (KDa) | No. of Linear B cell epitopes | B cell epitope ratio | MHC I binding sites | T MHC I binding sites’ ratio | MHC II binding sites | T MHC II binding sites’ ratio | Theoretical pI | estimated half time (E.coli) | Stable or not | instability index | aliphatic index | hydropathicity | Number of Predicted Discontinuous Epitope(s) | Predicted Functional Class | Functional Class Score | Adhesin probability |
| --- | --- | --- | --- | --- | --- | --- | --- | --- | --- | --- | --- | --- | --- | --- | --- | --- | --- | --- | --- | --- | --- | --- | --- | --- | --- |
| NP_819144.2 | OuterMembrane (SOSUIGramN) | Ag | 0.68 | Non-allergen | 0.08 | No | 0 | 305 | 34.26 | 7 | 0.544 | 50 | 0.163934 | 75 | 0.245902 | 6.46 | >10 hours | Unstable | 46.42 | 83.25 | -0.746 | 4 | Unknown | 0 | 0.654 |
| NP_819243.1 | OuterMembrane (GenBank) | Ag | 0.66 | Non-allergen | 0.14 | No | 1 | 929 | 101.93 | 19 | 0.37 | 254 | 0.273412 | 225 | 0.242196 | 9.19 | >10 hours | stable | 28.65 | 101.81 | -0.071 | 4 | Virulence factors | 19.86359 | 0.514 |
| NP_819354.2 | Extracellular (SOSUIGramN) | Ag | 0.63 | Non-allergen | -0.38 | No | 1 | 252 | 26.77 | 10 | 0.428 | 110 | 0.436508 | 50 | 0.198413 | 8.44 | >10 hours | Stable | 17.64 | 89.01 | 0.199 | 5 | Virulence factors | 2.690361 | 0.885 |
| NP_819523.1 | OuterMembrane (PSORTb) | Ag | 0.58 | Non-allergen | 0.12 | No | 0 | 344 | 39.96 | 12 | 0.549 | 151 | 0.438953 | 75 | 0.218023 | 9.37 | >10 hours | Unstable | 40.52 | 79.62 | -0.369 | 7 | Cellular process | 0.11399302 | 0.631 |
| NP_819755.1 | OuterMembrane (CELLO2GO) | Ag | 0.55 | Non-allergen | 0.12 | No | 1 | 308 | 33.97 | 2 | 0.116 | 96 | 0.311688 | 75 | 0.243506 | 9.22 | >10 hours | Unstable | 45.5 | 96.56 | -0.13 | 6 | Virulence factors | 1.0796458 | 0.541 |
| NP_819762.1 | Extracellular (SOSUIGramN) | Ag | 0.75 | Non-allergen | 0.24 | No | 1 | 217 | 24 | 10 | 0.755 | 71 | 0.327189 | 50 | 0.230415 | 10 | >10 hours | stable | 30.2 | 74.15 | -0.39 | 3 | Virulence factors | -0.50187 | 0.594 |
| NP_819951.2 | Extracellular (PSORTb) | Ag | 0.6 | Non-allergen | 0.12 | No | 1 | 508 | 55.63 | 17 | 0.537 | 142 | 0.279528 | 125 | 0.246063 | 10.05 | >10 hours | stable | 20.91 | 99.23 | -0.176 | 7 | Virulence factors | 1.560984 | 0.785 |
| NP_820094.2 | Extracellular (LocTree3) | Ag | 0.66 | Non-allergen | 0.19 | No | 1 | 187 | 20.25 | 8 | 0.55 | 32 | 0.171123 | 50 | 0.26738 | 6.89 | >10 hours | stable | 35.88 | 93.9 | -0.047 | 2 | Virulence factors | 0.849267 | 0.774 |
| NP_820185.1 | OuterMembrane (CELLO2GO) | Ag | 0.55 | Non-allergen | -0.39 | No | 1 | 211 | 23.8 | 10 | 0.905 | 73 | 0.345972 | 50 | 0.236967 | 10.04 | >10 hours | stable | 33.4 | 95.17 | -0.163 | 4 | Cellular process | 0.455085 | 0.566 |
| NP_820396.2 | OuterMembrane (SOSUIGramN) | Ag | 0.53 | Non-allergen | 0.3 | No | 1 | 273 | 30.23 | 9 | 0.505 | 96 | 0.351648 | 75 | 0.274725 | 8.51 | >10 hours | Stable | 33.77 | 87.55 | -0.038 | 3 | Cellular process | 6.045218 | 0.641 |
| NP_820398.1 | OuterMembrane (SOSUIGramN) | Ag | 0.65 | Non-allergen | 0.16 | No | 1 | 263 | 29.04 | 6 | 0.433 | 112 | 0.425856 | 75 | 0.285171 | 9.35 | >10 hours | Stable | 37.4 | 95.63 | 0.032 | 4 | Metabolism | 0.825746 | 0.825 |
| NP_820583.1 | OuterMembrane (SOSUIGramN) | Ag | 0.63 | Non-allergen | 0.3 | No | 0 | 181 | 19.45 | 4 | 0.243 | 55 | 0.303867 | 50 | 0.276243 | 6.71 | >10 hours | stable | 19.37 | 92.04 | 0.076 | 4 | Cellular process | 1.025492 | 0.782 |
| NP_820596.1 | Extracellular (SOSUIGramN) | Ag | 0.5 | Non-allergen | 0.3 | No | 0 | 139 | 16.19 | 3 | 0.208 | 25 | 0.179856 | 25 | 0.179856 | 9.03 | >10 hours | Stable | 36.74 | 68.85 | -0.757 | 3 | Unknown | 0 | 0.631 |
| NP_820609.2 | Extracellular (PSORTb) | Ag | 0.58 | Non-allergen | 0.46 | No | 1 | 1039 | 106.51 | 15 | 0.704 | 177 | 0.170356 | 250 | 0.240616 | 9.14 | >10 hours | stable | 30.96 | 75.94 | -0.099 | 3 | Metabolism | 30.00465 | 0.711 |
| NP_820793.1 | OuterMembrane (LocTree3) | Ag | 0.61 | Non-allergen | 0.22 | No | 1 | 237 | 26.25 | 3 | 0.185 | 109 | 0.459916 | 50 | 0.21097 | 8.85 | >10 hours | stable | 32.85 | 95.49 | 0.245 | 4 | Metabolism | 2.070274 | 0.719 |
| NP_820808.1 | Extracellular (SOSUIGramN) | Ag | 0.59 | Non-allergen | -0.38 | No | 0 | 209 | 24.02 | 5 | 0.301 | 70 | 0.334928 | 50 | 0.239234 | 9.66 | >10 hours | Unstable | 52.27 | 89.62 | -0.311 | 5 | Cellular process | 0.870486 | 0.584 |
| NP_820832.2 | OuterMembrane (CELLO2GO) | Ag | 0.53 | Non-allergen | 0.17 | No | 0 | 115 | 13.58 | 5 | 0.382 | 49 | 0.426087 | 25 | 0.217391 | 9.11 | >10 hours | Unstable | 41.83 | 81.48 | -0.211 | 4 | Metabolism | 0.242029 | 0.583 |
| NP_821009.2 | OuterMembrane (CELLO2GO) | Ag | 1.09 | Non-allergen | 0.4 | No | 1 | 58 | 6.33 | 2 | 0.517 | 21 | 0.362069 | 200 | 3.448276 | 10.87 | >10 hours | Unstable | 47.26 | 68.97 | 0.429 | 2 | Unknown | 0 | 0.58 |
| NP_821049.1 | Extracellular (LocTree3) | Ag | 0.7 | Non-allergen | 0.49 | No | 1 | 134 | 14.26 | 6 | 0.597 | 30 | 0.223881 | 25 | 0.186567 | 8.69 | >10 hours | stable | 13.78 | 79.4 | -0.184 | 2 | Metabolism | 0.684798 | 0.702 |
| NP_821052.2 | Extracellular (SOSUIGramN) | Ag | 0.76 | Non-allergen | 0.14 | No | 1 | 255 | 28.12 | 5 | 0.18 | 84 | 0.329412 | 50 | 0.196078 | 10.01 | >10 hours | Stable | 37.29 | 106.43 | 0.162 | 4 | Cellular process | 0.94665 | 0.535 |
| WP_010891173.1 | Extracellular (LocTree3) | Ag | 0.58 | Non-allergen | 0.5 | No | 0 | 171 | 19.06 | 5 | 0.245 | 51 | 0.298246 | 50 | 0.292398 | 5.62 | >10 hours | stable | 36.75 | 76.49 | -0.209 | 1 | Metabolism | -0.01537 | 0.774 |
| YP_002332945.1 | OuterMembrane (SOSUIGramN) | Ag | 1.06 | Non-allergen | 0.32 | No | 0 | 113 | 12.48 | 4 | 0.769 | 29 | 0.256637 | 25 | 0.221239 | 9.91 | >10 hours | stable | 31.4 | 66.46 | -0.587 | 1 | Cellular process | 0.043977884 | 0.648 |
