# Supplementary material for: Unveiling promising immunogenic targets in Coxiella burnetii through in silico analysis: paving the way for novel vaccine strategies
Source: BMC Infect Dis. 2023 Dec 21;23:902. doi: 10.1186/s12879-023-08904-7 (PMC10740251; doi:10.1186/s12879-023-08904-7)
Supplement: Supplementary file 5 — Supplementary Material 5 [file 12879_2023_8904_MOESM5_ESM.docx]

**Supplementary Table S2**. Linear and conformational B-cell epitopes of 22 putative immunogenic targets of *C. burnetii,* determined by BepiPred (threshold ≥0.6) and Ellipro (threshold ≥0.8), respectively.

| **Accession number (UniProt ID)** | **Linear B cell epitopes^*^** | **Start-End** | **Antigenicity** | **Conformational B cell epitopes^£^** | **Score** | **Color in 3D Modeling** |
| --- | --- | --- | --- | --- | --- | --- |
| **NP_821052.2 (Q83A29)** | **NFSLQIP** | 234-240 | 2.37 | A:K2, A:Q3, A:T4, A:L5, A:L6, A:R7, A:Y8, A:F9, A:F12, A:F13, A:L16, A:E19 | 0.924 | Red |
|  | **SIQWKALNPGM** | 26-36 | 1.34 | A:S149, A:P150, A:E151 | 0.907 | Green |
|  | **NNFSLQIPSLR** | 233-243 | 1.60 | A:A74, A:S76, A:L77, A:Q78, A:Q79, A:T80, A:A81 | 0.816 | Blue |
|  | **IPQLRGGSA** | 168-176 | 1.00 | A:A44, A:F45, A:S46, A:S47, A:E48, A:S49, A:R50, A:P51, A:G52, A:F54, A:L198 | 0.808 | Yellow |
|  | **QNYRYSPE** | 144-151 | 1.17 |  |  |  |
| **NP_821049.1 (Q83A32)** | **QGGTHDAGE** | 59-67 | 2.01 | A:M1, A:S2, A:Y3, A:I4, A:K5, A:R6 | 0.974 | Red |
|  | **FDDQGGTHDAGEVEANGQTKITAHLDQASGF** | 56-86 | 1.14 |  |  |  |
|  | **NVAGKREVVCSYKSGSHPNG** | 89-108 | 1.02 |  |  |  |
|  | **TKKISGGH** | 124-131 | 1.26 | A:D7, A:H8, A:T9, A:A10, A:L11, A:R12, A:D13 | 0.901 | Green |
|  | **KRDHTA** | 5-10 | 1.38 |  |  |  |
|  | **KNNYNQ** | 44-49 | 1.11 |  |  |  |
| **NP_819762.1 (Q83DJ4)** | **NPRYRGACSSTSGNGGKVRRIGDINFSYYPSSTFSSSGGKLQR** | 148-190 | 1.46 | A:D33, A:R34, A:Y35, A:H64, A:D65, A:P66, A:K67, A:H68, A:T70, A:L71, A:L74, A:I77, A:I78, A:G79, A:K80, A:E81, A:Q82, A:V83, A:P84, A:P85 | 0.861 | Red |
|  | **DKIIGKEQVPPSAQKLSIKYYPASALNGNGGKIAEANGI** | 75-113 | 0.75 |  |  |  |
|  | **APATRFSNAHYYGQKFSANNNS** | 121-142 | 0.61 |  |  |  |
|  | **QNSFQGKGGQLKNF** | 201-214 | 1.34 |  |  |  |
|  | **TVTKRVDRYG** | 27-36 | 0.87 | A:F199, A:P200, A:Q201, A:N202, A:S203, A:F204, A:Q205, A:G206 | 0.847 | Green |
|  | **FQGKGGQLK** | 204-212 | 2.08 |  |  |  |
|  | **TFSSSGG** | 180-186 | 1.25 |  |  |  |
|  | **STSGNGG** | 157-163 | 3.90 | A:A121, A:P122, A:A123, A:T124, A:R125, A:F126, A:S127, A:N128, A:A129, A:H130, A:Y131, A:I147, A:N148, A:P149, A:R150, A:Y151, A:R152, A:C155 | 0.829 | Blue |
|  | **GQKFSA** | 133-138 | 1.09 |  |  |  |
|  | **INPRYRG** | 147-153 | 0.73 |  |  |  |
| **NP_820808.1 (Q83AQ2)** | **SQYTTVGSVD** | 179-188 | 0.70 | A:M1, A:S2, A:L3, A:I4, A:S5, A:N6, A:N7, A:E8, A:E9, A:R10, A:R13 | 0.968 | Red |
|  | **QQQLGWQL** | 131-138 | 1.26 | A:S11, A:L12, A:V14, A:R15, A:Y16, A:C17, A:I18, A:A19, A:I20, A:A21, A:L22, A:S23, A:A24, A:L25, A:L26, A:I27, A:S28, A:G29, A:C30 | 0.88 | Green |
|  | **SNNEERSL** | 5-12 | 0.93 | A:S116, A:P117, A:R118, A:Q119 | 0.869 | Oranges |
|  | **RQHYTAS** | 118-124 | 0.95 | A:P72, A:G73, A:S199 | 0.811 | Blue |
|  | **MSLISNNEERSLRVRYCIAIALSALLISGC** | 1-30 | 0.87 | A:Q46, A:T47, A:A49, A:Q50, A:Y53 | 0.81 | Yellow |
| **NP_820094.2 (Q83CL9)** | **ETNMNI** | 19-24 | 1.34 | A:M1, A:A2, A:A3, A:T4, A:Q5, A:K6, A:Q7, A:T8, A:I9, A:L10, A:I11, A:V12, A:K13, A:A14, A:I16, A:I17 | 0.923 | Red |
|  | **ISGATFAQGKGISPELEGTL** | 39-58 | 0.84 |  |  |  |
|  | **YVKNNTRED** | 62-70 | 0.67 |  |  |  |
|  | **KVNGQELK** | 85-92 | 2.64 |  |  |  |
|  | **DQEGGSCA** | 110-117 | 2.54 | A:G41, A:A42, A:T43, A:A45, A:Q46, A:G47, A:K48, A:G49, A:I50 | 0.882 | Green |
|  | **ATLNLSGKLEPGTSSDYKQCEMRSKTYGNQKTTL** | 138-171 | 1.04 |  |  |  |
|  | **KLEPGTSSD** | 145-153 | 2.16 |  |  |  |
|  | **MRSKTYGNQ** | 159-167 | 0.65 |  |  |  |
| **NP_820583.1 (Q83BB2)** | **YYSNSLEANSDAK** | 73-85 | 1.24 | A:G130, A:P131, A:D132 | 0.91 | Red |
|  | **DYRYLGWTDGNGSQNLIEM** | 157-175 | 0.69 | A:L78, A:E79, A:A80, A:N81, A:S82, A:D83, A:N122, A:N123, A:G124, A:V125, A:L126, A:L127, A:N128, A:R129 | 0.899 | Green |
|  | **NSLEAN** | 76-81 | 2.05 | A:P15, A:P16, A:A19 | 0.893 | Blue |
|  | **LEANSD** | 78-83 | 1.05 | A:V148, A:S149, A:P150, A:R151, A:Q180, A:F181 | 0.849 | Yellow |
| **NP_819243.1 (Q83EW1)** | **LRSGPNH** | 476-473 | 0.87 | A:E853, A:S854, A:Q855, A:T856, A:T857, A:I858, A:A859, A:A860, A:L861, A:G862, A:G863, A:P864, A:T865, A:G866, A:P867, A:T868, A:Q869, A:S879, A:P880, A:R881, A:I882, A:T891, A:T892, A:S893, A:I894, A:V896, A:I897, A:Q898, A:I899, A:R900, A:Y901, A:L902, A:I903, A:S904, A:E905, A:N906, A:W907, A:A908, A:I909, A:Q910, A:T911, A:E912, A:T913, A:S914, A:S915, A:L916, A:G917, A:S918, A:G919, A:V920, A:D921, A:V922, A:L923, A:Y924, A:S925, A:I926, A:E927, A:R928, A:N929 | 0.936 | Red |
|  | **DIVDRNI** | 621-627 | 1.19 |  |  |  |
|  | **ETSSLGSGV** | 912-920 | 1.29 |  |  |  |
|  | **DFPQLQVSLT** | 543-552 | 0.67 |  |  |  |
|  | **ALYSSDPNLTQ** | 784-794 | 0.95 |  |  |  |
|  | **IRYSRGITTSINV** | 884-896 | 1.00 | A:M1, A:I2, A:K3, A:W4, A:V5, A:T6, A:G7, A:I8, A:L9, A:I10, A:S11, A:L12, A:A13, A:I14, A:L15, A:V16, A:G17, A:F18, A:L19, A:T20, A:I21, A:L22, A:M23, A:M24, A:T25, A:P26, A:A27, A:G28, A:L29, A:N30, A:I31, A:R34, A:Y46, A:S47, A:S48, A:A49, A:S50, A:G51, A:I52, A:P53, A:T54, A:G55, A:P56, A:I57, A:A58, A:Q77, A:K79, A:R81, A:L82, A:F83, A:Y84, A:L85, A:L86, A:R87, A:G88, A:V89, A:H91, A:T93, A:Q94, A:H122, A:R123, A:Q148, A:N156 | 0.898 | Green |
|  | **TLNIGGGKTSAGGVVD** | 824-839 | 1.74 |  |  |  |
|  | **GHSANANTPANVNLLVD** | 804-820 | 0.89 |  |  |  |
|  | **LHSKGTRDNYQLSLQTKSKDI** | 173-193 | 1.41 |  |  |  |
|  | **AQHVHTPVHRLSALKIDASGQPHAH** | 373-397 | 0.6807 |  |  |  |
|  | **VYFDPIFKWDINLDMVDLNLRKFNRNWPQQLTVQLKTKGEHKIGESPDF** | 226-274 | 1.46 | A:K755, A:T756, A:S757, A:P758, A:I759, A:V760, A:P761, A:Q762, A:L763, A:G764, A:G765, A:P766, A:T767, A:V768, A:S788, A:D789, A:P790, A:N791, A:L792, A:S806, A:A807, A:N808, A:A809, A:N810, A:T811 | 0.859 | Blue |
|  | **IVLPLHTEETKSRLPFDISIHRGLLKNLSIKYPAYDPVHFKTILLNQIEFNHALNGDIQAQITQPYPVNV** | 102-171 | 0.75 |  |  |  |
|  | **IKIVLPLHTEE** | 100-110 | 0.77 |  |  |  |
|  | **SPFCLRSGP** | 463-471 | 1.00 |  |  |  |
|  | **NIDTSFNQA** | 358-366 | 0.62 |  |  |  |
|  | **ANANTPA** | 807-813 | 0.71 | A:N152, A:H153, A:A154, A:K176, A:G177, A:T178, A:R179, A:D180, A:N181, A:Q183, A:K200, A:G201, A:T202, A:R203, A:Q204, A:W205, A:E207, A:Y227, A:F228, A:D229, A:P230, A:I231, A:K233, A:E265, A:K267, A:I268, A:G269, A:E270, A:S271, A:Q295, A:Q296 | 0.839 | Yellow |
|  | **TTSINVIQIRYLISENWAIQTETSSLGSGVDVLYSIERN** | 891-929 | 0.87 |  |  |  |
|  | **EHKIGES** | 265-271 | 2.04 |  |  |  |
|  | **YFDPIFK** | 227-233 | 0.78 |  |  |  |
| **NP_819755.1 (Q83DK1)** | **VSGIST** | 48-53 | 0.65 | A:Y299, A:P300, A:P301, A:P302, A:P303, A:G304, A:P305, A:G306, A:E307 | 0.98 | Red |
|  |  |  |  | A:H279, A:L280, A:Q282, A:L283, A:S284, A:I285, A:E286, A:L287, A:Q288, A:H289, A:N290, A:P291, A:S292, A:M293, A:L294, A:V295, A:R296, A:G297, A:K298 | 0.893 | Green |
|  |  |  |  | A:V184, A:T185, A:K186, A:T187, A:L188, A:S189, A:D190, A:N191, A:S192, A:E193, A:N194, A:I195, A:D196, A:A197, A:H200 | 0.887 | Blue |
|  | **HLQQLSIELQHNPSMLVRGKYPPPPGPGER** | 279-308 | 0.60 | A:M1, A:D2, A:T3, A:K4, A:V5, A:N6, A:L9 | 0.858 | Yellow |
|  |  |  |  | A:S175, A:A176, A:L178, A:V179, A:N180, A:Q182, A:K183 | 0.855 | Orange |
|  |  |  |  | A:D167, A:E168, A:R172 | 0.823 | Cyan |
| **WP_010891173.1 (H7C7E6)** | **GDGSYTD** | 104-110 | 2.26 | A:N122, A:C123, A:N124, A:N125, A:Q126, A:A127, A:V128, A:N130, A:S131, A:E166, A:P167 | 0.816 | Red |
|  | **RDIKETQP** | 50-57 | 1.1158 |  |  |  |
|  | **QKGDGSYTDS** | 102-11 | 1.96 |  |  |  |
|  | **QDEVNGAFCSR** | 65-75 | 0.84 |  |  |  |
|  | **KTATGE** | 36-41 | 1.9597 |  |  |  |
| **NP_820596.1 (Q83B99)** | **QDEVNGAFCSR** | 65-75 | 0.84 | A:F21, A:S22, A:S23, A:K24, A:K25, A:A26, A:E27, A:K28, A:K30, A:K31 | 0.918 | Red |
|  | **QKGDGSYTDS** | 102-111 | 1.96 | A:K38, A:A41, A:E42 | 0.906 | Green |
|  | **RDIKETQP** | 50-57 | 1.11 | A:A117, A:S118, A:T119, A:P120, A:E121 | 0.822 | Blue |
| **YP_002332945.1 (B5QS73)** | **SPQKQN** | 51-56 | 0.81 | A:D37, A:S38, A:N39, A:G40, A:G41, A:Q42, A:L43, A:T89 | 0.862 | Red |
|  | **KNATEGSFNFRMQYTDSNGGQLKTQSAQISPQKQNKFTV** | 22-60 | 1.45 |  |  |  |
|  | **KEGYTQWRLPNCKSTGGALEVKVFAKGSYKQITCR** | 75-109 | 1.00 |  |  |  |
|  | **DSNGGQL** | 37-43 | 2.53 |  |  |  |
| **NP_819951.2 (Q83D08)** | **WSHSAVTKNNSKNTADNNKKMTA** | 138-160 | 0.92 | A:M1, A:T2, A:K3, A:G4, A:P5, A:F6, A:M7, A:R8 | 0.987 | Red |
|  | **LNFQQSN** | 233-239 | 1.04 |  |  |  |
|  | **THRSAPRLKN** | 383-392 | 0.78 | A:L483, A:E486, A:I487, A:E488, A:K489, A:K490, A:V491, A:K492, A:Q493, A:Y494, A:T495, A:G496, A:G497, A:I498, A:K499, A:K500, A:L501, A:N502, A:L503, A:N504, A:N505, A:F506, A:F507, A:H508 | 0.932 | Green |
|  | **NQSKNTF** | 217-222 | 1.62 |  |  |  |
|  | **ADLNFQQSNYRL** | 231-242 | 1.49 |  |  |  |
|  | **KFNGTLLR** | 246-253 | 0.66 | A:T9, A:F10, A:I11, A:K12, A:I13, A:L14, A:V15, A:S16, A:V17, A:V18, A:A19, A:A20, A:L21, A:L22, A:L23, A:V24, A:S25, A:T26, A:I27, A:V30, A:V31, A:K34, A:L35, A:V36 | 0.894 | Blue |
|  | **THRSAPRLKNS** | 383-393 | 0.91 |  |  |  |
|  | **LASNQSKNTFNATLTADADLNFQQSNYRLSNIKFNGTLLRPTLPPVPVNI** | 214-263 | 0.96 |  |  |  |
|  | **NKNGKDNWAQWSHSAVTKNNSKNTADNNKKMTAPVTELK** | 128-166 | 0.73 | A:A380, A:L381, A:T383, A:H384, A:R385, A:S386, A:A387, A:P388, A:R389 | 0.883 | Yellow |
|  | **ILGININEFLAQANALLTHRSAPRLKNSKTT** | 366-396 | 0.73 |  |  |  |
|  | **TSGNSKEDFLSHLNGSGKF** | 341-359 | 0.63 |  |  |  |
|  | **EKKVKQYTGGIKKLNLN** | 488-504 | 0.97 | A:A152, A:D153, A:K156, A:K157, A:M158 | 0.86 | Cyan |
|  | **SPTIRPDFSA** | 468-477 | 1.94 |  |  |  |
|  | **VNPNDYKDR** | 36-44 | 1.17 | A:L390, A:K391, A:N392, A:S393, A:K394 | 0.841 | Oranges |
|  | **KLTNMQLN** | 284-291 | 1.20 |  |  |  |
|  | **WSFIPW** | 65-70 | 3.69 | A:K449, A:S450, A:G451, A:A452, A:P453 | 0.838 | Purple |
|  | **LNQQNI** | 271-276 | 0.85 |  |  |  |
| **NP_820185.1 (P39917)** | **DVDLSQATQQPLAQKTNINP** | 96-115 | 0.70 | A:M1, A:N2, A:T3, A:I4, A:K5, A:I6, A:L7, A:I8, A:G9, A:L10, A:L11, A:G12, A:I13, A:F14, A:L15 | 0.95 | Red |
|  | **SAQSDATTQL** | 23-32 | 1.25 |  |  |  |
|  | **QIKVNAPLSS** | 186-195 | 1.11 | A:D52, A:G53, A:Q54, A:D55, A:R56, A:D177 | 0.885 | Green |
|  | **SGSVKDLKQ** | 120-128 | 1.04 |  |  |  |
|  | **DGQDRVI** | 52-58 | 0.68 |  |  |  |
|  | **PVSLPTTQTDLTV** | 61-73 | 0.76 | A:P138, A:D139, A:K164 | 0.848 | Yellow |
|  | **SNDNSASSSQKPSSGDTSNTNNIQLQD** | 149-175 | 1.68 |  |  |  |
|  | **QVQERNLELLNNQQRSFYRDLDQRITQLKNLNSNNSDSSNDNSASSSQKPSSGDTSNTNNIQLQDSNTYRQALDLLTKKQYDK** | 111-193 | 0.97 | A:D98, A:L99, A:S100, A:S203, A:G205, A:I206, A:D207, A:V208, A:V209, A:K210 | 0.817 | Blue |
|  | **KQHPEST** | 277-283 | 1.51 |  |  |  |
|  | **QKDRKN** | 225-230 | 1.28 |  |  |  |
| **NP_819144.2 (Q83F57)** | **ISAQPQPTKTTVSPSETPETA** | 36-56 | 0.83 | A:M1, A:R2, A:L3, A:I4, A:K5, A:M6, A:K7 | 0.935 | Red |
|  | **PVSLPTTQTDLTV** | 61-73 | 0.76 | A:Q41, A:P42, A:T43, A:K44, A:T45, A:T46, A:V47, A:S48, A:P49, A:S50, A:E51, A:T52, A:P53, A:E54, A:T55, A:A56, A:I57, A:P58, A:T59, A:A60, A:P61, A:V62 | 0.926 | Green |
|  | **SNDNSASSSQKPSSGDTSNTNNIQLQD** | 149-175 | 1.68 |  |  |  |
|  | **QVQERNLELLNNQQRSFYRDLDQRITQLKNLNSNNSDSSNDNSASSSQKPSSGDTSNTNNIQLQDSNTYRQALDLLTKKQYDK** | 111-193 | 0.97 |  |  |  |
|  | **KQHPEST** | 277-283 | 1.51 | A:V298, A:D299, A:S300, A:A301, A:T302, A:T303, A:T304, A:P305 | 0.899 | Blue |
|  | **QKDRKN** | 225-230 | 1.28 | A:I8, A:K9, A:T10, A:L11, A:C12, A:V13, A:S14, A:S15, A:A16, A:L17, A:A18, A:A19, A:L20, A:M21, A:L22 | 0.866 | Yellow |
|  | **EEVDSATTTP** | 296-305 | 1.23 |  |  |  |
| **NP_820793.1 (Q83AR6)** | **HVNGYGRASAGAIQKLPSPH** | 204-223 | 0.81 | A:N21, A:A22, A:A23, A:A24, A:G25, A:G26, A:I27, A:E28, A:V29, A:P30, A:H31 | 0.925 | Red |
|  | **GIEVPHYIPIP** | 26-36 | 0.95 | A:L59, A:P60, A:Y61, A:Q62, A:T63, A:Y64, A:V65, A:A66, A:A67, A:L68, A:Q69, A:G70, A:D71, A:G72, A:P73, A:P74, A:F75, A:F78 | 0.9 | Green |
|  | **NAAAGGIEVPHYI** | 21-33 | 0.8352 | A:A213, A:G214, A:A215, A:I216, A:Q217 | 0.852 | Blue |
|  |  |  |  | A:S40, A:L235, A:A236, A:I237 | 0.82 | Yellow |
| **NP_820398.1 (Q83BT8)** | **SRQGNNDSVTFVGSGFLFL** | 111-129 | 0.74 | A:E57, A:S58, A:I59, A:V60, A:G61, A:I62, A:G63, A:P64, A:V65, A:D66, A:M67, A:P68, A:T70, A:A71, A:I72, A:R73, A:N74 | 0.897 | Red |
|  | **MASSLQTSVQQK** | 177-188 | 0.89 |  |  |  |
|  | **QGNSRQGNNDSVTFVGSGFLFLLQNEFRI** | 108-136 | 1.12 | A:G123, A:S124, A:G125, A:M177, A:A178, A:S179, A:S180, A:L181, A:Q182 | 0.892 | Green |
|  | **KLTVSQWMASSLQTSVQQKLNKNL** | 170-193 | 0.61 | A:F17, A:S20, A:L21, A:I22, A:T23, A:I24, A:N25, A:A26, A:F27, A:A28, A:G29, A:G30, A:L206, A:G207, A:R208, A:G209 | 0.853 | Blue |
|  | **INAFAGGPEIPSFNPWTIS** | 24-42 | 0.70 |  |  |  |
|  | **GIRYHLERSYL** | 91-101 | 0.61 | A:P38, A:W39, A:Y94, A:L96, A:E97 | 0.853 | Yellow |
| **NP_820396.2 (Q83BU0)** | **TTSASISQQHQTH** | 158-197 | 1.00 | A:P44, A:E45, A:S46, A:P47, A:W48, A:Y99, A:F101, A:I102, A:R103, A:P104, A:Y105, A:N272 | 0.822 | Red |
|  | **TLNTANNISVD** | 232-242 | 0.70 |  |  |  |
|  | **PDVYTQEAK** | 248-256 | 1.21 |  |  |  |
|  | **YGNHTLNTANNISVDPVVAPPDVYTQEA** | 228-255 | 0.66 | A:S28, A:L29, A:I30, A:G31, A:S32, A:G33, A:S34, A:A35, A:L36, A:A37, A:G38, A:G39, A:Y40 | 0.806 | Green |
|  | **SNSRKAQFNGSVSLGAGSTTVPVIDTTLRL** | 115-144 | 0.81 |  |  |  |
|  | **VEDFAVLQATTSASISQQHQTHHG** | 176-199 | 0.64 |  |  |  |
|  | **GGYEPL** | 38-43 | 0.70 | A:A184, A:T185, A:T186, A:S187, A:A188, A:S189, A:S240, A:V241, A:D242, A:P243, A:V244, A:V245 | 0.804 | Blue |
|  | **SLIGSGSALAG** | 28-38 | 0.63 |  |  |  |
|  | **LGAGST** | 128-133 | 1.01 |  |  |  |
| **NP_820832.2 (Q83AM8)** | **QKENTDL** | 44-50 | 0.95 | A:M1, A:F2, A:M3, A:P4, A:W5, A:L6, A:R7, A:F8, A:L9 | 0.929 | Red |
|  | **KINYKYRV** | 94-101 | 1.48 |  |  |  |
|  | **RQKRYKAAIE** | 78-87 | 0.74 | A:L113, A:L114, A:H115 | 0.89 | Green |
|  | **RFLIIFLMGF** | 7-16 | 1.84 |  |  |  |
|  | **MFMPWLRFL** | 1-9 | 1.61 | A:Y25, A:S26, A:P27 | 0.835 | Blue |
| **NP_821009.2 (Q83A72)** | **RAGGNP** | 20-25 | 1.01 | A:M1, A:M2, A:F3, A:P4 | 0.897 | Red |
|  | **NSLKSNPFNFKLRHSRAGGNPGAP** | 5-28 | 1.42 | A:G55, A:M57, A:I58 | 0.868 | Green |
| **NP_819523.1 (Q83E43)** | **NGRGGNLER** | 214-222 | 3.05 | A:D49, A:K50, A:K51, A:T52 | 0.916 | Red |
|  | **PIWYDMFGS** | 153-161 | 1.06 |  |  |  |
|  | **SNGRGGNLER** | 213-222 | 2.85 | A:W155, A:Y156, A:D157, A:M158, A:F159, A:G160, A:S161, A:N162 | 0.89 | Green |
|  | **QSGAHTHTKTTSQLKNSRNDCRHIKRYDKKTKHYYNVVVCKHATPPPPSFEEKLQEILSNLTPAQQSLLEKRLARQRQVTKT** | 22-103 | 0.64 |  |  |  |
|  | **AESSDLHNPDIYRY** | 251-264 | 0.67 | A:P68, A:P69, A:S70, A:E72, A:E73 | 0.884 | Blue |
|  | **NGRGGNLER** | 214-222 | 3.05 | A:M1, A:G2, A:K3 | 0.871 | Yellow |
|  | **VESGFKRGA** | 289-297 | 0.75 | A:H28, A:T29, A:K30, A:T31, A:T32, A:S33, A:Q34, A:L35, A:K36, A:N37, A:S38, A:R39, A:N40, A:D41, A:C42, A:R43, A:C61, A:K62, A:H63 | 0.865 | Purple |
|  | **HTKTTSQLKNSRNDCR** | 28-43 | 1.20 |  |  |  |
|  | **WYDMFGSN** | 155-162 | 0.78 | A:E130, A:G131, A:N132, A:T133, A:P134, A:E135, A:S181 | 0.855 | Cyan |
|  | **KAESSDL** | 250-256 | 1.31 |  |  |  |
|  | **EGNTPE** | 130-135 | 2.03 | A:K250, A:A251, A:E252, A:S253, A:S254, A:D255, A:L256, A:H257 | 0.839 | Oranges |
|  | **KRYDKKTKHY** | 46-55 | 1.46 |  |  |  |
| **NP_819354.2 (Q83EK8)** | **SNYSYRTR** | 119-126 | 1.14 | A:G10, A:S12, A:A13, A:C15, A:C16, A:L17, A:S19, A:A20, A:A21, A:F22, A:A23, A:G24, A:G25, A:P26, A:D27, A:I28 | 0.882 | Red |
|  | **MKNRLNGG** | 172-179 | 0.89 |  |  |  |
|  | **LVGSNYSYRTRLG** | 116-128 | 0.84 | A:T59, A:N60, A:G61, A:G62, A:T63, A:V64, A:L65, A:S66, A:A114, A:P115, A:L116, A:V117, A:G118, A:S119, A:S215, A:A216, A:T217, A:A218, A:S219, A:A220, A:E221, A:G222, A:T223, A:A224, A:I225, A:G226 | 0.857 | Green |
|  | **AVSMKNRLNGGIA** | 169-181 | 0.70 |  |  |  |
|  | **DYALYRSKSNSVTLS** | 200-215 | 0.67 |  |  |  |
|  | **QYQYDNVR** | 100-107 | 0.64 |  |  |  |
|  | **TASAEG** | 217-222 | 2.13 |  |  |  |
|  | **SATASAEGTAIG** | 215-226 | 1.35 | A:F36, A:Y86, A:F250 | 0.856 | Blue |
|  | **TNGGTVLS** | 59-66 | 0.79 | A:S90, A:D91, A:W92 | 0.856 | Yellow |
|  | **SALCCLASAAFAGGPDI** | 12-28 | 0.64 | A:M190, A:N191, A:N192 | 0.81 | Purple |
| **NP_820609.2 (Q83B86)** | **KATEDAFKSTAEVSSPPTI** | 42-60 | 0.68 | A:M1, A:K2, A:G3, A:C4, A:K5, A:M6, A:A7, A:E8, A:F9, A:S10, A:K11, A:K12, A:F13, A:L14, A:Q15, A:S16, A:A17, A:K18, A:F19, A:R20, A:V21, A:I22, A:A23, A:A24, A:A25, A:V26, A:A27, A:A28, A:V29, A:A30, A:L31, A:I32, A:A33, A:V34, A:V35, A:G36, A:V37, A:I38, A:W39, A:H40, A:H41, A:K42, A:A43, A:T44, A:E45, A:D46, A:A47, A:F48, A:K49, A:S50, A:T51, A:A52, A:E53, A:V54, A:S55, A:S56, A:P57, A:P58 | 0.924 | Red |
|  | **FEQDQPSAPTTDLKKRNCP** | 111-129 | 0.67 |  |  |  |
|  | **LKEKGC** | 400-405 | 1.01 |  |  |  |
|  | **SSGDCSVKALKKARMAGIS** | 508-526 | 0.76 |  |  |  |
|  | **RHAGYSKGDLLRAGYTAEQAGYPPSSPPGTEVSQSAQRPPLSADNSAASVSGLNNSQSSAMPSINSDSPEA** | 701-771 | 0.60 |  |  |  |
|  | **MQGQMSLQAQKLMAGWSNDSGQAYQVALQQPATTPVGGNVSSQQGAGAAA** | 796-845 | 0.86 |  |  |  |
|  | **AGYSKGDLLRAGYTAEQAGYPPSSPPGTEVSQSAQRPPLSADNSAASVSGLNNSQSSAMPSINSDSPEARLRALQKLQQEQLNEQQRRDVEQQMQGQMSLQAQKLMAGWSNDSGQAYQVALQQPATTPVGGNVSSQQGAGAAAKPTGPVIKA** | 703-854 | 0.70 | A:R738, A:P739, A:P740, A:L741, A:S742, A:A743, A:D744, A:N745, A:S746, A:A747, A:A748, A:S749, A:V750, A:S751, A:G752, A:L753, A:N754, A:N755, A:S756, A:Q757, A:S758, A:S759, A:A760, A:M761, A:P762, A:S763, A:I764, A:N765, A:S766, A:D767, A:S768, A:P769, A:E770, A:A771, A:R772, A:L773, A:R774, A:A775, A:L776, A:Q777, A:K778, A:L779, A:Q780, A:Q781, A:E782, A:Q783, A:L784, A:N785, A:R789 | 0.914 | Green |
|  | **KATEDAFKSTAEVSSPPTIESLPGAGNPSDAYVKTQNIQNAQQASEARKGGTSFVPTITRPSFLGSEDQFEQDQPSAPTTDLKKRNCPIKKVVYMYKPNPASCTVDNLKLARSAGVTAEELVCQSCSCPSLRLAGY** | 42-177 | 0.68 |  |  |  |
|  | **ADDLKAAGFSDQALSAAGFPPSSGDCSVKALKKARMAGISATELKEKGCGLAALKAAGFTAAELKNAGFTAAQLKAAGFSAKDLKDAGFSAAELKAAGFGAKDLKDA** | 487-593 | 0.63 |  |  |  |
|  | **YSSAEMQAAGIQTNNPDCDLAALKKARASGVTAAELRQKGCGLAALKAAGFTAAELKDAGF** | 247-307 | 0.70 | A:A553, A:F575, A:S576, A:A578, A:E579, A:K581, A:A582, A:A583, A:G584, A:F585, A:G586, A:A593, A:Y595, A:A603, A:G604, A:F605, A:S606, A:A607, A:A608, A:Q609, A:L610, A:K611, A:D612, A:A613, A:G614, A:F615, A:D616, A:A617, A:A619, A:L620, A:D622, A:A623, A:G624, A:F625, A:S626, A:A627, A:A628, A:D629, A:L630, A:K631, A:N632, A:A633, A:G634, A:F635, A:S636, A:A637, A:E638, A:A639, A:L640, A:K641, A:N642, A:A643, A:G644, A:F645, A:S646, A:A647, A:A648, A:Q649, A:L650, A:K651, A:A652, A:A653, A:G654, A:F655, A:S656, A:A657, A:G658, A:A659, A:L660, A:K661, A:A662, A:A663, A:G664, A:F665, A:S666, A:A667, A:S668, A:Q669, A:L670, A:K671, A:A672, A:A673, A:G674, A:F675, A:D676, A:A677, A:K678, A:A679, A:L680, A:R681, A:D682, A:A683, A:G684, A:F685, A:S686, A:E689, A:A693, A:A703, A:G704, A:Y705, A:S706, A:D709, A:R712 | 0.861 | Blue |
|  | **VEALKAARAQGISAKELKEKGCGLAALKAAGFTAAELKDAGF** | 384-425 | 0.69 |  |  |  |
|  | **AGFSAADLKAAGFSAAQLKAAGFSAKALKAA** | 433-463 | 0.76 |  |  |  |
|  | **GFSAKDLKAA** | 206-2115 | 1.12 |  |  |  |
|  | **TLPQPLPA** | 335-342 | 1.04 |  |  |  |
|  | **SQLKAA** | 668-673 | 0.66 |  |  |  |

**^*^** **Panel of 27 most frequent A & B alleles:** 1. A*01:01; 2. A*02:01; 3. A*02:03; 4. A*02:06; 5. A*03:01; 6. A*11:01; 7. A*23:01; 8. A*24:02; 9. A*26:01; 10. A*30:01; 11. A*30:02; 12. A*31:01; 13. A*32:01; 14. A*33:01; 15. A*68:01; 16. A*68:02; 17. B*07:02; 18. B*08:01; 19. B*15:01; 20. B*35:01; 21. B*40:01; 22. B*44:02; 23. B*44:03; 24. B*51:01; 25. B*53:01; 26. B*57:01; 27. B*58:01

**^£^ Panel of 26 most frequent alleles:** DRB1*01:01; DRB1*03:01; DRB1*04:01; DRB1*04:05; DRB1*07:01; DRB1*08:02; DRB1*09:01; DRB1*11:01; DRB1*12:01; DRB1*13:02; DRB1*15:01; DRB3*01:01; DRB3*02:02; DRB4*01:01; DRB5*01:01; DPA1*01/DPB1*04:01; DPA1*01:03/DPB1*02:01; DPA1*02:01/DPB1*01:01; DPA1*02:01/DPB1*05:01; DPA1*03:01/DPB1*04:02; DQA1*01:01/DQB1*05:01; DQA1*01:02/DQB1*06:02; DQA1*03:01/DQB1*03:02; DQA1*04:01/DQB1*04:02; DQA1*05:01/DQB1*02:01; DQA1*05:01/DQB1*03:01
