# Supplementary material for: Unveiling promising immunogenic targets in Coxiella burnetii through in silico analysis: paving the way for novel vaccine strategies
Source: BMC Infect Dis. 2023 Dec 21;23:902. doi: 10.1186/s12879-023-08904-7 (PMC10740251; doi:10.1186/s12879-023-08904-7)
Supplement: Supplementary file 6 — Supplementary Material 6 [file 12879_2023_8904_MOESM6_ESM.docx]

**Table S3.** Molecular docking investigations were performed using the Cluspro and PatchDock web tools to explore the interactions between 14 presumed immunogenic proteins derived from *C. burnetii* and human Toll-Like Receptors (TLR-1, TLR-2, TLR-4, and TLR-6).

| **TLR-1** | | | | | |
| --- | --- | --- | --- | --- | --- |
| **UniProt ID** | **Cluspro (Balanced)** | | | **PatchDock (Beta 1.3 Version)** | |
|  | **cluster size** | **weighted score (Center)** | **weighted score (Lowest Energy)** | **Score** | **Negative Atomic Contact Energy (ACE)** |
| Q83F57 | 47 | -727.6 | -896.1 | 13596 | 417.03 |
| Q83EW1 | 98 | -877.8 | -1062.5 | 17700 | 486.38 |
| Q83EK8 | 101 | -1005.5 | -1161.9 | 19100 | 493.91 |
| Q83E43 | 69 | -1405.1 | -1590.6 | 22928 | -83.81 |
| Q83DJ4 | 65 | -821.5 | -821.5 | 18768 | 483.88 |
| Q83D08 | 56 | -865.6 | -1004.4 | 18346 | 371.88 |
| Q83CL9 | 115 | -741.0 | -880.2 | 16200 | 481.97 |
| P39917 | 63 | -737.1 | -826.2 | 16052 | 326.86 |
| Q83BU0 | 154 | -1131.7 | -1401.6 | 19314 | 203.79 |
| Q83BT8 | 126 | -907.9 | -1334.0 | 20030 | -3.57 |
| Q83BB2 | 114 | -968.1 | -1100.9 | 18748 | 401.37 |
| Q83B86 | 50 | -917.7 | -1024.2 | 18072 | 418.25 |
| Q83AQ2 | 69 | -858.9 | -1000.1 | 17150 | 463.04 |
| Q83A32 | 130 | -800.2 | -876.0 | 17682 | 419.40 |
| **Mean** | | -911.8 | -1070.0 | 18120.4 | 348.5 |
| **TLR-2** | | | | | |
| Q83F57 | 41 | -816.5 | -991.4 | 14946 | -263.20 |
| Q83EW1 | 98 | -1230.0 | -1281.7 | 16036 | 454.60 |
| Q83EK8 | 99 | -1153.2 | -1317.9 | 18798 | 462.65 |
| Q83E43 | 89 | -1200.3 | -1232.6 | 22048 | 222.35 |
| Q83DJ4 | 62 | -683.5 | -872.4 | 15524 | 484.40 |
| Q83D08 | 68 | -889.8 | -1038.0 | 15734 | 286.13 |
| Q83CL9 | 199 | -789.6 | -951.4 | 15050 | 454.07 |
| P39917 | 125 | -913.0 | -1038.9 | 15252 | 408.17 |
| Q83BU0 | 92 | -1132.8 | -1334.9 | 20402 | 497.11 |
| Q83BT8 | 138 | -1122.2 | -1269.9 | 19334 | 494.22 |
| Q83BB2 | 86 | -1056.5 | -1171.2 | 18624 | 217.03 |
| Q83B86 | 87 | -1003.0 | -1003.0 | 18152 | 278.93 |
| Q83AQ2 | 84 | -942.5 | -1084.4 | 17646 | 395.31 |
| Q83A32 | 151 | -924.6 | -970.4 | 14764 | 484.41 |
| **Mean** | | -989.8 | -1111.2 | 17307.8 | 348.2 |
| **TLR-4** | | | | | |
| Q83F57 | 33 | -950.3 | -1013.7 | 15568 | 185.73 |
| Q83EW1 | 41 | -992.0 | -1146.6 | 15496 | 442.92 |
| Q83EK8 | 64 | -1148.5 | -1340.7 | 24008 | 415.81 |
| Q83E43 | 50 | -1418.7 | -1619.1 | 19904 | -101.47 |
| Q83DJ4 | 75 | -1050.2 | -1291.0 | 20350 | 155.57 |
| Q83D08 | 40 | -921.7 | -1186.1 | 15194 | 452.81 |
| Q83CL9 | 46 | -758.9 | -903.3 | 18006 | 451.85 |
| P39917 | 83 | -848.8 | -917.3 | 18628 | 474.02 |
| Q83BU0 | 66 | -1496.0 | -1559.3 | 20904 | 220.37 |
| Q83BT8 | 106 | -1243.1 | -1639.7 | 22758 | 439.20 |
| Q83BB2 | 75 | -1270.9 | -1483.3 | 21950 | 302.23 |
| Q83B86 | 28 | -1170.1 | -1223.2 | 21618 | 485.21 |
| Q83AQ2 | 45 | -955.2 | -1042.2 | 20694 | 81.05 |
| Q83A32 | 47 | -796.4 | -930.1 | 18758 | 492.77 |
| **Mean** | | -1072.9 | -1235.4 | 19559.7 | 321.3 |
| **TLR-6** | | | | | |
| Q83F57 | 137 | -866.3 | -978.2 | 13652 | 147.03 |
| Q83EW1 | 94 | -1086.8 | -1286.2 | 20062 | 295.53 |
| Q83EK8 | 238 | -1394.7 | -1532.6 | 19370 | -8.70 |
| Q83E43 | 90 | -1226.6 | -1335.5 | 20366 | 20366 |
| Q83DJ4 | 68 | -869.6 | -1059.6 | 17572 | 266.36 |
| Q83D08 | 79 | -934.3 | -1087.9 | 17570 | 351.21 |
| Q83CL9 | 78 | -857.4 | -945.7 | 16810 | 309.16 |
| P39917 | 148 | -924.3 | -1033.9 | 17144 | 339.93 |
| Q83BU0 | 162 | -1509.8 | -1614.5 | 21490 | 260.23 |
| Q83BT8 | 107 | -1223.9 | -1461.1 | 20160 | 236.63 |
| Q83BB2 | 90 | -1310.7 | -1395.1 | 20332 | 121.82 |
| Q83B86 | 83 | -979.8 | -1104.9 | 21104 | 288.79 |
| Q83AQ2 | 56 | -1049.1 | -1099.6 | 19676 | 176.05 |
| Q83A32 | 133 | -919.2 | -1012.7 | 16294 | 460.69 |
| **Mean** | | -1082.3 | -1210.5 | 18685.8 | 1686.4 |
